# Genetics and epidemiology of mutational barcode-defined clonal hematopoiesis

**DOI:** 10.1038/s41588-023-01555-z

**Published:** 2023-11-06

**Authors:** Simon N. Stacey, Florian Zink, Gisli H. Halldorsson, Lilja Stefansdottir, Sigurjon A. Gudjonsson, Gudmundur Einarsson, Grimur Hjörleifsson, Thjodbjorg Eiriksdottir, Anna Helgadottir, Gyda Björnsdottir, Thorgeir E. Thorgeirsson, Thorunn A. Olafsdottir, Ingileif Jonsdottir, Solveig Gretarsdottir, Vinicius Tragante, Magnus K. Magnusson, Hakon Jonsson, Julius Gudmundsson, Sigurgeir Olafsson, Hilma Holm, Daniel F. Gudbjartsson, Patrick Sulem, Agnar Helgason, Unnur Thorsteinsdottir, Laufey Tryggvadottir, Thorunn Rafnar, Pall Melsted, Magnus Ö. Ulfarsson, Brynjar Vidarsson, Gudmar Thorleifsson, Kari Stefansson

**Affiliations:** 1 deCODE genetics/Amgen Inc., Reykjavik, Iceland; 2 School of Engineering and Natural Sciences, University of Iceland, Reykjavik, Iceland; 3 Faculty of Medicine, School of Health Sciences, University of Iceland, Reykjavik, Iceland; 4 Department of Immunology, Landspitali University Hospital, Reykjavik, Iceland; 5 Department of Anthropology, University of Iceland, Reykjavik, Iceland; 6 Icelandic Cancer Registry, Reykjavik, Iceland; 7 Department of Hematology, Landspitali University Hospital, Reykjavik, Iceland

**Keywords:** Genome-wide association studies, Myelodysplastic syndrome, Myelodysplastic syndrome, Ageing

## Abstract

Clonal hematopoiesis (CH) arises when a substantial proportion of mature blood cells is derived from a single hematopoietic stem cell lineage. Using whole-genome sequencing of 45,510 Icelandic and 130,709 UK Biobank participants combined with a mutational barcode method, we identified 16,306 people with CH. Prevalence approaches 50% in elderly participants. Smoking demonstrates a dosage-dependent impact on risk of CH. CH associates with several smoking-related diseases. Contrary to published claims, we find no evidence that CH is associated with cardiovascular disease. We provide evidence that CH is driven by genes that are commonly mutated in myeloid neoplasia and implicate several new driver genes. The presence and nature of a driver mutation alters the risk profile for hematological disorders. Nevertheless, most CH cases have no known driver mutations. A CH genome-wide association study identified 25 loci, including 19 not implicated previously in CH. Splicing, protein and expression quantitative trait loci were identified for *CD164* and *TCL1A*.

## Abstract (web summary)

A barcode-based approach applied to UK Biobank and an Icelandic cohort identifies drivers of clonal hematopoiesis (CH) and finds associations between CH and multiple diseases. Genome-wide association analyses identify 25 loci associated with CH susceptibility.

## Main

Clonal hematopoiesis (CH) may be defined as a disproportionate expansion of one or a few clones of hematopoietic stem cells (HSCs) in individuals with ostensibly normal hematopoiesis^1^. Hematopoiesis has a highly polyclonal underpinning in younger individuals, but becomes increasingly restricted in HSC clonal diversity with advancing age^2^. CH is associated with reduced age-adjusted life expectancy and predisposes to hematological neoplasia, particularly to myeloid diseases^1,3,4^. CH has also been implicated in a broad spectrum of nonhematological conditions, ranging from carcinomas to cardiovascular disease (CVD)^1,5–9^.

Peripheral blood sampling can provide a reasonable insight into the clonal makeup of the recent underlying HSC population. Leukocytes from normal blood are predominantly short-lived myeloid cells, mostly granulocytes. These cells have high production rates and short time lags from committed progenitor cells, which in turn require continual replenishment from HSC or multipotent progenitors^10^. Naturally, the lymphocytic lineages have a much greater time lag from the underlying HSC population. Clonal expansions in CH can show multilineage involvement extending to lymphocytes, but do not always do so^11,12^.

Perhaps as a result of the proximity of myeloid lineages to the underlying HSC population, somatic mutations that initiate myeloid malignancies are thought to arise in the HSC compartment. Similar mutations can be found in apparently normal but clonally expanded hematopoietic cells from individuals who appear to be well. In both cases, the mutations can be traced back to underlying HSC^12^. We refer to them as ‘candidate preleukemic driver’ (CPLD) mutations, because of their propensity to drive CH expansions and consequently to increase risks of hematological disease. Indeed, the presence of a CPLD mutation in a blood sample from an evidently healthy individual has, by many investigators, been used to define the presence of CH^4,13–15^. Clearly, and as pointed out by others^16^, this biases the detection of CH in favor of genes and mutations that may subsequently lead to the development of myeloid neoplasia.

As cell populations grow they accumulate mutations, most of which are presumed to be phenotypically inconsequential. As a result, every clone is uniquely ‘barcoded’ by the somatic mutations that were present in the founder cell at the inception of the clone. If a particular clone expands sufficiently, its mutational barcode becomes evident in DNA sequence reads. We have shown through whole-genome sequencing (WGS) of peripheral blood that clonal expansions indicative of CH can be detected by examining counts of mosaic somatic mutations (if sufficient care is taken to differentiate them from germline variants and sequencing errors)^1^. Thus CH expansions can be identified solely on the basis of barcode mutations, irrespective of whether they carry a CPLD mutation. This method enabled us and others to show that CH is very common, if not inevitable, in the elderly^1–3^. Moreover, most CH cases do not carry an obvious CPLD mutation. Here we use mutational barcodes to study the epidemiology and genetics of CH in participants from Iceland (ISL) and the UK Biobank (UKB) for whom we have generated extensive WGS data.

## Results

### Identification of CH cases from WGS in ISL and UKB

**Extended Data Fig. 1:**
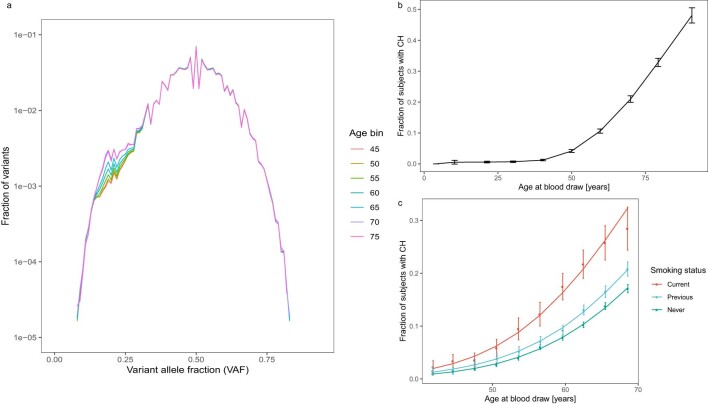
Age and smoking dependency of CH. **a**, Frequency distribution in UKB of singleton mutations: Mutations that were observed only once in the cohort were plotted by variant allele fraction (VAF). The counts were further stratified by the age of the subject at blood draw. Note that there is a ‘bump’ in the distribution starting below a VAF of approximately 0.3 and that the size of this ‘bump’ is age dependent. This distribution was modeled to identify people with more than the expected number of low-VAF mutations, as explained further in the Methods. **b**, Proportion of subjects with CH increases with age. The line connects the observed CH proportions, error bars are 95%CI. Data are from the ISL sample (n = 45,510), which has a larger age range than UKB. **c**, Effects of current and previous smoking on CH by age: CH was modeled by age and stratified by current or previous smoking status using sex, *Pack-Years* and *Years Since Stopped Smoking* as covariates. Points correspond to observed CH proportions and error bars are 95%CI. Lines correspond to a logistic regression fit. Data are from the UKB sample (n = 130,709).

We used WGS from 45,510 Icelanders and 130,709 British ancestry participants from the UKB^17,18^. Average sequencing depth was 33× for UKB and 38× for ISL. Participants with prior diagnoses of hematological disorders or grossly abnormal hematology measurements on entry were excluded. We identified people with CH based on an evolution of our mutational barcode strategy^1^. Mosaic somatic mutation barcodes were generated by modeling low variant allele fraction (VAF) sequence reads (Extended Data Fig. 1). To reduce contamination from low-VAF germline variants and recurrent sequencing errors, we used only indicator mutations that were observed once in each cohort and restricted in VAF range to 0.10–0.25. Participants with barcodes containing a number of indicator mutations above a threshold were considered to have CH. We identified 16,306 people with CH, a prevalence over the two cohorts of 9.3%.

As anticipated from previous studies, CH was uncommon in under 45-year-olds, but increased dramatically in frequency thereafter, approaching 50% by age 80. Both current and previous smoking substantially increased risk of CH (Extended Data Fig. 1b,c). Pack years further increased CH risk (*P* = 8.57 × 10^−7^), whereas years since stopped smoking were protective (*P* = 3.54 × 10^−10^; Supplementary Table 1), indicating a dose-dependent relationship between smoking and CH. While the mechanisms by which age and smoking promote CH are yet to be elucidated, both factors clearly are potential confounders in epidemiological analyses. Participants with CH were at substantially greater risk of all-cause mortality and of being diagnosed subsequently with a hematological disorder. Smoking was an independent risk factor for mortality but not for hematological disorders (Supplementary Table 2).

### Associations of CH with disease

In case–control analysis, CH had strong associations with both myeloid and lymphoid neoplasia (Table 1 and Supplementary Table 3). CH was also associated with existing or subsequent diagnoses of chronic obstructive pulmonary disease (COPD), lung cancer, peripheral artery disease (PAD), emphysema and alcohol abuse. These nonhematological conditions are known to be smoking-related, and their significance was substantially attenuated once smoking was taken into account. This suggests that the associations may be due to residual confounding from various aspects of smoking behavior. Hematological disorder associations were not similarly attenuated by smoking adjustments. Analysis restricted to never smokers produced similar conclusions (Supplementary Table 4).

**Table 1:** Associations between clonal hematopoiesis and disease in UKB

Phenotype UKB	*n* cases	*n* controls	OR^a^	*P* value^a^	OR_adj_ smoking^b^	*P*_adj_ smoking^b^
C91 lymphoid leukemia	268	124,500	10.44	1.59 × 10^−64^	10.62	6.51 × 10^−62^
C911 chronic lymphocytic leukemia	229	124,010	11.94	2.59 × 10^−63^	12.25	9.27 × 10^−61^
Myeloproliferative neoplasms	194	124,670	7.62	1.60 × 10^−34^	7.92	1.40 × 10^−34^
C92 myeloid leukemia	182	124,057	7.60	1.05 × 10^−33^	7.41	3.85 × 10^−31^
F10 mental and behavioral disorders due to use of alcohol	3,069	121,730	1.91	6.32 × 10^−28^	1.58	9.53 × 10^−14^
D46 myelodysplastic syndromes	141	124,098	6.40	7.12 × 10^−23^	6.47	1.31 × 10^−21^
D473 essential hemorrhagic thrombocythaemia	183	124,056	5.32	7.71 × 10^−21^	5.23	9.48 × 10^−20^
J44 other chronic obstructive pulmonary disease	4,113	120,751	1.51	2.01 × 10^−19^	1.12	0.018
D45 polycythemia vera	92	124,676	8.28	1.05 × 10^−18^	8.12	3.29 × 10^−18^
C34 malignant neoplasm of bronchus and lung	1,377	123,391	1.90	2.29 × 10^−18^	1.45	1.00 × 10^−6^
C93 monocytic leukemia	25	123,134	46.51	1.01 × 10^−16^	47.72	9.37 × 10^−17^
Peripheral artery disease	2,012	122,787	1.60	9.23 × 10^−14^	1.27	2.46 × 10^−4^
D619 aplastic anemia	284	123,955	2.69	1.28 × 10^−10^	2.38	1.00 × 10^−7^
D474 osteomyelofibrosis	26	123,133	13.56	9.08 × 10^−10^	14.61	4.71 × 10^−9^
J43 emphysema	1,025	123,774	1.70	1.01 × 10^−9^	1.18	0.066
C83 diffuse non-Hodgkins lymphoma	359	124,536	2.29	2.73 × 10^−9^	2.23	3.52 × 10^−8^
K709 alcoholic liver disease	276	124,492	2.40	4.70 × 10^−6^	1.93	6.20 × 10^−4^
I50 heart failure^c^	2,922	121,942	1.28	5.03 × 10^−6^	1.17	0.0045

The Bonferroni cutoff level is 5.00 × 10^−6^, unadjusted.

Phenotype list is edited to remove redundancies and subphenotypes.

^a^Multivariable regression, adjusted for sex and age at blood draw (linear and quadratic).

^b^Additionally, adjusted for smoking status (current, previous), pack years and years since stopped smoking.

^c^Heart failure was included in the UKB table because prior literature reports implicated an association with CH.

Case–control analysis revealed no indication of association between CH and key CVD phenotypes, neither in UKB nor in ISL (Supplementary Table 5). Unadjusted for smoking, no CVD phenotype passed Bonferroni significance and, once adjusted, none was even nominally significant. To examine this further, we conducted a time-to-CVD-event analysis in UKB. We considered also whether CH defined by mutational barcodes differed in this respect from CH containing a CPLD mutation. Additionally, we examined CHIP as defined using the filtering strategy recommended in ref. ^19,20^. In all three instances, we were unable to measure any increased risk of CVD in people with CH. We did, though, observe strong effects from potential confounders in the multivariable model (Table 2). CH has also been implicated in pro-inflammatory phenomena, a suggested basis for its reported CVD association^21,22^. Accordingly, we looked for CH associations with a panel of inflammatory conditions, but saw none (Supplementary Table 5). In UKB, CH was associated with alcoholic liver disease (Table 1) but not fatty liver conditions, at variance with a recent report^23^.

**Table 2:** Time-to-event analysis of three models of CH for cardiovascular disease endpoints^a^

Characteristics	Barcode-CH			CPLD-CH^b^			CHIP^c^		
	HR	95% CI	*P* value	HR	95% CI	*P* value	HR	95% CI	*P* value
Clonal hematopoiesis	1.01	(0.94, 1.08)	0.88	1.01	(0.90, 1.13)	0.89	1.01	(0.88, 1.15)	0.92
Age at blood draw	1.08	(1.06, 1.10)	<2 × 10^−16^	1.08	(1.06, 1.10)	<2 × 10^−16^	1.08	(1.06, 1.10)	<2 × 10^−16^
Previous smoking	1.15	(1.10, 1.22)	8.60 × 10^−8^	1.16	(1.10, 1.22)	8.20 × 10^−8^	1.16	(1.10, 1.22)	8.20 × 10^−8^
Current smoking	2.10	(1.95, 2.27)	<2 × 10^−16^	2.10	(1.95, 2.27)	<2 × 10^−16^	2.10	(1.95, 2.27)	<2 × 10^−16^
Hypertension	1.44	(1.37, 1.51)	<2 × 10^−16^	1.44	(1.37, 1.51)	<2 × 10^−16^	1.44	(1.37, 1.51)	<2 × 10^−16^
BMI	1.05	(1.04, 1.05)	<2 × 10^−16^	1.05	(1.04, 1.05)	<2 × 10^−16^	1.05	(1.04, 1.05)	<2 × 10^−16^

*n* = 118,673; number of events = 7,242; stratified by age bin and sex.

^a^Data are from UKB.

^b^CH containing a CPLD mutation, defined using our in-house methodology (Methods).

^c^CHIP is defined using the strategy described in ref. ^20^.

CI, confidence interval from Cox regression.

To better understand the increased mortality rate attributable to CH, we examined the primary cause of death records in a meta-analysis of ISL and UKB. Participants with CH were at increased risk of death from both myeloid and lymphoid hematological disorders, as well as lung cancer, COPD and alcohol abuse (Supplementary Table 6). As before, the nonhematological risks were attenuated (but not eliminated) by adjustment for smoking. Chronic ischemic heart disease and heart failure had nominally significant hazard ratios (HRs), but did not meet the Bonferroni threshold. Even though a substantial number of deaths from acute myocardial infarction occurred in the cohort, their frequency was not elevated in participants with CH.

### Association of mosaic somatic mutations with CH

**Fig. 1:**
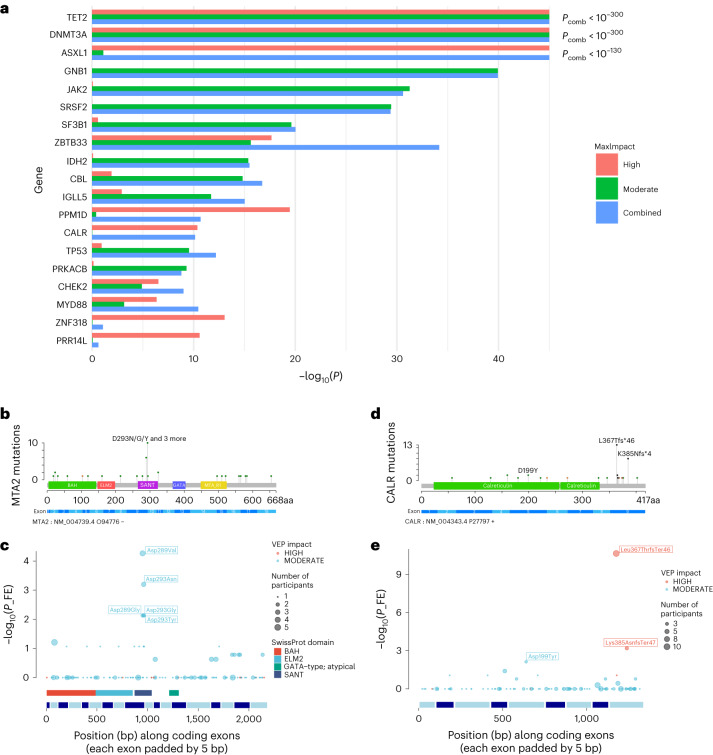
Association of mosaic somatic mutations with CH. **a**, Results (−log_10_(*P*)) of gene-based burden test using SKAT-O for association of somatic mutations with CH. Data are a meta-analysis of ISL and UKB. Separate burden tests were conducted to include high-impact (red) or moderate-impact mutations (green; as assessed with the Ensembl VEP) and a combination of both types (blue) for the genes indicated. *P*_comb_ is the *P* value for combined high- and moderate-impact variants. The maximum impact (MaxImpact) VEP annotation was used to classify each mutation. **b**, Lollipop plot showing the counts of somatic mutations in the *MTA2* gene detected in CH cases in UKB. Green lollipops are missense, black are frameshifts and orange are splice mutations. PFAM domain and exon structures are shown below. BAH, bromo-adjacent homology domain; ELM2, Egl-27 and MTA1 homology 2 domain; GATA, GATA zinc finger domain; MTA_R1, metastasis-associated protein MTA1 R1 domain; SANT, Swi3-Ada2-N-Cor and TFIIIB domain. **c**, Fisher’s exact association test results in UKB for individual mutations in *MTA2*. Diameter of the circles indicates the total number of participants with the mutation (CH cases + controls). SwissProt domains and exon structure of the gene are shown below. **d**,**e**, As in **b** and **c** but for the *CALR* gene. FE, Fisher’s exact.

Most prior DNA sequence-based studies identified CH using a predefined list of CPLD mutations that are already known to occur in myeloid neoplasia^4,13–15^. Some studies have tested mutated genes for statistical association with CH or evidence of positive selection in CH^1,3,24,25^. Our method can identify CH irrespective of whether a CPLD mutation is present. Thus we can search in a comparatively unbiased manner for genes with mutations that drive CH. We conducted a gene-based burden test for somatic mutations associated with CH (Fig. 1a and Supplementary Table 7). As anticipated from previous studies^1,3,4^, mutations in *DNMT3A*, *TET2* and *ASXL1* were the most significantly associated with CH. Most of the other genes are known to be commonly mutated in myeloid disease. Some are implicated, additionally or uniquely, in lymphoid neoplasia^26^.

We also examined the intragenic distribution of the somatic mutations and used Fisher’s exact tests to identify individual mutations that drive the signal from each gene (Fig. 1b–e and Supplementary Fig. 1)*. ASXL1* exhibited predominantly frameshift or nonsense mutations in the 13th (last) exon. *ASXL1* activation in myeloid neoplasia typically results from gain-of-function mutations that produce C-terminally truncated proteins^27^. However, we also saw protein truncation mutations in exon 12, namely Arg404Ter and Arg417Ter, that associated strongly with CH (*P* = 9.7 × 10^−6^ and 2.6 × 10^−6^, respectively, UKB, Fisher’s exact test). These mutations are puzzling because they would be expected to induce nonsense-mediated decay of the *ASXL1* transcript^28^, which would obviate a gain-of-function effect. Further investigation is warranted. The CH association with *GNB1* was completely attributable to Lys57Glu mutations (*P* = 1.4 × 10^−46^, UKB, Fisher’s exact test). *GNB1* mutations affecting Lys57 predominate in myeloid neoplasia, whereas mutations at other positions are more frequent in lymphoid malignancies^29^. In *CALR*, high-impact mutations clustered in the ninth (last) exon, suggesting a gain-of-function analogous to that seen in *PPM1D* and *ASXL1* (Fig. 1d,e). Such mutations are present in essential thrombocythemia (ET) and primary myelofibrosis^30^; however, they have not been consistently implicated as CH-defining mutations (Supplementary Table 7). We obtained robust evidence linking high-impact *PRR14L* mutations to CH (*P* = 3 × 10^−11^, UKB, SKAT-O). *PRR14L* is not generally recognized as a CH gene (Supplementary Table 7); however, mutations have been seen in chronic myelomonocytic leukemia and infrequently in CH participants^31^.

**Extended Data Fig. 2:**
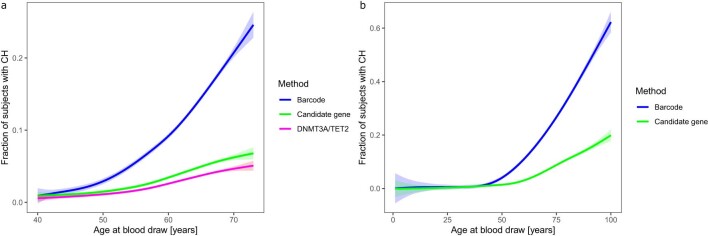
Only a minority of CH cases have a known CPLD mutation. The proportion of subjects with barcode-CH by age is shown in blue. Proportions of subjects where a CPLD mutation had been identified (CPLD-CH) are in green and the proportion with a mutation in DNMT3A or TET2 are in magenta. CPLD mutations were defined as in ref. ^13^. The lines indicate a data fit using a generalized additive model with cubic splines. Shading indicates 95%CI. **a**, Data from UKB. **b**, Data from ISL.

We previously reported a tentative association between CH and *MYD88* mutations in ISL^1^. We confirm that finding robustly here (*P* = 1.9 × 10^−10^, UKB, SKAT-O), the strongest signal coming from Leu252Pro. *MYD88* Leu252Pro (formerly Leu265Pro) mutations are particularly related to lymphoplasmacytic lymphoma/Waldenström macroglobulinemia (LPL/WM), which would not be expected to have a substantial bloodborne component^26,32,33^. However, *MYD88* mutations also occur in an atypical minority of chronic lymphocytic leukemia (CLL) and Leu252Pro has been observed in normal B cells from patients with LPL/WM^34,35^. We also reported a CH association with mutations in *MTA2* (ref. ^1^) and confirm that finding here (*P* = 7.9 × 10^−7^, UKB, SKAT-O). Individually significant missense mutations were clustered within the SANT domain (Fig. 1b,c), which recruits histone deacetylase-1 to the nucleosome remodeling and deacetylase (NuRD) complex^36^. Even though we were able to demonstrate strong associations between the common CPLD genes and CH, most cases could not be accounted for by an obvious driver mutation (Extended Data Fig. 2). Several factors may contribute to this; a lower sensitivity for CPLD mutation detection in WGS versus whole exome or panel sequencing, driver mutations located outside the coding sequences of known CPLD genes, mosaic chromosomal alterations (mCA), clonally inherited epigenetic effects and random drift in an HSC pool with a very low effective population size^1,2^.

### Differential risks of hematological disorders

**Fig. 2:**
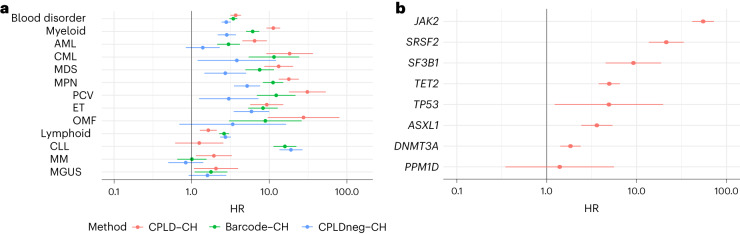
Differential risks of subsequent hematological disorders for barcode-CH, CPLD-CH and CPLDneg-CH. **a**, HR and 95% CI from Cox regressions for subtypes of hematological disorder, stratified by CPLD-CH, barcode-CH and CPLDneg-CH. Diagnoses were included if they arose 6 months or more after blood sampling for CH determination. Data are meta-analysis of UKB and ISL (*n* = 162,963 participants overall, 14,837 with barcode-CH, 5,288 with CPLD-CH and 11,692 with CPLDneg-CH). **b**, HR and 95% CI for subsequent hematological disorder stratified by CPLD genes. MM, multiple myeloma; MGUS, monoclonal gammopathy of undetermined significance; OMF, osteomyelofibrosis.

We investigated the types of hematological disorders arising in participants with CH. Moreover, we considered how the risk profile of CH defined by mutational barcodes (referred to herein as simply ‘CH’ or ‘barcode-CH’ when disambiguation is required) differed from CH defined by the presence of a CPLD mutation (CPLD-CH) or by the absence of a CPLD mutation in a barcode positive case (CPLDneg-CH) (Supplementary Table 8). As shown in Fig. 2a, HRs for both myeloid and lymphoid disorders were increased for all three CH classes. There were, however, differences in nuance. Participants with CPLD-CH were more likely to develop myeloid neoplasia than those with barcode-CH or CPLDneg-CH. Conversely, participants with barcode-CH or CPLDneg-CH were more likely to develop lymphoid neoplasia than those with CPLD-CH. Within myeloid subtypes, CPLDneg-CH participants were at demonstrable risk of chronic myeloid leukemia (CML), myelodysplastic syndrome (MDS) and myeloproliferative neoplasia (MPN). However, CPLD-CH participants were at higher risk of developing acute myeloblastic leukemia (AML), MDS and MPN (in particular, polycythemia vera (PCV)) than CPLDneg-CH participants. Within lymphoid subtypes, barcode-CH and CPLDneg-CH carried significant risks of CLL, whereas CPLD-CH did not. This suggests that some barcode-CH cases may have incipient, undiagnosed CLL or high-count monoclonal B cell lymphocytosis (MBL). However, because B cells normally comprise a small proportion of the leukocyte population, even in MBL, B cell clonal expansions are unlikely to pass our CH detection threshold in the absence of an overt hematological abnormality. Accordingly, they are unlikely to account for a substantial number of barcode-CH cases. Moreover, associations with MPN and CLL could be driven by undetected mCA accompanying the barcode-CH^37,38^.

We investigated whether, among CPLD-CH participants, risks of hematological disorders differed by the particular CPLD gene involved (Fig. 2b). Significant HRs were seen for *ASXL1*-CH, *DNMT3A*-CH, *JAK2*-CH, *SF3B1*-CH, *SRSF2*-CH, *TET2*-CH and *TP53*-CH but not for *PPM1D*-CH. The risk from *JAK2*-CH was greater than from any other of the CPLD genes. While participants with *DNMT3A*-CH were at somewhat increased risk, HR estimates for other CPLD-CH types including *ASXL1*-CH and *TET2*-CH were substantially higher.

### CH GWAS meta-analysis in ISL and UKB

**Fig. 3:**
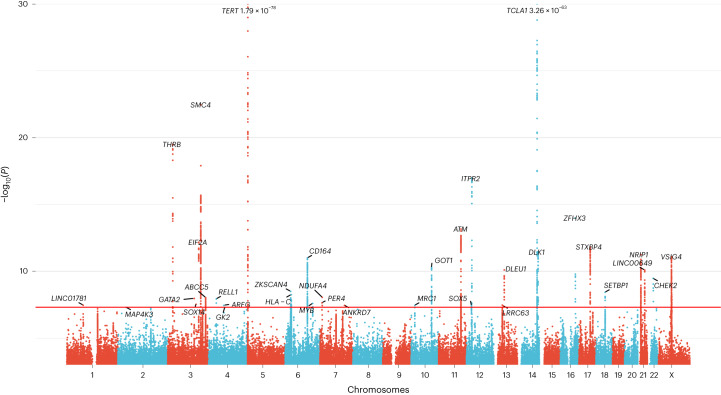
GWAS meta-analysis of barcode-CH in ISL and UKB. Manhattan plot showing logistic regression GWAS results (−log_10_(*P*) versus chromosomal position) from 16,306 cases and 159,913 controls. The horizontal red line corresponds to a *P* value of 5 × 10^−8^. Named loci have unconditional *P* values of <5 × 10^−8^. Loci are named by the nearest gene or plausible candidate. The *TERT* and *TCL1A* loci are offscale, and their *P* values are indicated on the plot. Detailed data for named loci are in Supplementary Table 9. Several high-effect, rare variants were deemed to require further confirmation and were not considered further (indicated in Supplementary Table 9).

**Extended Data Fig. 3:**
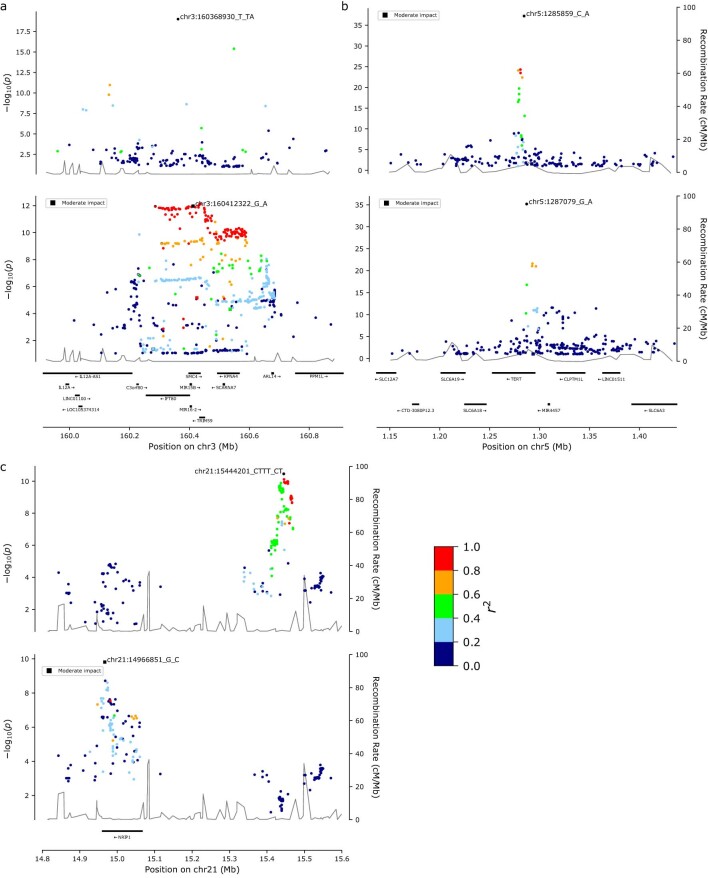
Locus zoom plots for loci where a secondary signal was detected by conditional analysis. Plots show conditional logistic regression GWAS results (−log_10_*P* vs chromosomal position) from 16,306 cases and 159,913 controls. The adjusted signals are shown, with the primary signal in the upper part of each panel and the secondary signal in the lower part. r^2^ values relative to the peak signal are shown by color as indicated in the color bar, bottom right. **a**, *SMC4* locus. **b**, *TERT* locus. **c**, *NRIP1* locus.

We carried out a GWAS meta-analysis for barcode-CH (designated the ‘CH GWAS’) in 130,709 UKB and 45,510 ISL participants, using germline genotypes imputed from WGS training sets^17,18^. We identified 25 loci with association signals of *P* < 5 × 10^−8^ (Fig. 3 and Supplementary Table 9). An additional ten low-frequency, high-effect variants require confirmation and were not considered further. All of the sentinel variants had low variant effect predictor (VEP) impacts. At chr22q12, the sentinel variant was in high linkage disequilibrium (LD) (*r*^2^ = 0.95 in UKB and 1.0 in ISL) with the well-known oncogenic ‘1100delC’ *CHEK2* frameshift mutation rs555607708_delG (Thr367MetfsTer15)^39^. Conditional analysis identified secondary signals at chr3q25 (a splice region variant in *SMC4*), chr5p15 (*TERT*) and chr21q11 (an Arg448Gly missense in *NRIP1*; Extended Data Fig. 3 and Supplementary Table 9). Scanning at a more relaxed stringency (*P* < 5 × 10^−7^) for variants with moderate or high VEP effects identified a low-frequency protective Arg684Gln variant in *RTEL1* (rs35640778_A; odds ratio (OR) = 0.80, *P* = 1.75 × 10^−7^) and a Thr343Ser missense in *ELF1* (rs1056820_T; OR = 0.92, *P* = 1.71 × 10^−7^).

One CH GWAS variant, at *TERT*, was reported by us previously in association with barcode-CH in ISL^1^. We reproduced this association; however, the sentinel *TERT* variant this time was rs7705526_A (OR = 1.28, *P* = 1.79 × 10^−78^), which is the same variant as subsequently reported for CPLD-CH^13^. Several other CH GWAS loci have been associated with related phenotypes, such as CPLD-CH^13–15^, mCA^38,39^, loss of Y chromosome (LoY)^40–42^ or MPN^43,44^. The LD between our CH GWAS variants and those signals is detailed in Supplementary Table 10. We found no previous reports for 19 of the CH GWAS loci.

**Extended Data Fig. 4:**
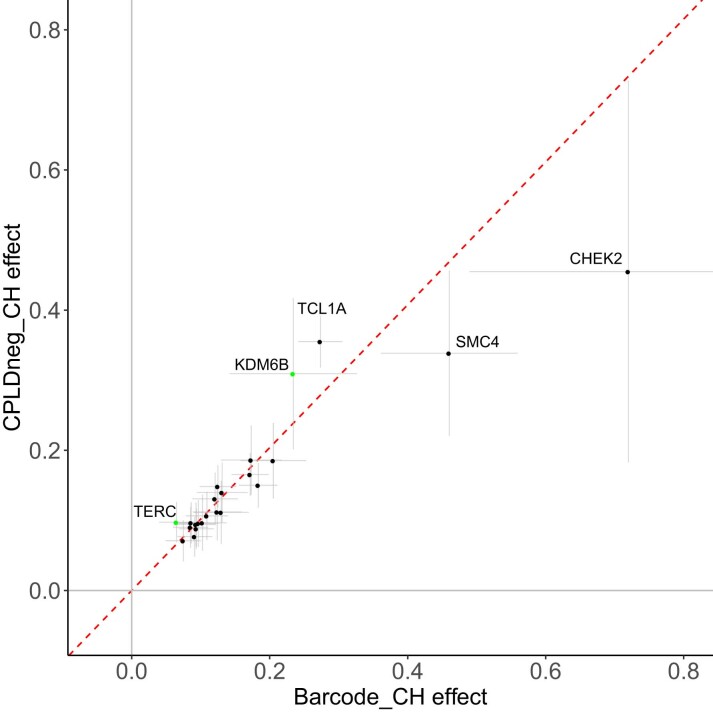
GWAS of CPLDneg-CH and comparison of effects with barcode-CH GWAS. Data are a meta-analysis of ISL and UKB. GWAS variants were included if they were significantly associated with barcode-CH or CPLDneg-CH. The plotted points are association effect estimates (log_e_ odds ratio) and 95%CI from logistic regression association testing for variants in barcode-CH (16,306 cases, 159,913 controls) and CPLDneg-CH (11,692 cases, 151,277 controls) respectively. The fitted inverse variance weighted linear regression, fixed through the origin, is shown as a red dotted line. Variants that were newly discovered in the CPLDneg-CH GWAS are colored green. Labeled loci are discussed in the text.

To gain further insight into CH without known drivers, we repeated the GWAS using only CPLDneg-CH participants as cases (Extended Data Fig. 4 and Supplementary Table 11). Effects were broadly similar to the barcode-CH GWAS (*m* = 1.02, *P* = 1.47 × 10^−18^). Following two new loci were detected: *TERC* and *KDM6B*. The protective effect of chr14:*TCL1A* rs2887399_T was stronger in CPLDneg-CH, perhaps due to the differing effects of this allele in various CPLD mutation backgrounds (see *CPLD* gene specific CH GWAS associations, below). *CHEK2* and *SMC4* variants had somewhat larger effects in barcode-CH.

### CPLD gene-specific CH GWAS associations

**Extended Data Fig. 5:**
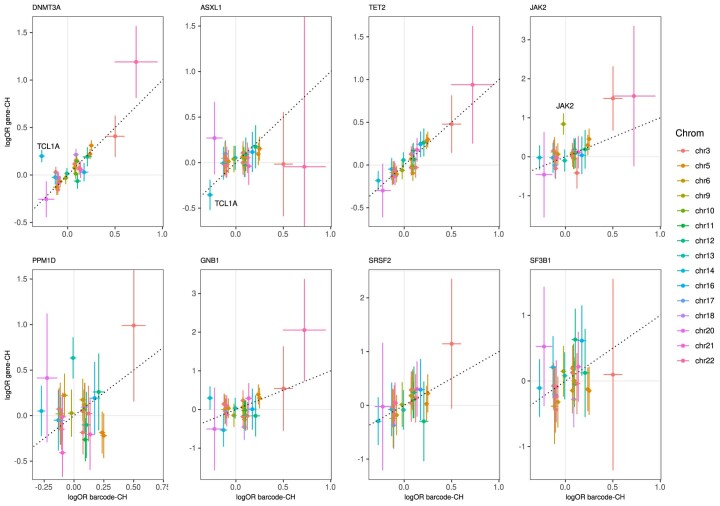
Effects of GWAS meta-analysis variants on various types of CPLD-CH vs barcode-CH. GWAS variants were included if they were significantly associated with barcode-CH or any of the CPLD-CH types. The x-axes show the effects (log_e_ odds ratio) and 95%CI (horizontal lines) for each variant in barcode-CH, determined by logistic regression. The y-axes show the corresponding effects and 95%CI (vertical lines) for each variant in the different types of CPLD-CH, as indicated above each panel. The dotted line shows the position of the diagonal. Gray lines indicate the position of no effect. Detailed data including case and control numbers are in Supplementary Table 12. The chr14:*TCL1A* rs2887399_T allele was *protective* against barcode-CH, *TET2*-CH and *ASXL1*-CH whilst the same allele *increased* risk of *DNMT3A*-CH, in line with previous reports. The chr14:*TCL1A* variant is indicated in the *DNMT3A*-CH and *ASXL1*-CH panels to illustrate the reversal of effect. Similarly, the *chr6:CD164* chr6:*CD164* rs3056655_A allele *increased* risk of barcode-CH and *DNMT3A*-CH but *decreased* risk of *TET2*-CH^13,14^. The latter result was seen only in UKB, whereas ISL data could not confirm it. The chr3:*SMC4* rs201009932 variant had no discernible effect on *ASXL1*-CH while it had a pronounced effect on *JAK2*-CH. chr3:*THRB* had no apparent effect on *DNMT3A*-CH and chr5:*TERT* rs7705526 had no effect on *PPM1D*-CH. Other variants showed prominent effects only in specific CPLD-CH types: chr12:*SOX5* and chr14:*DLK1* had no evident effects outside of barcode-CH, while chr13:*KLF12* had no apparent effect outside of *PPM1D*-CH. The chr9:*JAK2* rs16922785_G allele (indicated in the *JAK2*-CH panel) only conferred CH risk in the context of the *JAK2* Val617Phe somatic mutation and was preferentially linked to it in *cis*, as has been noted previously for the 46/1 *JAK2* haplotype and MPN risk^104^. rs16922785 is in moderate LD with the 46/1 haplotype (r^2^ = 0.68) and had a somewhat stronger association with *JAK2*-CH than the 46/1 haplotype tagger rs12343867_C (*P* = 1.60 × 10^−9^ *vs* 1.04 × 10^−7^).

We repeated the GWAS meta-analysis on CPLD-defined CH for driver genes where there was sufficient power to do so. Considering all variants that were significantly associated with barcode-CH or any one of the CPLD-CH types, we compared their effects on barcode-CH and various types of CPLD-CH. There were substantial differences in effects between CPLD-CH types (Extended Data Fig. 5 and Supplementary Table 12).

Viewing the patterns overall, most variants demonstrated no effect on *ASXL1*-CH. While *TET2*-CH, for example, showed a highly significant slope when regressed on barcode-CH (*m* = 0.94, *P* = 5.64 × 10^−10^), the slope for *ASXL1*-CH versus barcode-CH was much shallower and of lower significance (*m* = 0.41, *P* = 8.76 × 10^−4^). Moreover, *PPM1D*-CH produced no significant regression against barcode-CH. One possible explanation is that environmental factors have a greater influence on *ASXL1*-CH and *PPM1D*-CH than on other CPLD-CH types— risk of *PPM1D*-CH was substantially increased in patients who have undergone chemotherapy (OR = 7.9, *P* = 4.5 × 10^−4^; Supplementary Table 13), while *ASXL1*-CH was more strongly associated with smoking than other CPLD-CH types (Supplementary Table 14) in agreement with previous reports^9,45,46^.

### CH GWAS variants affect blood traits, telomeres and MPN

**Extended Data Fig. 6:**
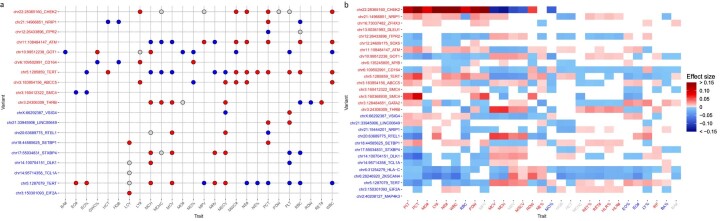
Effects of CH GWAS variants on clinical hematology parameters. **a**, GWAS Catalog reports: For each sentinel CH GWAS variant, we identified all variants in LD with r^2^ > = 0.8 within +/−500kb. For those variants, we searched the GWAS Catalog for reported associations with *P*-values < 1 × 10^−7^ from linear regression association. CH GWAS loci (y-axis) are colored red if the Alt allele increased CH risk, otherwise blue. Circles are colored red if the Alt allele was associated with an increase in the hematological trait value (x-axis), blue if there was a decrease and gray if the direction of effect could not be ascertained. **b**, Associations from linear regression between sentinel CH GWAS variants and clinical hematology traits measured on contemporaneous samples in the UKB: CH GWAS loci (y-axis) are colored red if the Alt allele increased CH risk, otherwise blue. Hematological trait symbols (x-axis) are colored red if their values increased in association with the CH phenotype, blue if they decreased in CH and gray if they were not associated with CH. Blocks are colored in if the effect of the CH GWAS variant on the trait was at least nominally significant: red indicates that the Alt allele was associated with an increase in the hematological trait value, blue indicates a decrease. Intensity of color indicates the effect size. Hematological traits are ordered by hierarchical clustering within the CH at-risk and CH protective strata. Platelet parameters were affected by the greatest number of variants: PCT, PLT, PDW and MPV; followed by erythrocytic parameters MCH, RBC and MCV. The best alignments in direction of effects (*that is* where the effects of the variant on CH and the hematological trait were consistent with the phenotype:phenotype association) were seen again for platelet parameters PDW, PCT and PLT as well as for MO#, LY# and BA%. From the perspective of the CH GWAS variants, the variants affecting the most hematological traits were chr6:*CD164* and chr6:*HLA-C*. However chr6:*CD164* had rather poor alignment in the direction of effects. The best alignments were seen for chr21:14966851 *NRIP1,* chr3:*THRB* and chr3:16068930:*SMC4*. Clinical hematology parameters are as defined in Sheard^47^.

To gain insight into the functionality and pleiotropic effects of the CH GWAS variants, we examined published GWAS associations for them and variants in LD (Supplementary Table 15). Even though participants with grossly abnormal hematology had been excluded from the study, many clinical hematology parameters^47^ showed associations with the CH phenotype. Moreover, many CH GWAS loci had associated clinical hematology traits in the GWAS Catalog or UKB data (Supplementary Tables 15 and 16 and Extended Data Fig. 6).

**Fig. 4:**
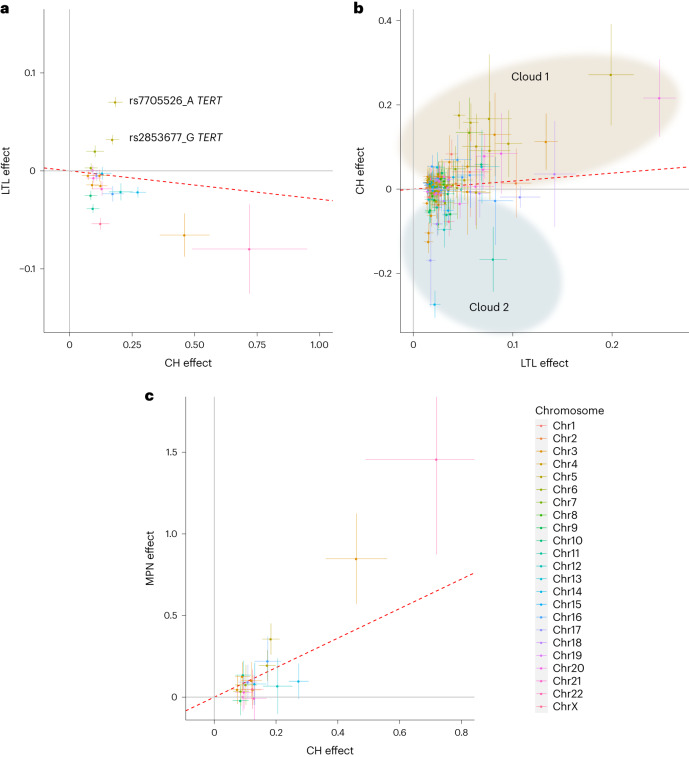
Effects CH GWAS variants and LTL GWAS variants on CH, LTL and MPN outcomes. **a**, Effects of CH GWAS variants on CH (*x* axis) and LTL (*y* axis) outcomes. LTL data are from UKB (*n* = 418,251). The two discordant *TERT* variants mentioned in the text are indicated. **b**, Effects of LTL GWAS variants on LTL (*x* axis) and CH (*y* axis) outcomes. Variants are grouped into ‘cloud 1’ (shaded brown) and ‘cloud 2’ (shaded blue) according to their direction of effect on CH (see text). **c**, Effects of CH GWAS variants on CH (*x* axis) and MPN (*y* axis) outcomes. MPN outcomes were obtained from meta-analysis of ISL and UKB data (*n*_case_ = 1,124 and *n*_control_ = 747,154). In all panels, only variants with MAF > 1% are plotted. The plotted points are association effect estimates from logistic/linear regression and the bars indicate 95% CI. The red dotted lines indicate the IVW regressions. The chromosomal location of each plotted variant is indicated by color as indicated in the color key, lower right.

Several CH GWAS variants were reportedly associated with leukocyte telomere length (LTL) in the GWAS Catalog. To investigate this in detail, we examined the relationship between CH and LTL, using UKB samples that were contemporaneously assessed for both CH (in this study) and LTL (in ref. ^48^). CH, along with age and prior or current smoking, was strongly associated with shorter LTL (*β* = −0.129, *P* < 2 × 10^−16^; Supplementary Table 17) as seen previously in ISL^1^. Moreover, most CH GWAS variants associated with shorter telomeres, in line with the CH:LTL phenotype association. However, the two chr5:*TERT* variants and a variant on chr6p22 (near the MHC) were significantly associated with longer telomeres (Fig. 4a and Supplementary Table 18). As a result of this discordance, no significant regression parameters could be obtained and, consequently, a Mendelian randomization (MR) analysis was not considered prudent. For a complementary examination of the effects of LTL GWAS variants on the CH phenotype, we conducted a new GWAS for LTL in the UKB, using our current WGS-based imputation. We found 191 LTL variants (Supplementary Table 19). Their effects on LTL and CH are plotted in Fig. 4b. We found evidence of a massive discordance of effects, with some longer LTL alleles associated with increased CH risk and others associated with reduced risk (indicated as ‘cloud 1’ and ‘cloud 2,’ respectively, in Fig. 4b). Here again, MR analysis was not considered advisable.

**Extended Data Fig. 7:**
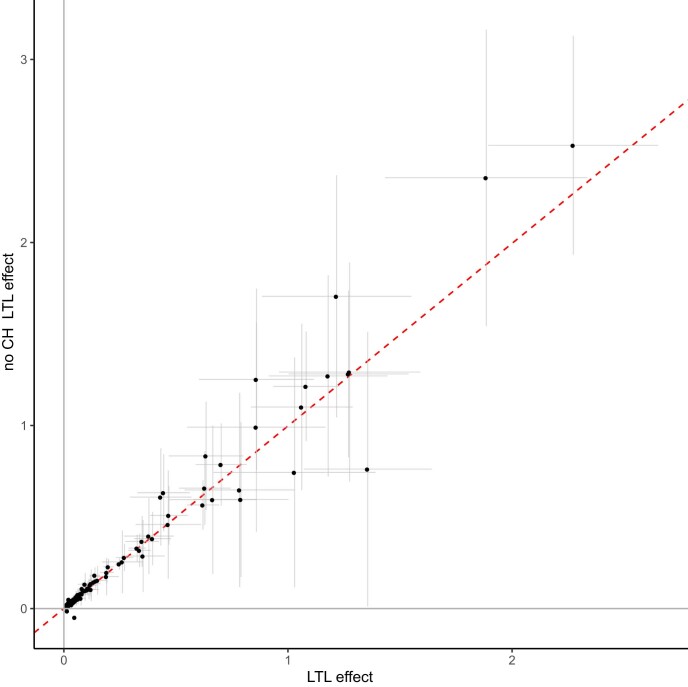
Effects leukocyte telomere length (LTL) GWAS variants on LTL in UKB and in a UKB sub-sample with barcode-CH cases removed. A GWAS was conducted on a sub-sample of UKB from which proven CH cases had been removed (n = 111,523). The effects of LTL GWAS variants were compared between the two samples: LTL effect on the x-axis and the no CH LTL effect on the y-axis. The plotted points are association the effect estimates from linear regression and the bars indicate 95%CI. The red dotted line indicates the fitted inverse variance weighted (IVW) regression. Gray lines indicate the position of no effect.

Observed LTL is measured in blood that may contain CH expansions. So, any variant that promotes CH but does not directly affect telomeres would appear to cause shorter telomeres, because of the association between CH and contemporaneously observed short telomeres. By the same token, such CH-promoting variants might be identified as LTL-associated variants in an LTL GWAS. To examine this, we repeated the GWAS for LTL, using only participants without proven CH. There was no evident difference in the effects of LTL GWAS variants between the two subgroups (Extended Data Fig. 7).

As was shown in Fig. 2a, CH associated strongly with subsequent diagnoses of MPN in line with its proposed status as a clinical precursor to MPN^49^. The majority of CH GWAS variants also conferred risk of MPN (Fig. 4c and Supplementary Table 18). MR analysis was consistent with CH having a causative effect on MPN (inverse-variance weighted (IVW), *P* = 7.86 × 10^−6^; Supplementary Table 20).

### CH GWAS variants are involved in expression quantitative trait loci (eQTL), splicing quantitative trait loci (sQTL) and protein quantitative trait loci (pQTL)

**Fig. 5:**
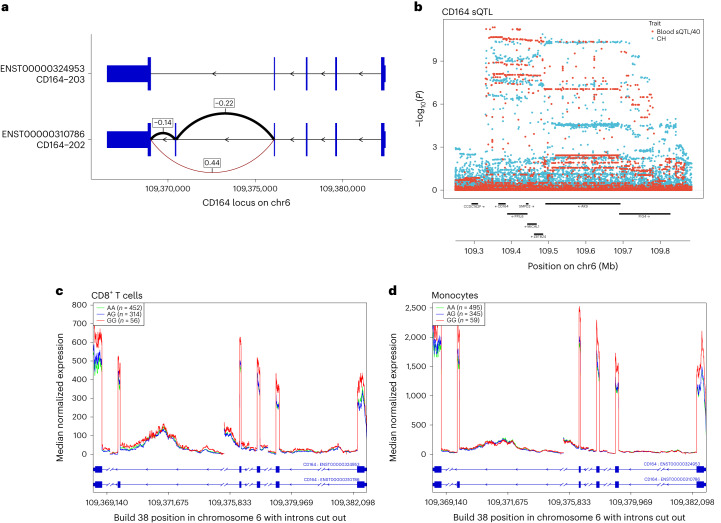
CH GWAS variants are associated with splicing and expression of CD164. **a**, Splice diagram of the two major *CD164* mRNA isoforms from whole blood RNA-seq data. Blue bars depict exons and are wider in coding regions. Introns are depicted as black arrowed lines. The sQTL affects skipping or inclusion of exon 5. Effects (*β* in s.d. units) from linear regression of the CH risk rs3056655_A allele are as follows: E4 to E6 (*β* = 0.44, *P* = 3.04 × 10^−302^; E4 to E5 (*β* = −0.22, *P* = 3.29 × 10^−72^); E5 to E6 (*β* = −0.14, *P* = 4.16 × 10^−32^). Thickness of the arcs indicates the overall usage of the different splice junctions. Black arcs indicate a reduction in usage in association with rs3056655_A, while the brown arc indicates an increase. **b**, Colocalization plot of the *CD164* locus showing association from logistic/linear regression of rs3056655 with CH (blue) and with the E4 to E6 splice event in whole blood (red, −log_10_(*P*) is divided by 40 for scaling). **c**, RNA-seq coverage plot of *CD164* from 822 CD8^+^ cytotoxic T cell samples, stratified by rs3056655 allele, showing reduced levels of expression in rs3056655_A (CH at-risk) heterozygotes and homozygotes. Note that rs3056655 is multi-allelic, but only the rs3056655_A (CH at-risk) and _G (CH protective) alleles were seen in the RNA-seq samples. **d**, As **c**, but RNA-seq from 899 monocyte samples.

**Extended Data Fig. 8:**
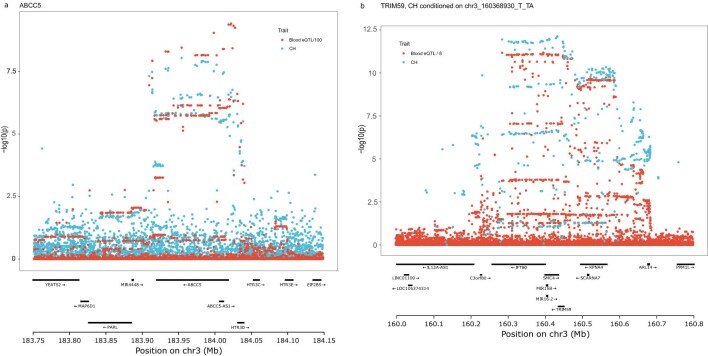
Co-localization of eQTL with CH GWAS loci chr3q27:ABCC5 and chr3q25:TRIM59/SMC4. **a**, Public databases report that *ABCC5* expression is down regulated in association with the CH risk allele chr3:183954156_GT in whole blood, monocytes and T-cells. This eQTL was confirmed in ISL whole blood RNAseq (β = −0.926 sd, *P* = 1 × 10^−1657^). We noted a closely correlated, moderate impact splice region variant (rs7636910, r^2^ = 0.96) in *ABCC5*. The panel shows a plot of RNAseq eQTL signals from whole blood (red) and CH GWAS results (blue) by genomic location. eQTL *P-*values are scaled as indicated in the legend. Co-localization analysis (COLOC^57^) indicated a PP.H4 = 74% probability that the eQTL and CH GWAS signals arise from the same, single causative variant. *ABCC5* is, however, not a compelling biological candidate for CH causation. **b**, Public databases report that *TRIM59* and *SMC4* expression in blood is increased in association with CH risk allele rs2305407_A, which is annotated as an *SMC4* splice region variant. These signals replicated in ISL blood RNAseq (*TRIM59*: β = 0.458sd, *P* = 1 × 10^−420^; *SMC4*: β = 0.073sd, *P* = 1.75 × 10^−11^). There were two independent CH GWAS signals at 3q25; a 1-2%EAF CH risk variant chr3_160368930_T_TA and a ∼ 55%EAF CH risk variant rs2305407_A, which carries the eQTL association. Accordingly, the CH GWAS plot (blue) shows the *P*_*adj*_ values for rs2305407_A conditioned on chr3_160368930_T_TA. The *TRIM59* RNAseq eQTL signal (red) is scaled as indicated in the legend. COLOC revealed a PP.H4 = 96% probability of peak identity. COLOC did not show substantial evidence of peak identity with the *SMC4* eQTL, whether the CH GWAS signal was conditioned on chr3_160368930_T_TA or not, with PP.H4 = 4.5% and 2.2%, respectively. eQTL and CH GWAS signals were derived from linear and logistic regression association analysis, respectively.

We considered whether the CH GWAS variants affect RNA abundance or splicing of nearby genes. For each sentinel variant, we identified all variants in LD (*r*^2^ ≥ 0.8) and then queried public RNA-seq eQTL and sQTL databases, focusing on blood or blood-related cell types. Variants with substantial *cis* effects were investigated further in ISL RNA-seq data from 17,848 peripheral blood samples (Supplementary Table 21). eQTL at *ABCC5* and *TRIM59/SMC4* are described in Extended Data Fig. 8, while other salient examples are discussed below:

CD164 is, biologically, a good candidate for a role in CH pathogenesis. It is expressed on early HSC and can affect their proliferation, differentiation, adhesion to bone marrow stromal elements, migration and retention in HSC niches^50–52^. Public sources revealed a *CD164* sQTL in blood, lymphoblastoid B-cell lines (LCL) and several nonhematological tissues. The top reported sQTL in whole blood has *r*^2^ = 0.81 with our sentinel CH GWAS hit (rs3056655), while the top sQTL in LCL has *r*^2^ = 0.86. Using ISL blood RNA-seq, we ascertained that the sQTL affects the two major isoforms of *CD164*, which differ by the presence (CD164-202) or absence (CD164-203) of exon 5. The latter isoform lacks the full-length CD164 protein’s glycosaminoglycan attachment site. Increased exon 5 skipping was strongly associated with the rs3056655_A CH risk allele (*P* = 3.04 × 10^−302^, *β* = 0.44). Coverage plots of ISL RNA-seq data from CD8^+^ T cells and monocytes revealed a decrease in overall *CD164* gene expression associated with the CH risk allele rs3056655_A (Fig. 5).

**Fig. 6:**
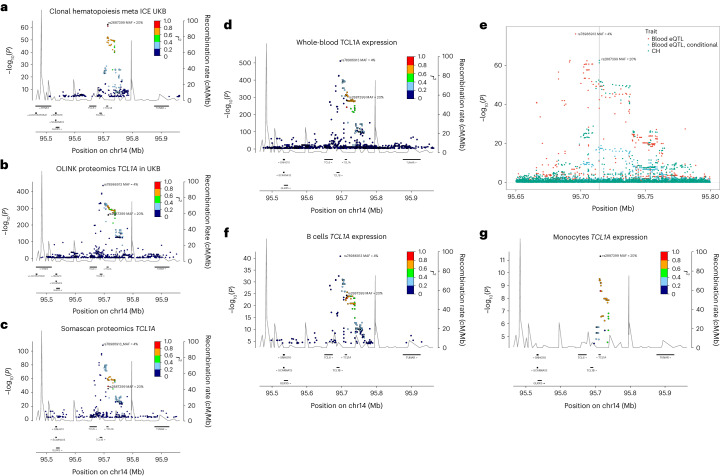
CH risk variants, pQTL and eQTL at the *TCL1A* locus. **a**, Locus zoom of CH GWAS results at *TCL1A*. **b**, *Cis*-pQTL analysis of variants affecting plasma protein levels of TCL1A in 47,133 UKB participants. **c**, As **b**, but from 35,559 ISL participants. **d**, RNA-seq *cis*-eQTL analysis of *TCL1A* in whole blood. **e**, Colocalization analysis of CH GWAS and blood eQTL signals at the *TCL1A* locus. The CH GWAS (green) and unadjusted eQTL signals (red) do not coincide. However, when the eQTL signal is adjusted for the 4% MAF rs78986913 variant (*P*_adj_ values shown in blue), then the peaks overlap with a PP.H4 = 85% probability that they correspond to the same signal. The position of the CH GWAS sentinel variant rs2887399 is indicated by the gray vertical line. **f,** *TCL1A* eQTL from 758 B cell RNA samples. **g**, *TCL1A* eQTL from 884 monocyte samples. In all panels except **e**, the *r*^2^ focus is on rs2887399.

We carried out a proteomic analysis of plasma samples from 12,636 UKB participants for whom we had CH status information, using the Olink platform to interrogate levels of 1,472 proteins and test them for association with CH. Several proteins of relevant biological interest ranked highly (by significance), including the hematopoietic progenitor cell growth factors FLT3LG and CLEC11A, thrombopoietin THPO, pro-inflammatory cytokines CCL5 and TNFSF12 and smoking marker ALPP (Supplementary Table 22). Second in the ranking was TCL1A, an oncoprotein in T cell leukemias, lymphomas, CLL and several nonhematological cancers^53^. Higher TCL1A levels were associated with CH (*P* = 2.05 × 10^−13^, *β* = 0.21), and this replicated ISL SomaScan proteomic data (*P* = 2.86 × 10^−3^, *β* = 0.06) (ref. ^54^). *TCL1A* is of particular interest because a CH GWAS variant is located 162 bp upstream of the gene’s transcription start site (Fig. 6a). The minor allele, rs2887399_T (minor allele frequency (MAF) ∼20%), is protective against CH in our data. It has been implicated (with varying direction of effect) in CPLD-CH, mCA and LoY (see above and refs. ^13,41,55^). The rs2887399_T allele is reported to suppress ectopic expression of *TCL1A* in CPLD mutant HSC^56^. A search for *cis*-pQTL using UKB Olink and ISL SomaScan identified two conditionally independent LD classes of variant, both with minor alleles acting to reduce *TCL1A* expression. One LD class of pQTL was correlated with rs2887399_T (*r*^2^ ∼0.67), whereas a second LD class pQTL, typified by rs78986913_A was not (*r*^2^ ∼0.092, MAF ∼4%; Fig. 6b,c). Curiously, rs78986913_A did not show an independent signal in GWAS for CH predisposition in conditional analysis (*P*_adj_ = 0.78).

To investigate this further, we searched for RNA-seq *cis*-eQTL for *TCL1A*. In whole blood, both the 4% MAF rs78986913_A and the 20% MAF rs2887399_T variant classes reduced expression of *TCL1A*. Conditioning the eQTL signal on rs78986913, COLOC^57^ revealed an 85% probability of peak identity between the rs2887399 eQTL and the CH GWAS peak. Both the 4% MAF and 20% MAF variants classes affected expression in B cells. However, in monocytes only the 20% MAF rs2887399_T variant was associated with *TCL1A* RNA expression and a 4% MAF rs78986913_A peak was not in evidence (Fig. 6d–g). It appears that, in this case, the eQTL and pQTL of relevance to CH may be restricted to the myeloid lineage.

## Discussion

This study expands greatly on our previous investigation of CH detected using mutational barcodes^1^, extending the number of cases from 1,403 to 16,306. We reaffirm the strong associations between CH, age and smoking and provide evidence that smoking has a dose-dependent impact on CH. Aside from confirming the risk for hematological diseases, we find that CH associates with COPD, lung cancer, PAD, emphysema and alcohol abuse. These conditions are all smoking-related. The effects of CH on their risks were strongly attenuated when adjusted for smoking. It is likely that the remaining associations are due to residual confounding from various aspects of smoking behavior that could not be fully taken into account in the analysis. It is notoriously difficult to remove all residual confounding from smoking behavior, especially when using self-reported information^58,59^. An attractive hypothesis is that smoking creates an inflammatory state, exerting pressure on the hematopoietic system, depleting the HSC and progenitor cell pool and driving compensatory HSC self-renewal, thereby increasing the probability of a clonal outgrowth^60–62^.

Studies that reported an association between CH and CVD received a great deal of attention, having been reviewed extensively^15,21,22^. Somewhat less attention was given to contemporaneous studies reporting a lack of association, albeit sometimes in smaller samples^7–9,12,14,15,63^. The present study finds no evidence of an association between CVD and barcode-CH or CPLD-CH. The strong potential for confounding by age and smoking has been emphasized, here and elsewhere^14^. Moreover, our stringent exclusion of people with a pre-existing hematological abnormality may be a factor. Some hematological disorders (particularly MPN) have known associations with blood clotting and CVD risk^64^. We observed an increased incidence of CVD among the participants whom we excluded compared to participants without CH (HR = 5.08, *P* < 2 × 10^−16^). We also note that published CVD risks are seen particularly for *ASXL1*-CH (which has a demonstrable smoking bias) and *JAK2*-CH (which associates strongly with MPN)^9,15^. Not taking these considerations sufficiently into account may create or inflate an apparent CVD risk.

There may be a large number of undiscovered mutations that confer a sufficient fitness advantage to drive HSC clonal expansions to overt CH over a long period of time^2,24,25^. We find several genes that are not well recognized as CH drivers, some with previously noted involvement in myeloid (or in some cases lymphoid) disease. Nevertheless, most CH still cannot be accounted for by an obvious driver mutation. No satisfactory explanation has yet emerged and the question merits further investigation.

Here we provide new evidence for 25 loci with germline variants that predispose to barcode-CH. We additionally identify three secondary signals and two suggestive, missense variants. Several variants overlap with loci that have been associated with CPLD-CH, mCA, LoY and MPN, underlining the close relationships between these phenotypes^1,9,14,15,42–44,65,66^. CH GWAS variants commonly show pleiotropic associations with blood cell traits, LTL and MPN but not CVD—no CH GWAS variants had listings for CVD in the GWAS Catalog, and MR analysis gave no indication that CH risk variants increased CVD outcomes (Supplementary Table 20).

Based on MR using the few instrumental variables that were available to them at the time, a study described in ref. ^67^ concluded that long-LTL alleles predispose to CH, whereas CH alleles predispose toward shorter telomeres. This is not fully consistent with our observations, in which we see many discordant effects (Fig. 4). MR studies typically show that long-LTL alleles are associated with cancer predisposition, whereas observed telomere lengths in blood of predisposed people or in tumors can be either longer or shorter. Indeed, we find that CH is linked to shorter observed LTL, perhaps as a result of extra divisions that an HSC clone had to undertake to gain its dominance (see Fig. 4a above and ref. ^1^). In leukemias, paradoxically, risk is increased by both long and short observed LTL, measured prospectively^68^. A rationalization for this, as evidenced in congenital telomeropathies, could be that too short telomeres impair HSC function and precipitate a bone marrow insufficiency. This places a selective pressure on the HSC population and the marrow is repopulated by HSCs that have acquired alterations allowing them to bypass the replicative exhaustion induced by the telomere erosion^69,70^. MR studies in MPN, CLL and leukemias in general implicate long-LTL alleles as risk factors^44,68,71,72^. A long-LTL genetic constitution may relax the replicative constraints that normally keep HSC expansions in check, allowing emergent HSC clones to expand and present a larger target for secondary oncogenic events. It is therefore plausible that both long-LTL and short-LTL variants could act to promote CH.

## Methods

### Epidemiology

#### Iceland

The study included WGS of whole blood samples from 45,699 Icelanders participating in various projects at deCODE genetics. The study was authorized by the Icelandic National Bioethics Committee and the Data Protection Authority (License VSN-16-104). All individuals gave written informed consent.

#### UKB

The study included WGS of whole blood samples from 130,709 participants in the UKB. The study was authorized by the North West Research Ethics Committee (reference 06/MRE08/65). All individuals gave written informed consent. Genotype and phenotype data for our study were obtained, and research was conducted under the UKB application license 56270.

#### Study exclusion criteria

For UKB we included only participants with self-declared British or Irish ethnicity (UKB field 21000). For ISL, to suppress germline singleton mutations in the samples (which can be mistaken for high VAF somatic mutations^1^), we excluded individuals with unproven Icelandic ancestry as far back as great-grandparents. Our definition of CH excludes pathological expansions of defined, committed lineages such as lymphomas, leukemias, MDS and MPN. Accordingly, participants were excluded from most analyses (unless otherwise specified) if they had a diagnosis of a hematological disorder (International Classification of Diseases, Tenth Revision (ICD10) codes C81-C96 and D45-D47) before or within 6 months after blood draw. Participants were also excluded if they had substantial evidence of abnormality from hematology parameters measured at recruitment (if available), comprising white blood cells (WBC) < 1.5 × 10^9^ or >35 × 10^9^ cells per l or hemoglobin concentration (HGB) < 8 g dl^−1^, or platelet count (PLT) < 50 × 10^9^ cells per l.

### WGS for CH case definition

#### UKB

Reads were aligned to GRCh38 reference (GRCh38 reference with alt contigs plus additional decoy contigs and HLA genes) with bwa mem (v0.7.17). Duplicates were marked using Picard MarkDuplicates (v2.20.3). A base quality recalibration table was created using GATK BaseRecalibrator (v4.0.12) with known sites files dbSNP138, Mills and 1000G gold standard indels, and known indels from GATK resource bundle (from gs://genomics-public-data/resources/broad/hg38/v0). For each chromosome in chr1 to chr22, chrX, chrY, the resulting base recalibration table was applied using GATK ApplyBQSR (v4.0.12) and then variants were called for each sample individually using GATK HaplotypeCaller and GATK GenotypeGVCFs (v4.0.12). Variants were (hard) filtered using criteria in http://gatkforums.broadinstitute.org/discussion/2806/howto-apply-hard-filters-to-a-call-set. Average sequence depth was 33.

We extracted all singleton SNPs (SNPs occurring only once in the UKB cohort) for 149,960 participants, then filtered on genotype quality (GQ) ≥ 90 to obtain some 287 million singleton variants (ignoring hard filtering).

The following filter steps were applied:

- use FILTER in (PASS, Low_QD)
- 15 ≤ depth ≤ 60
- minor allele reads ≥3 to remove spurious low-VAF bump

We estimate the number of somatic singleton mutations with 0.1 ≤ VAF ≤ 0.25 as the number of observed variants in this VAF range minus the number of expected germline variants. To model the expected number of germline variants in this VAF range, we make the following assumptions:

- The expected number of germline variants in the VAF ranges 0.1–0.25 and 0.75–0.9 are approximately equal (that is, there is symmetry in the germline variant VAF distribution).
- The vast majority of variants in VAF ranges 0.35–0.65 and 0.75–0.90 are germline variants.
- The ratio of germline variants in VAF ranges 0.75–0.90 and 0.35–0.65 is approximately constant for each participant, given sequencing depth and sequencing center.

For each depth, we compute the ratio of total observed (germline) variants in VAF range 0.75–0.9 compared to VAF range 0.35–0.65. This computation is done separately for each sequencing center. For each participant, the number of expected germline variants in VAF range 0.1–0.25 for a given sequencing depth is then computed as the expected fraction of germline variants in VAF range 0.75–0.9, given the observed number of variants in VAF range 0.35–0.65 at the given depth. Only sequencing depths ≥21 were considered. Based on an expected fraction of CH of around 1% at age 40, we set a threshold of ≥20 observed somatic singleton indicator mutations with 0.1 ≤ VAF ≤ 0.25 to define CH. This threshold was adjusted for sequencing center (+1 for Vanguard and −2.2 for Sanger) to achieve agreement of age dependency between the sequencing centers. Note that the VAF of the indicator mutations is not a precise measurement of the VAF of the CH clone—because only ∼20 indicator mutations are required to define CH, VAF distributions of somewhat smaller and larger clones are likely to pass through the detection window. Moreover, larger clones will generate subclones with indicator mutations of lower VAF.

#### ISL

For ISL, we needed to accommodate for different sequencing platforms. A total of 33,189 samples sequenced on Illumina HiSeqX were processed to determine CH status as previously^1^. For 12,510 samples sequenced on Illumina NovaSeq, reads were aligned to hg38 reference using bwa mem (v0.7.10), indels realigned using GATK IndelRealigner (GATK 2.3-9) and duplicates removed using Picard MarkDuplicates (V1.117). Genotypes were called using GATK HaplotypeCaller and GATK GenotypeGVCFs (v.2014.4-3.3.0-0-ga3711aa). Variants were (hard) filtered as above. CH status was determined as described above for UKB; however, singletons were determined based on a cohort of ∼100,000 sequenced participants. As no base quality recalibration was applied to ISL, the estimated number of somatic singletons for 0.1 ≤ VAF ≤ 0.25 was higher than for UKB (46 for WGS NoPCR Nova and 32 for NEB WGS). Average sequence depth was 38.

#### Definition of CPLD-CH

We ran Strelka2 (2.9.10) somatic workflow on CPLD gene regions on CRAM files from genome alignment (see above). To suppress artifacts due to mapping problems, we used one of the CRAM files as a normal sample for all other samples. Variants were filtered on depth >10, FILTER = ‘PASS,’ and 0.01 ≤ VAF ≤ 0.99. To identify germline variants, we performed a binomial test on VAF against 0.5, and classified calls with *P* > 0.05 as potential germline calls. Variants with >5 observations and >75% potential germline calls were removed. We annotated the remaining variants using VEP and kept only those moderate/high-impact variants that were either high impact (but not in ‘GNAS,’ ‘JAK2,’ ‘SRSF2,’ ‘SF3B1’) or present in ref. ^13^.

Note that the definition of CPLD-CH is not subject to the same VAF restrictions as the mutational barcode method described above. Moreover, particularly in younger individuals, CPLD-CH can be detected in the absence of a mutational barcode, as discussed in ref. ^1^ (see also Supplementary Table 8).

To define CHIP in Table 2, we used the strategy recommended in refs. ^19,20^, adapted to our dataset. Variants in the 73 candidate genes (except U2AF1) were called using Strelka2. Variants were annotated with VEP v.100. Variants given in Vlasschaert Supplementary Table 1 (ref. ^20^) were selected and kept if they had depth ≥20 and minAD ≥3. Variants occurring at ≥15 times were tested for association with age and rs7705526—variants with *P* > 0.1 or estimate <0 for both covariates were removed. A binomial test was used to remove putative germline variants by testing if the read depth was statistically different from half of the sum of all sequencing reads at that site. Variants with *P* > 0.01 were removed, except for variant sites TET2 H1904R, I1873T and T1884A.

### Phenotypes and metadata

ISL phenotypic data were taken from national registries, hospital and project-based datasets curated in the deCODE genetics phenotypic database. For UKB, age at blood sampling was computed from UKB field 3166. ICD10 diagnoses were taken from the following UKB fields:

- ICD10 hospital inpatient summary (41270).
- Self-reported illnesses, cancer (20001) and noncancer (20002).
- Cause of death from death registry, primary (40001) and secondary (40002).
- Cancer registry ICD10 (40006) and ICD9 (40013).
- OPCS4 hospital inpatient summary (41272).

#### Smoking

The smoking phenotype was focused on heavy smokers (current or previous) and was defined based on the following UKB fields:

- 1249, past tobacco smoking (−3 = no answer, 1 = on most or all days, 2 = occasionally, 3 = tried once or twice, 4 = no) .
- 1239, current tobacco smoking (−3 = no answer, 1 = on most or all days, 2 = occasionally, 0 = no).
- 3436, age started smoking in current smokers.
- 2867, age started smoking in former smokers.
- 2897, age stopped smoking.
- 2887, number of cigarettes previously smoked daily.
- 3456, number of cigarettes currently smoked daily.

We used data only from the first assessment. Smoking status was defined as current if 1239 = 1 and previous if 1249 = 1, otherwise never. Pack years and years since stopped smoking were derived from 3436, 2867, 2897, 2887 and 3456. The fraction of participants with CH was modeled using logistic regression for all participants with the available covariates smoking status, pack years and years since stopped smoking. Nonlinear transformations for pack years and years since stopped smoking were based on the results of the generalized additive model.

Hematological disorders were classified according to the following scheme:

- All hematological disorders—C81-C96, D45-D47, then…
- Any myeloid disease—C92-C94, D45, D46, D47.0, D47.1, D47.3, D47.4, D47.5
- AML and related—C92.0, C92.4, C92.5, C92.6, C92.8, C93.0, C94
- CML and related—C92.1, C92.2, C92.3, C93.1, C93.3
- MDS—D46
- MPN (non-CML)—D45, D47.0, D47.1, D47.3, D47.4, D47.5
- PCV—D45
- ET—D47.3
- OMF—D47.4
- Any lymphoid disease—C81-C91, D47.2, D47.9
- CLL—C91.1
- MM—C90
- MGUS—D47.2

#### Disease and clinical hematology parameter phenotype–phenotype association testing

We tested for association between CH and case–control phenotypes by logistic regression, using sex and age at blood draw (linear and quadratic) as covariates. To correct for the influence of smoking, we also performed logistic regression using smoking status (and, in some cases, pack years and years since stopped smoking) as additional covariates. We estimated, conservatively, that we tested 10,000 independent disease phenotypes and set the Bonferroni adjustment level accordingly at 5.00 × 10^−6^. For clinical hematology parameters and other quantitative phenotypes, we tested for association between the number of somatic singletons and quantitative phenotypes by linear regression, using sex and age at blood draw (linear and quadratic) as covariates. The number of somatic singletons was inverse normal transformed stratified by sequencing center and sex. Quantitative traits were inverse normal transformed stratified by sex. To correct for the influence of smoking, we also performed linear regression using smoking status as an additional covariate.

#### Time-to-event analysis of CVD, hematological disorders, survival and cause of death analysis

For UKB, the median age at blood draw was 58.4 years and the median follow-up time was 12.0 years (range: 10.2–14.7). For ISL, the median age at blood draw was 53.0 years and the median follow-up time was 14.7 years (range: 0–20.8). For time-to-event and survival analysis, we fitted Cox proportional hazards models using the R package Survival (v3.3-1). We stratified by sex and 5-year age bin and adjusted for age at blood draw and smoking. Assumptions for the Cox proportional hazards model were checked using the ‘cox.zph’ function of the R package. The CAD phenotype comprised ICD10 codes from first reported diagnoses or cause of death (I200, I21, I210, I211, I212, I213, I214, I219, I21X, I22, I220, I221, I228, I229, I24, I240, I241, I248, I249, I25, I250, I251, I252, I256, I258, I259) and OPCS4 codes (K401, K402, K403, K404, K411, K412, K413, K414, K451, K452, K453, K454, K455, K491, K492, K498, K499, K502, K751, K752, K753, K754, K758, K759). Primary cause of death data were obtained from field 40001 for UKB and from the National Register of Deaths for ISL. Analysis was conducted where ≥10 participants had the same cause of death. Participants with nonqualifying causes of death were right-censored. For the time-to-event analysis of hematological disorders shown in Fig. 2, hematological events with ICD codes described above were registered if they occurred 6 months or more after sampling for CH assessment. Participants who could not be assessed for CPLD status were excluded. In an analysis of HR for CPLDneg-CH, participants who were barcode-CH positive, CPLD-CH positive were excluded.

### Somatic genetics

#### Gene-based somatic mutation burden testing

Burden testing of somatic variants was performed using SKAT-O^73^. For all protein-coding genes, we retrieved genotypes for those high/moderate-impact variants that occurred less than 500 (UKB) or 175 (ISL) times and removed likely germline variants (that occurred >5 times with a mean VAF between 0.45 and 0.55). SKAT-O was run with adjustment for age at blood draw, ethnicity, sex and sequencing center. We report on genes where one of the VEP categories was Bonferroni significant (*P* < 1.0 × 10^−6^) in one cohort and at least nominally significant in the other, or the *P*_combined_<1.0 × 10^−9^. Individual variants were assessed using Fisher’s exact test.

#### Chemotherapy and CPLD mutations

We extracted the date of first chemotherapy (OPCS4 code X72%, X73%) from the UKB phenotype database. In total, 403 participants had undergone chemotherapy before blood sampling. We then estimated the relative risk of a defined CPLD mutation by multivariable logistic regression including terms for age, sex and smoking status.

### CH GWAS

#### Genotyping, WGS and imputation

For ISL, 174,987 samples were genotyped using chip arrays from the Illumina OmniExpress family (*n* = 136,215) with the remaining samples using older HumanHap family chips. Sequence variants for imputation were identified by WGS data from 63,118 samples. Joint variant calling used GraphTyper v.1.4 (ref. ^74^). Genotypes for these variants were imputed into the chip-typed samples using long-range phasing^75^ yielding phased genotypes for 173,025 participants.

For UKB, chip genotyping, WGS and imputation are detailed in ref. ^17^. Briefly, genotyping was performed using a custom-made Affymetrix chip (UK BiLEVE Axiom) on the first 50,000 participants and the UKB Axiom for the remainder. Sequence variants for imputation were identified by WGS of 150,119 samples, performed by deCODE genetics and the Wellcome Trust Sanger Institute. Joint variant calling was performed using GraphTyper v.1.4. Long-range phasing was used to impute the WGS-derived genotypes into 431,079 participants.

#### CH GWAS, association testing and meta-analysis

Methods for GWAS association testing are described in detail elsewhere^17,76^. Briefly, association between imputed variants and barcode-CH as a binary phenotype was tested by logistic regression under a multiplicative genetic model. For ISL, the model included as covariates—sex, county of birth, current age or age at death (first- and second-order terms included) and an indicator function for the overlap of the lifetime of the individual with the time span of phenotype collection. In UKB, 20 principle components were used to adjust for population stratification, with age and sex included as covariates. LD regression was used to account for cryptic relatedness and stratification^77^. Analysis of quantitative hematological parameters and LTL used the linear mixed model implemented in BOLT-LMM^78^. For meta-analyses, GWAS results from ISL and UKB were combined using a fixed-effects inverse-variance method based on effect estimates and s.e. in which each dataset was assumed to have a common OR but allowed to have different population frequencies for alleles and genotypes. Sequence variants were mapped to NCBI Build 38 and matched on position and allele to harmonize the datasets. We tested ∼75.2 million variants for association, with MAF > 0.001% and imputation information >0.8 in at least one of the cohorts. For conditional analysis, the sentinel signal at each locus was defined as the variant with the lowest Bonferroni adjusted *P* value using adjusted significance thresholds^79^. Conditional analysis used individual-level genotype data to test possible secondary signals ±500 kb from the sentinel signal.

#### CPLD-CH GWAS

The GWAS was repeated using individuals who were identified as carrying a somatic mutation in CPLD genes as affected. For the CPLD-CH × barcode-CH effect × effect plots, variants were included if they were associated at *P* < 5 × 10^−8^ (or 5 × 10^−7^ for moderate- or high-impact variants) in barcode-CH or in any one of the CPLD-CH classes and had not been excluded as high impact, rare variants as indicated in Supplementary Table 9. Variants were not plotted if they had abs(log_e_OR) > 3, but they were included in the data table (Supplementary Table 12).

#### Investigation of pleiotropic traits in the GWAS Catalog

For each sentinel variant, we identified all variants in LD (*r*^2^ ≥ 0.8) within ±500 kb. For those variants, we then searched the GWAS Catalog^80^ for reported associations with *P* < 1 × 10^−7^.

#### LTL and MPN effect × effect plots and MR

Variants selected for effect × effect plots and MR of LTL and MPN were genome-wide significant according to stringent weighted Bonferroni criteria after stepwise conditional analysis at each locus^79^. LTL variants and effects were determined by GWAS using UKB LTL data^48^. MPN outcomes were freshly recalculated using current UKB data (Supplementary Table 18). MR analyses were performed using linear regression without an intercept term, weighted by the inverse-variance of the outcome associations (IVW), MR coupled with an intercept test and weighted linear regression with an intercept term (MR-Egger^81^).

#### RNA eQTL and sQTL analysis

Public domain databases that were screened for RNA-seq eQTL and sQTL data are detailed in the Data Availability section. In-house RNA-seq analysis was performed as an extension of our previous studies^76,82^—we isolated RNA from whole blood samples from ISL participants (*n* = 17,848), in addition to 822 T cell, 758 B cell and 899 monocyte samples, using Chemagic Total RNA Kit special (PerkinElmer) and sequenced it using Illumina HiSeq 2599 and NovaSeq systems. STAR software (v.2.5.3) was used to align RNA-seq reads to personalized genomes^83^. Kallisto^84^ was used to estimate transcript abundances. BOLT-LMM was used to test additive model association between transcript abundance and genetic variants. Adjustment factors were as follows: sequence artifact estimations, demographic characteristics, blood cell counts and 100 leave-one-chromosome-out (LOCO) principle components of the gene expression matrix. The top *cis*-eQTL was defined as the variant with the most significant association within 1 Mb of the gene.

LeafCutter (v.0.2.6) (ref. ^85^) was used to quantify RNA alternative splicing. Linear regression under the additive model was used to test the association between alternative splicing events and linked genetic variants using quantile-normalized-percentage-spliced-in (PSI) values for each junction. Adjustment factors were as follows: sequence artifact estimations, demographic characteristics, blood cell counts and 15 LOCO principle components of the quantile-normalized PSI matrix. Colocalization analysis between CH GWAS variants and eQTL was carried out using COLOC^57^ implemented in R.

#### Proteomics

Proteomic analysis of ISL plasma samples (including *n* = 18,527 participants assessed for CH) using the SomaScan version 4 panel was described previously^54^. Proteomic analysis of UKB plasma samples (*n* = 12,636 participants with CH assessment) was conducted using the Olink Explore 1536 platform as part of the UKB-Pharma Proteomics Project (UKB application 65851). The vast majority of the samples were randomly selected from among UKB participants. Olink measurements used the normalized protein expression (NPX) values recommended by the manufacturer, which include normalization.

To test for associations between plasma protein levels and CH, we used the following model: protein level ∼ CH + age + sex + smoking + blood count phenotypes, where the smoking phenotype is ‘ever smoked’ (UKB ID20160) and blood count phenotypes are WBC, eosinophil (EO) %, lymphocyte number (LY#), plateletcrit (PCT), platelet (PLT), high light scatter reticulocyte number (HLR#), HLR%, monocyte number (MO#), reticulocyte (RET) %, immature reticulocyte fraction (IRF), reticulocyte number (RET#) platelet distribution width (PDW), mean corpuscular hemoglobin (MCH), mean corpuscular volume (MCV), mean sphered cell volume (MSCV), basophil (BA) %, MO%, eosinophil number (EO#), neutrophil (NE) %, and red cell distribution width (RWD). All the blood-related phenotypes were corrected for age and sex and standardized to normal distribution before entering into the model.

#### Statistical testing

All statistical tests used in the study were two-sided. None of the *P* values quoted were adjusted for multiple testing.

### Reporting summary

Further information on research design is available in the Nature Portfolio Reporting Summary linked to this article.

## Online content

Any methods, additional references, Nature Portfolio reporting summaries, source data, extended data, supplementary information, acknowledgements, peer review information; details of author contributions and competing interests; and statements of data and code availability are available at 10.1038/s41588-023-01555-z.

### Supplementary information

Supplementary Information Supplementary Fig. 1.

Reporting Summary

Supplementary Tables Supplementary Tables 1–22.

## Data Availability

In addition to data presented in Supplementary Tables 1–22, the following new datasets are made available at: https://www.decode.com/summarydata/ 1. Variant level GWAS meta-analysis data for ISL and UKB for barcode-CH and each CPLD-CH type illustrated in Fig. 3. 2. Mutation level counts and Fisher’s exact test results for each somatic mutation tested in ISL and UKB. WGS, genotype and phenotypic data for UKB participants can be accessed by approved researchers via the UKB research analysis platform: https://ukbiobank.dnanexus.com/landing. Guidance on access can be found here: apply for access (ukbiobank.ac.uk). Individual-level ISL WGS, RNA-seq and phenotype data cannot be made publicly available because that is prohibited by the Icelandic Act on Data Protection and Processing of Personal Data and conditions set forth to us by the Icelandic Data Protection Authority. On-site access to the data at deCODE genetics facilities may be granted. Interested parties should write to the lead contact author S.N.S. with a brief description of the requirements and intended use. Requests will be discussed by the deCODE data access committee and a response given within 4 weeks. We used data from the following public domain sources: GWAS Catalog^80^ (https://www.ebi.ac.uk/gwas/home 26/10/2021 release) for reported GWAS associations. GTEx v8 (ref. ^86^; https://gtexportal.org/home/) for eQTL/sQTL, various tissues. eQTL Catalog^87^ (https://www.ebi.ac.uk/eqtl) for eQTL/sQTL, various tissues. GEUVADIS^88^ (https://www.cnag.crg.eu/projects/geuvadis) for eQTL/sQTL in LCL. Ref. ^89^ for eQTL/sQTL in monocytes, neutrophils and T cells. eQTLGen Consortium^90^ (https://www.eqtlgen.org) for eQTL/sQTL in blood. Ref. ^91^ for eQTL/sQTL in vascular and metabolic tissues. xQTL Serve^92^ (https://mostafavilab.stat.ubc.ca/xQTLServe) for eQTL/sQTL in brain. Ref. ^93^ for eQTL/sQTL in dendritic cells. Ref. ^94^ for eQTL/sQTL in monocytes. MuTHER^95^ (http://www.muther.ac.uk) for eQTL/sQTL in adipose, LCL and skin. Ref. ^96^ for eQTL/sQTL in liver. Ref. ^97^ for eQTL/sQTL in lung. Ref. ^98^ (https://nephqtl.org) for eQTL/sQTL in kidney. Ref. ^99^ (http://icahn.mssm.edu/gwas2genes) for eQTL/sQTL in various tissues. Ref. ^100^ for eQTL/sQTL in leukocytes. Ref. ^101^ for eQTL/sQTL in blood. Ref. ^102^ (GEO (https://www.ncbi.nlm.nih.gov/geo) accession GSE196830) for eQTL/sQTL in 14 immune cell types. Ref. ^103^ for eQTL/sQTL in LCL.

## Code availability

No custom code/software was used in the study. The publicly available software used is indicated in the Methods above and collated in the attached Reporting Summary.
