# Supplementary material for: Genetics and epidemiology of mutational barcode-defined clonal hematopoiesis
Source: Nat Genet. 2023 Nov 6;55(12):2149–59. doi: 10.1038/s41588-023-01555-z (PMC10703693; doi:10.1038/s41588-023-01555-z)
Supplement: Supplementary file 1 — Supplementary Fig. 1. [file 41588_2023_1555_MOESM1_ESM.pdf]

nature genetics

Article

https://doi.org/10.1038/s41588-023-01555-z

Genetics and epidemiology of mutational barcode-defined clonal hematopoiesis

In the format provided by the authors and unedited

Panel A

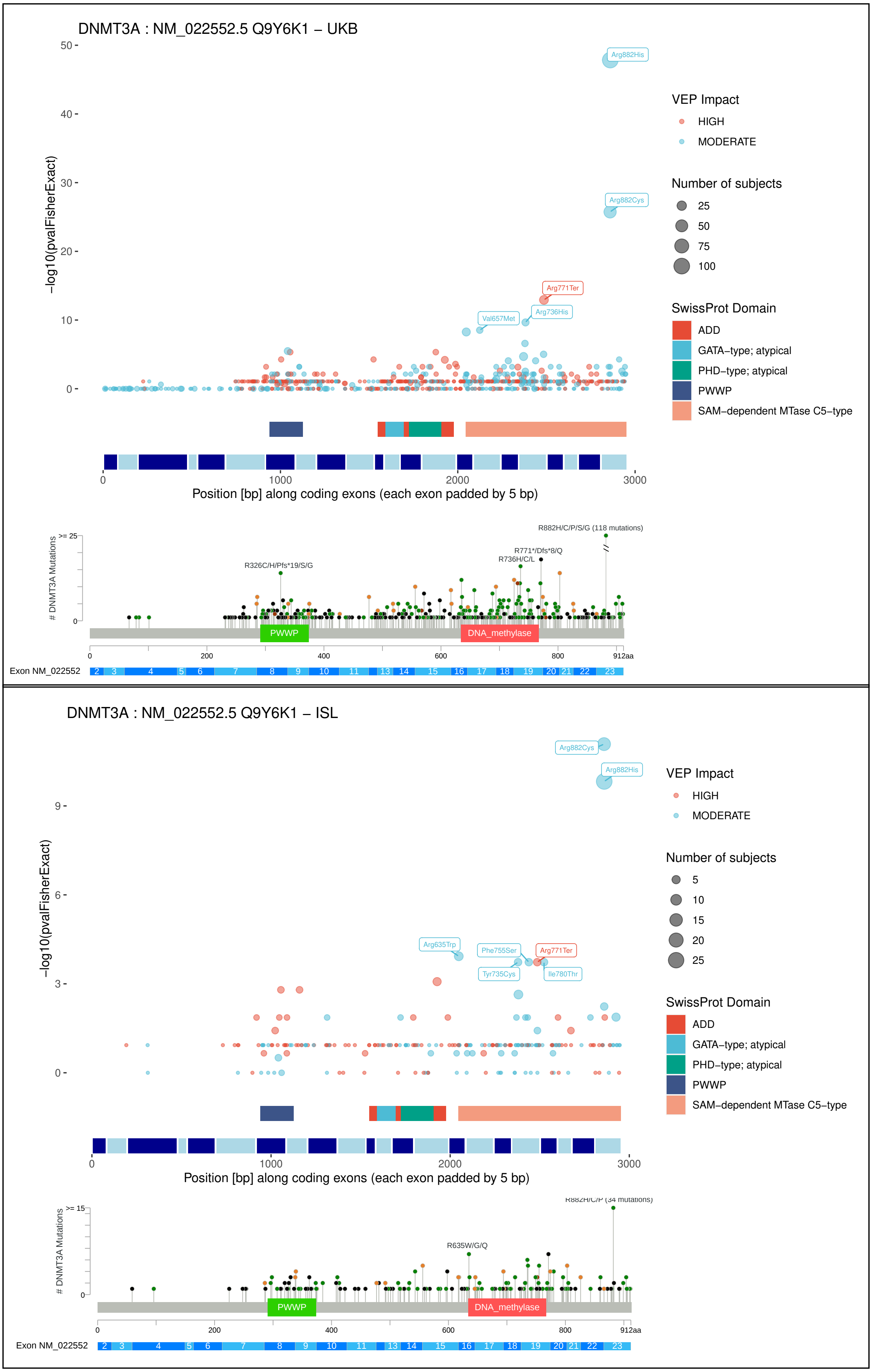
DNMT3A : NM_022552.5 Q9Y6K1 – UKB
−log10(pvalFisherExact)
50
40
30
20
10
0
Arg882His
Arg882Cys
Arg771Ter
Arg736His
Val657Met
VEP Impact
HIGH
MODERATE
Number of subjects
25
50
75
100
SwissProt Domain
ADD
GATA−type; atypical
PHD−type; atypical
PWWP
SAM−dependent MTase C5−type
0
1000
2000
3000
Position [bp] along coding exons (each exon padded by 5 bp)
DNMT3A Mutations
>= 25
0
R882H/C/P/S/G (118 mutations)
R771*/Dfs*8/Q
R736H/C/L
R326C/H/Pfs*19/S/G
PWWP
DNA_methylase
0
200
400
600
800
912aa
Exon NM_022552
2 3 4 5 6 7 8 9 10 11 13 14 15 16 17 18 19 20 21 22 23
DNMT3A : NM_022552.5 Q9Y6K1 – ISL
−log10(pvalFisherExact)
9
6
3
0
Arg882Cys
Arg882His
Arg635Trp
Phe755Ser
Arg771Ter
Tyr735Cys
Ile780Thr
VEP Impact
HIGH
MODERATE
Number of subjects
5
10
15
20
25
SwissProt Domain
ADD
GATA−type; atypical
PHD−type; atypical
PWWP
SAM−dependent MTase C5−type
0
1000
2000
3000
Position [bp] along coding exons (each exon padded by 5 bp)
DNMT3A Mutations
>= 15
0
R882H/C/P (34 mutations)
R635W/G/Q
PWWP
DNA_methylase
0
200
400
600
800
912aa
Exon NM_022552
2 3 4 5 6 7 8 9 10 11 13 14 15 16 17 18 19 20 21 22 23

Panel B

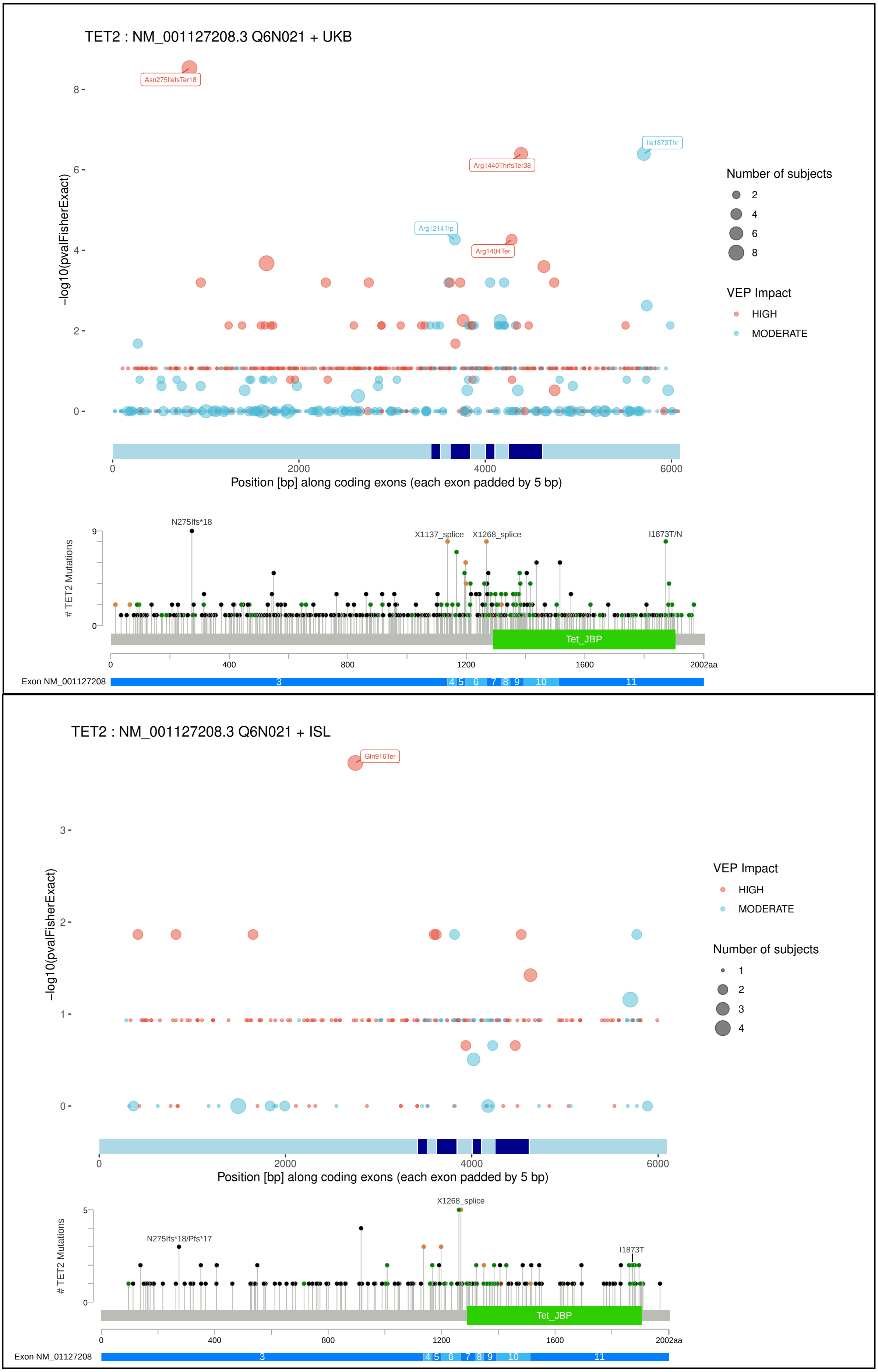

TET2 : NM_001127208.3 Q6N021 + UKB
Asn275IlefsTer18
Arg1440ThrfsTer38
Ile1873Thr
Arg1214Trp
Arg1404Ter
−log10(pvalFisherExact)
Number of subjects
2
4
6
8
VEP Impact
HIGH
MODERATE
Position [bp] along coding exons (each exon padded by 5 bp)
0
2000
4000
6000
TET2 Mutations
N275Ifs*18
X1137_splice
X1268_splice
I1873T/N
Tet_JBP
2002aa
Exon NM_001127208
TET2 : NM_001127208.3 Q6N021 + ISL
Gln916Ter
N275Ifs*18/Pfs*17
X1268_splice
I1873T
Exon NM_01127208

Panel C

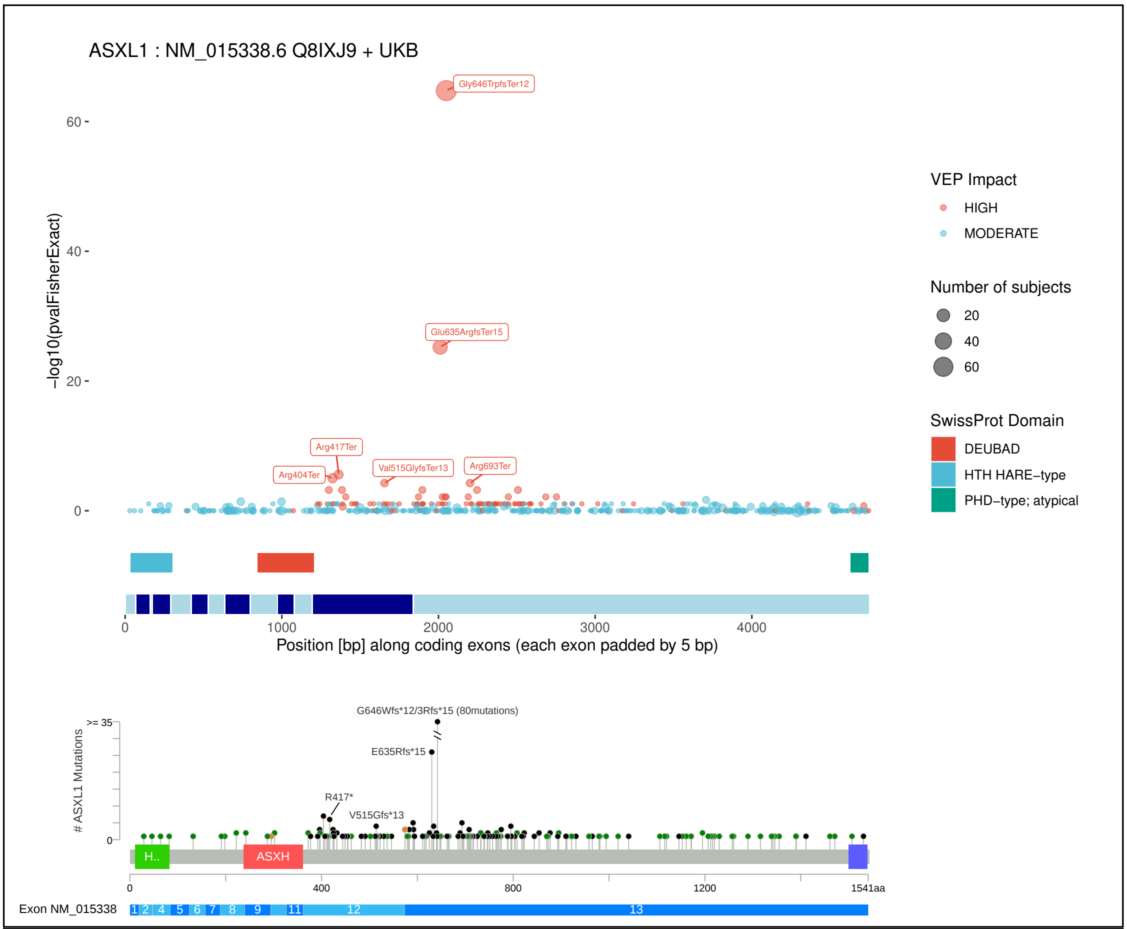
ASXL1 : NM_015338.6 Q8IXJ9 + UKB
Gly646TrpfsTer12
Glu635ArgfsTer15
Arg417Ter
Arg404Ter
Val515GlyfsTer13
Arg693Ter
−log10(pvalFisherExact)
60
40
20
0
VEP Impact
HIGH
MODERATE
Number of subjects
20
40
60
SwissProt Domain
DEUBAD
HTH HARE−type
PHD−type; atypical
Position [bp] along coding exons (each exon padded by 5 bp)
0
1000
2000
3000
4000
G646Wfs*12/3Rfs*15 (80mutations)
E635Rfs*15
R417*
V515Gfs*13
ASXL1 Mutations
>= 35
H..
ASXH
400
800
1200
1541aa
Exon NM_015338
1 2 4 5 6 7 8 9 11 12 13

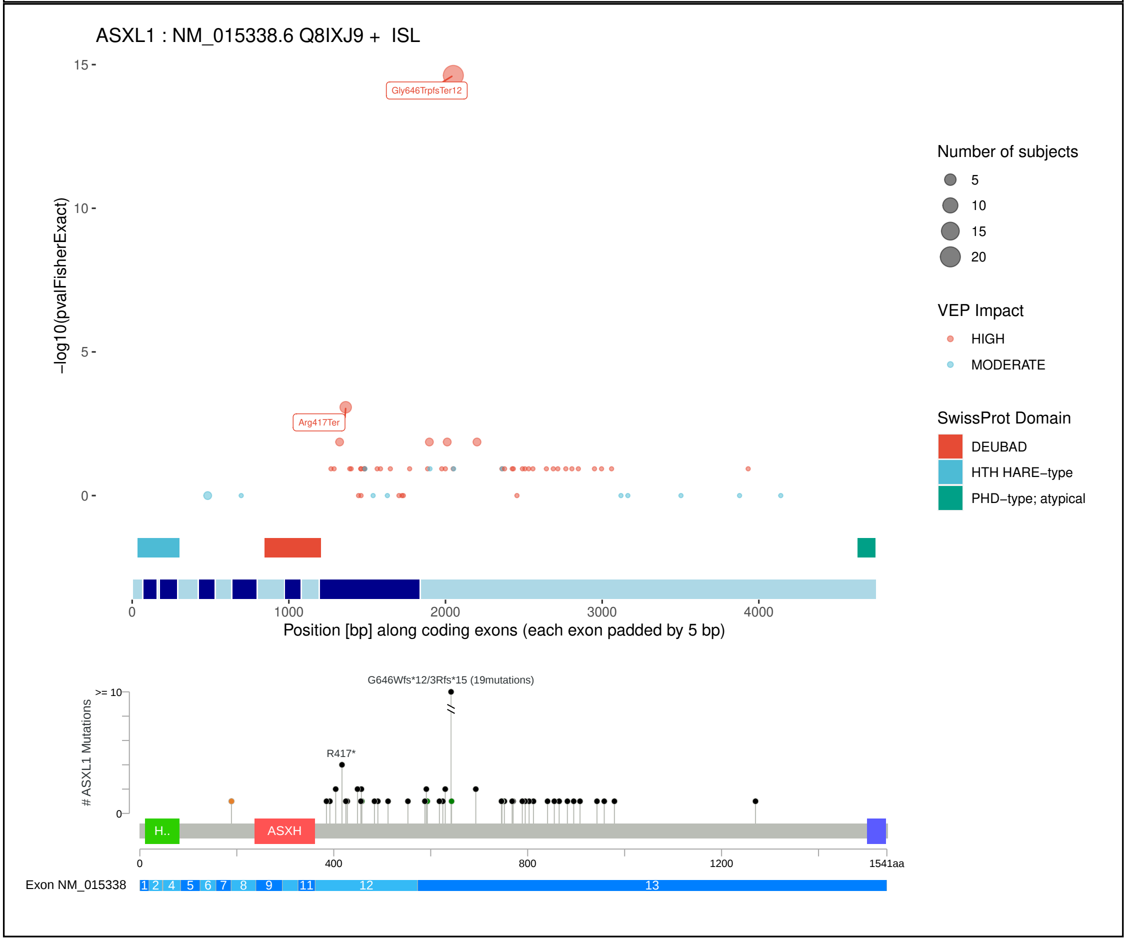
ASXL1 : NM_015338.6 Q8IXJ9 + ISL
Gly646TrpfsTer12
Arg417Ter
−log10(pvalFisherExact)
15
10
5
0
Number of subjects
5
10
15
20
VEP Impact
HIGH
MODERATE
SwissProt Domain
DEUBAD
HTH HARE−type
PHD−type; atypical
Position [bp] along coding exons (each exon padded by 5 bp)
0
1000
2000
3000
4000
G646Wfs*12/3Rfs*15 (19mutations)
R417*
ASXL1 Mutations
>= 10
H..
ASXH
400
800
1200
1541aa
Exon NM_015338
1 2 4 5 6 7 8 9 11 12 13

Panel D

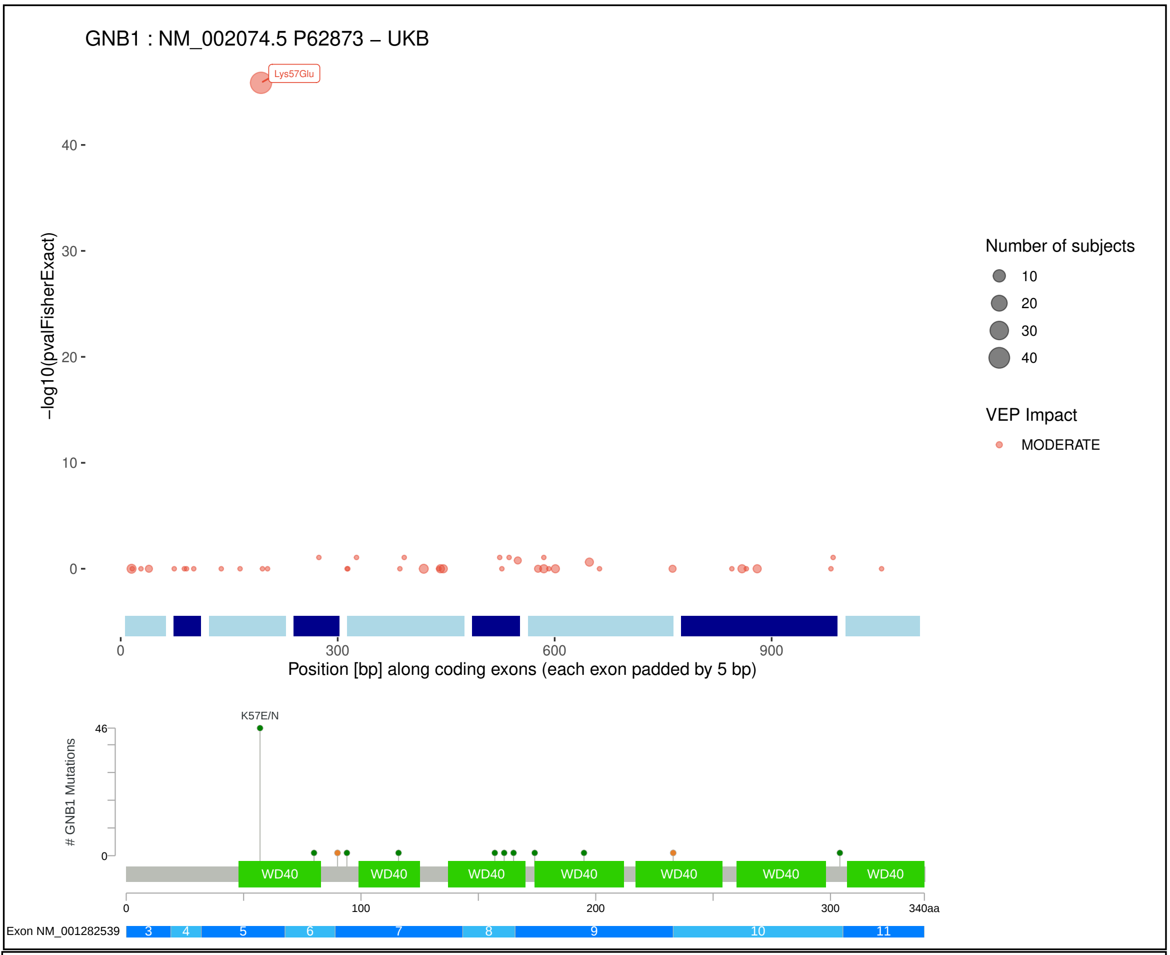
GNB1 : NM_002074.5 P62873 – UKB
Lys57Glu
−log10(pvalFisherExact)
40
30
20
10
0
Number of subjects
10
20
30
40
VEP Impact
MODERATE
0
300
600
900
Position [bp] along coding exons (each exon padded by 5 bp)
K57E/N
46
0
GNB1 Mutations
WD40
WD40
WD40
WD40
WD40
WD40
WD40
0
100
200
300
340aa
Exon NM_001282539
3
4
5
6
7
8
9
10
11

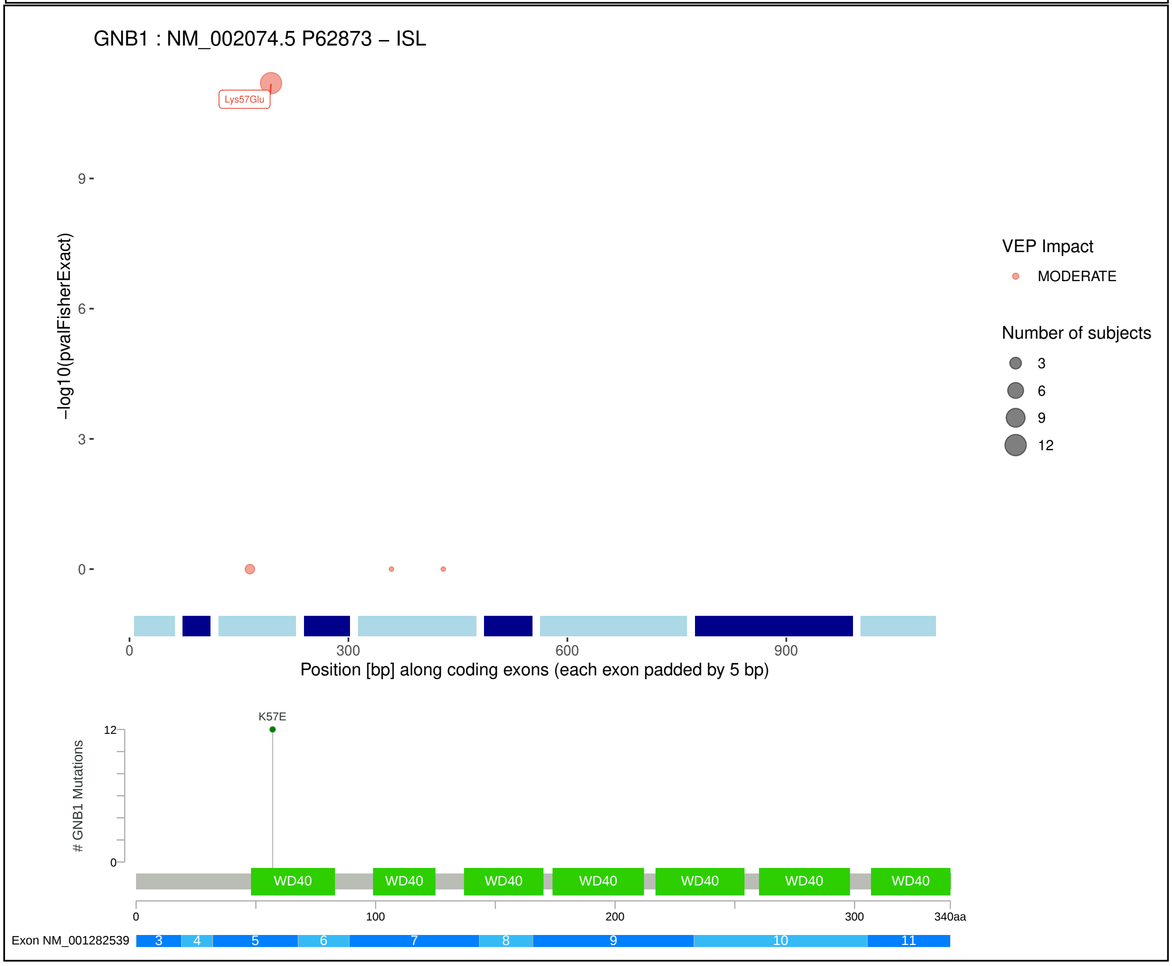
GNB1 : NM_002074.5 P62873 – ISL
Lys57Glu
−log10(pvalFisherExact)
9
6
3
0
VEP Impact
MODERATE
Number of subjects
3
6
9
12
0
300
600
900
Position [bp] along coding exons (each exon padded by 5 bp)
K57E
12
0
GNB1 Mutations
WD40
WD40
WD40
WD40
WD40
WD40
WD40
0
100
200
300
340aa
Exon NM_001282539
3
4
5
6
7
8
9
10
11

Panel E

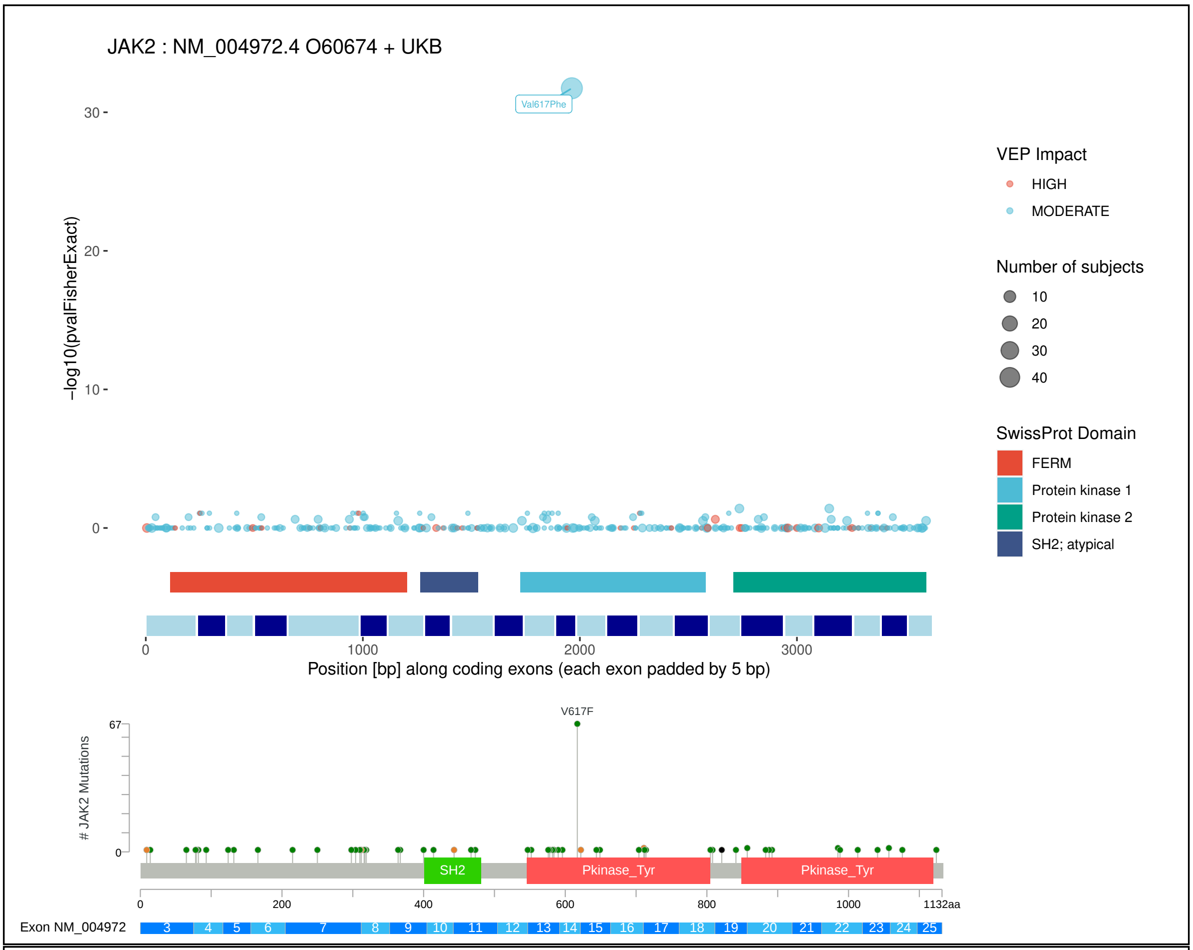

JAK2 : NM_004972.4 O60674 + UKB
Val617Phe
−log10(pvalFisherExact)
30
20
10
0
VEP Impact
HIGH
MODERATE
Number of subjects
10
20
30
40
SwissProt Domain
FERM
Protein kinase 1
Protein kinase 2
SH2; atypical
0
1000
2000
3000
Position [bp] along coding exons (each exon padded by 5 bp)
JAK2 Mutations
67
0
V617F
SH2
Pkinase_Tyr
Pkinase_Tyr
0
200
400
600
800
1000
1132aa
Exon NM_004972
3 4 5 6 7 8 9 10 11 12 13 14 15 16 17 18 19 20 21 22 23 24 25

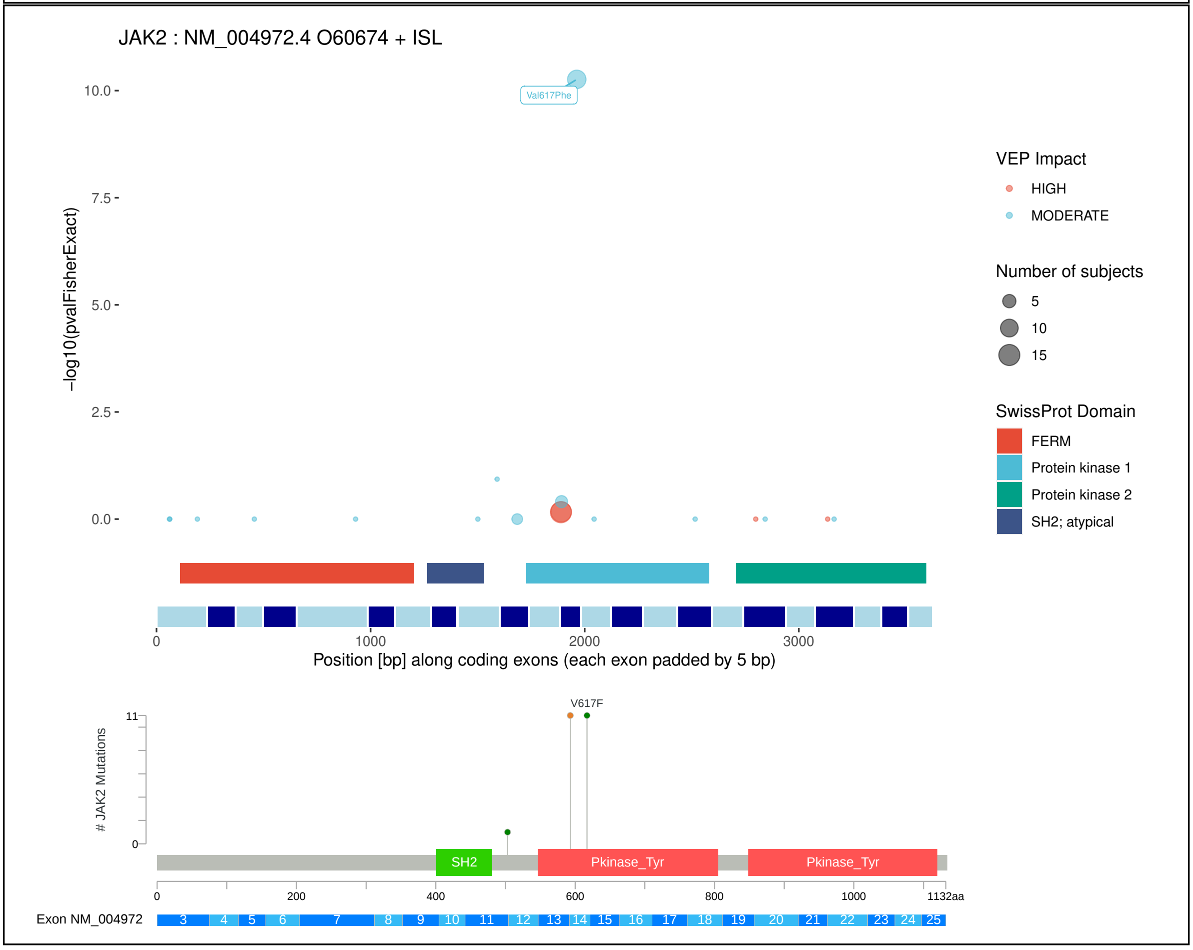

JAK2 : NM_004972.4 O60674 + ISL
Val617Phe
−log10(pvalFisherExact)
10.0
7.5
5.0
2.5
0.0
VEP Impact
HIGH
MODERATE
Number of subjects
5
10
15
SwissProt Domain
FERM
Protein kinase 1
Protein kinase 2
SH2; atypical
0
1000
2000
3000
Position [bp] along coding exons (each exon padded by 5 bp)
JAK2 Mutations
11
0
V617F
SH2
Pkinase_Tyr
Pkinase_Tyr
0
200
400
600
800
1000
1132aa
Exon NM_004972
3 4 5 6 7 8 9 10 11 12 13 14 15 16 17 18 19 20 21 22 23 24 25

Panel F

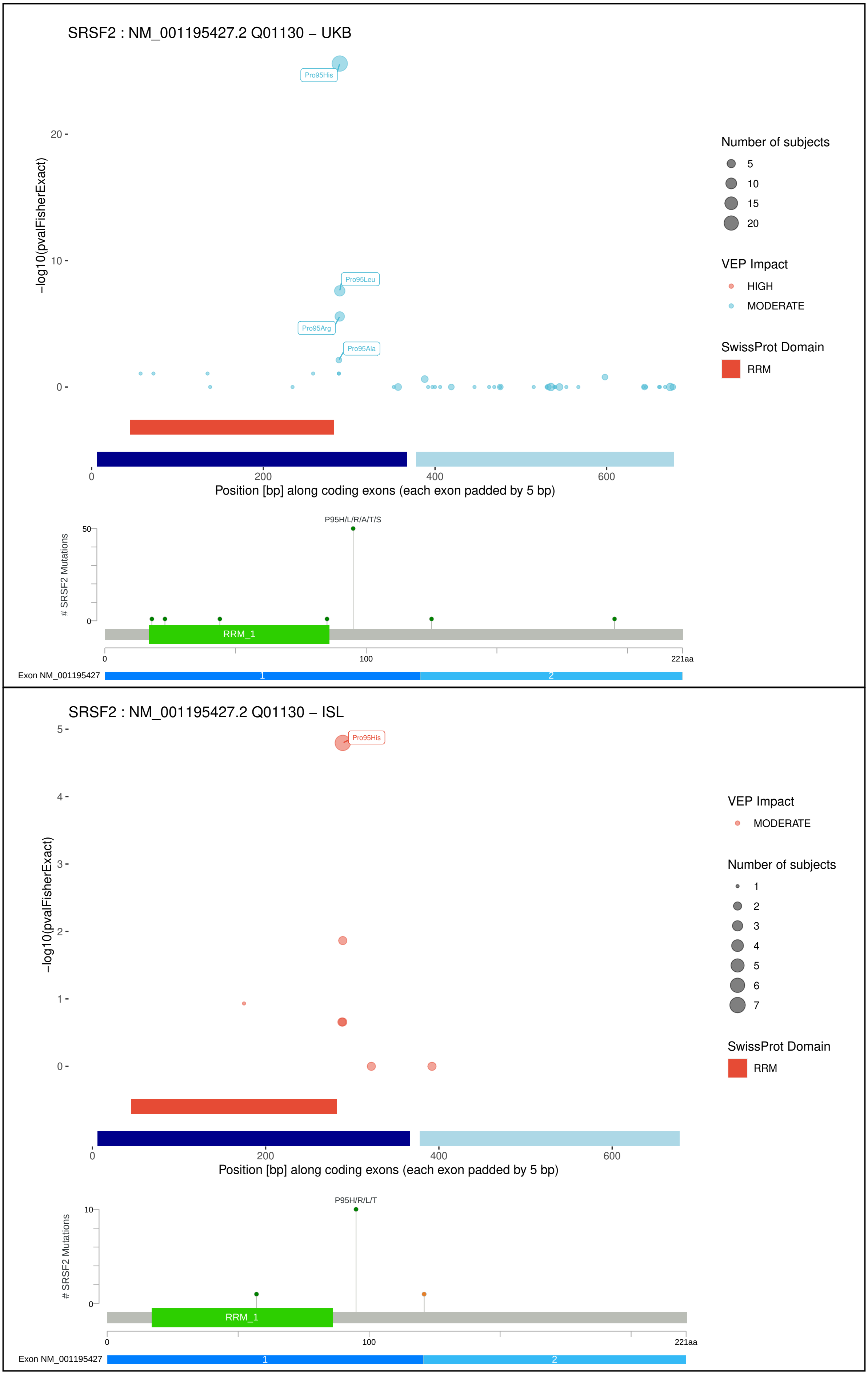
SRSF2 : NM_001195427.2 Q01130 – UKB
Pro95His
Pro95Leu
Pro95Arg
Pro95Ala
−log10(pvalFisherExact)
20
10
0
Number of subjects
5
10
15
20
VEP Impact
HIGH
MODERATE
SwissProt Domain
RRM
0
200
400
600
Position [bp] along coding exons (each exon padded by 5 bp)
P95H/L/R/A/T/S
SRSF2 Mutations
50
0
RRM_1
0
100
221aa
Exon NM_001195427
1
2
SRSF2 : NM_001195427.2 Q01130 – ISL
Pro95His
−log10(pvalFisherExact)
5
4
3
2
1
0
VEP Impact
MODERATE
Number of subjects
1
2
3
4
5
6
7
SwissProt Domain
RRM
0
200
400
600
Position [bp] along coding exons (each exon padded by 5 bp)
P95H/R/L/T
SRSF2 Mutations
10
0
RRM_1
0
100
221aa
Exon NM_001195427
1
2

Panel G

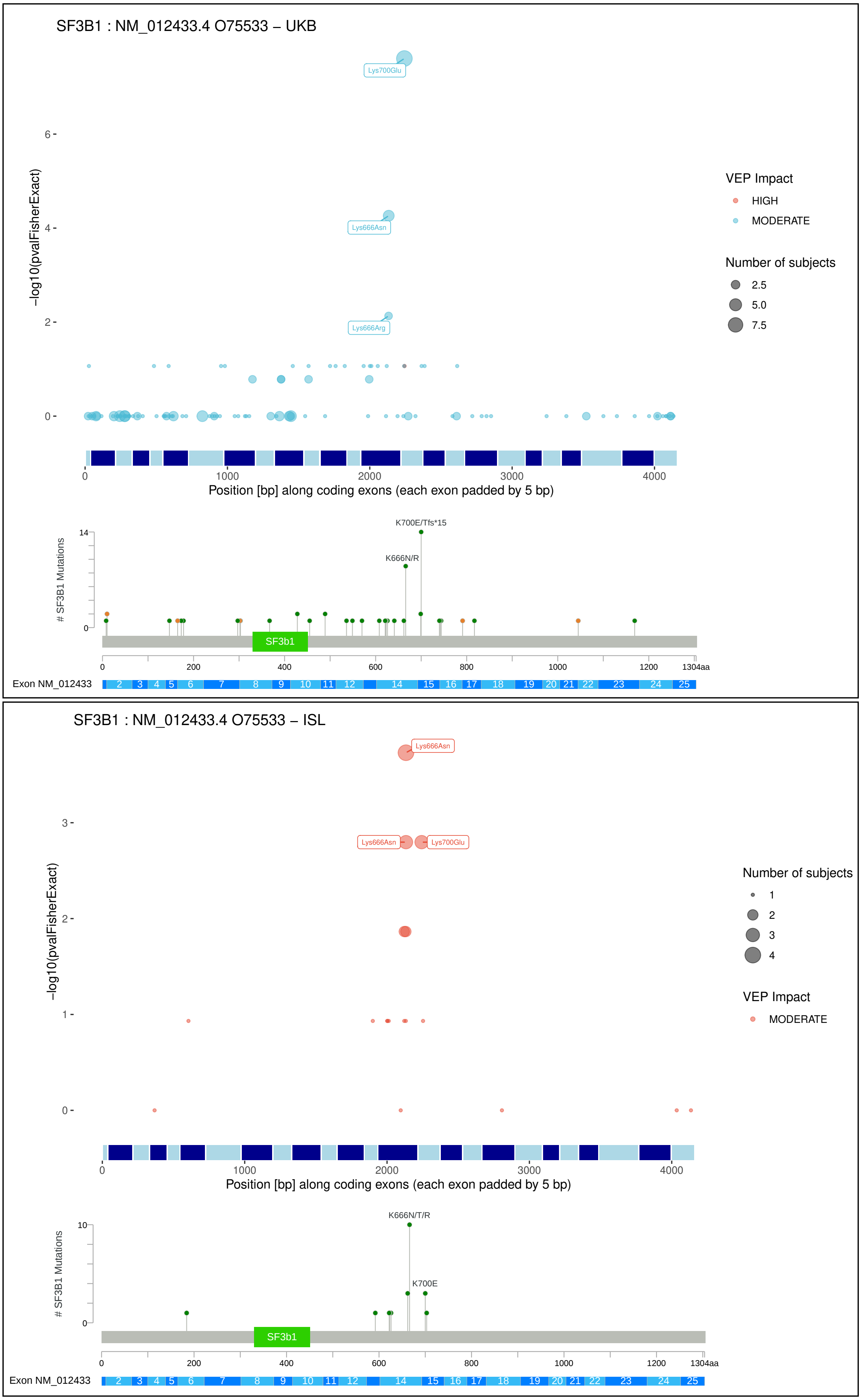
SF3B1 : NM_012433.4 O75533 – UKB
Lys700Glu
Lys666Asn
Lys666Arg
−log10(pvalFisherExact)
Position [bp] along coding exons (each exon padded by 5 bp)
VEP Impact
HIGH
MODERATE
Number of subjects
2.5
5.0
7.5
SF3B1 Mutations
K700E/Tfs*15
K666N/R
SF3b1
1304aa
Exon NM_012433
SF3B1 : NM_012433.4 O75533 – ISL
Lys666Asn
Lys700Glu
−log10(pvalFisherExact)
Number of subjects
1
2
3
4
VEP Impact
MODERATE
Position [bp] along coding exons (each exon padded by 5 bp)
SF3B1 Mutations
K666N/T/R
K700E
SF3b1
1304aa
Exon NM_012433

Panel H

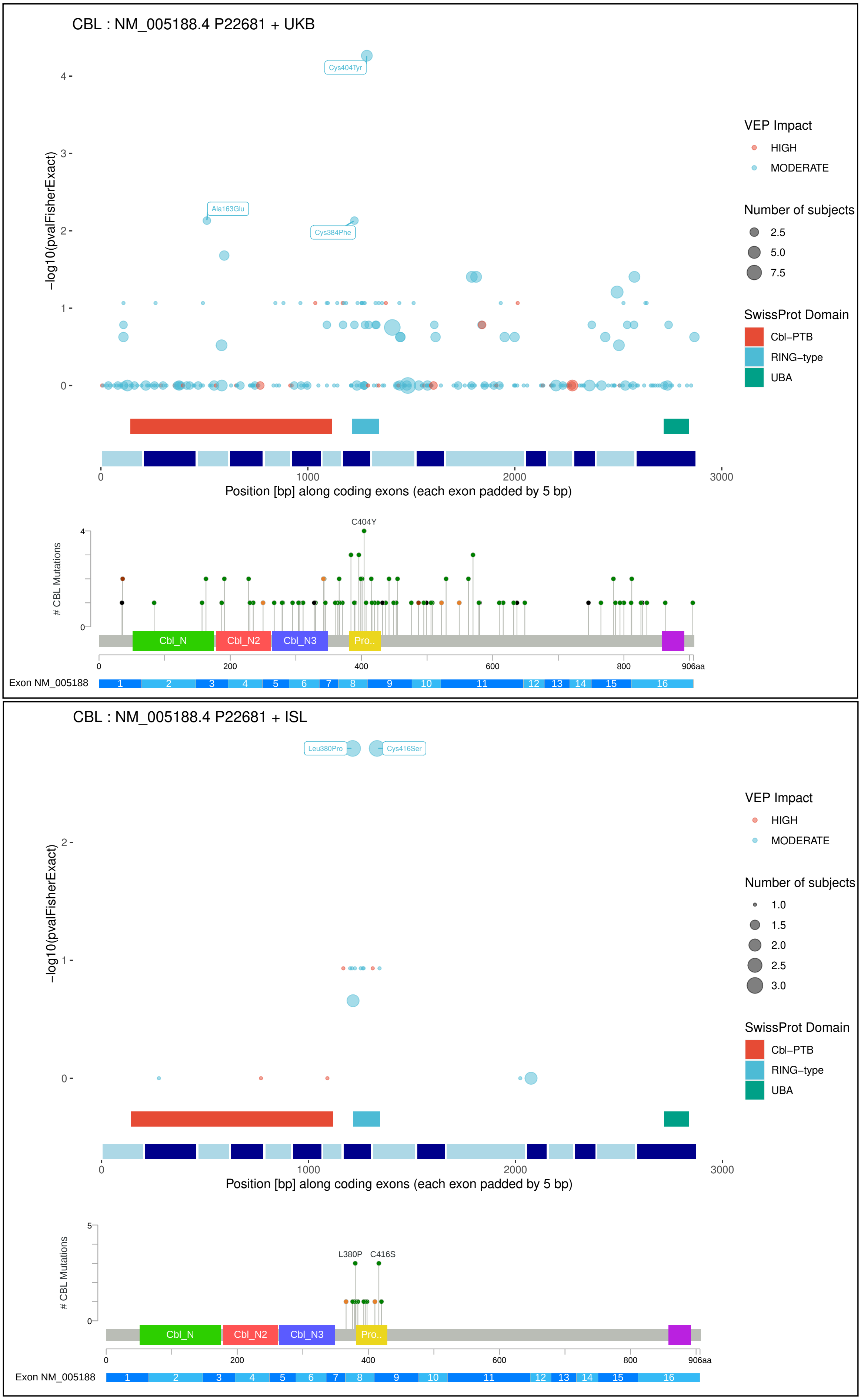
CBL : NM_005188.4 P22681 + UKB
Cys404Tyr
Ala163Glu
Cys384Phe
−log10(pvalFisherExact)
Position [bp] along coding exons (each exon padded by 5 bp)
VEP Impact
HIGH
MODERATE
Number of subjects
2.5
5.0
7.5
SwissProt Domain
Cbl−PTB
RING−type
UBA
C404Y
CBL Mutations
Cbl_N
Cbl_N2
Cbl_N3
Pro..
906aa
Exon NM_005188
CBL : NM_005188.4 P22681 + ISL
Leu380Pro
Cys416Ser
−log10(pvalFisherExact)
Position [bp] along coding exons (each exon padded by 5 bp)
VEP Impact
HIGH
MODERATE
Number of subjects
1.0
1.5
2.0
2.5
3.0
SwissProt Domain
Cbl−PTB
RING−type
UBA
L380P
C416S
CBL Mutations
Cbl_N
Cbl_N2
Cbl_N3
Pro..
906aa
Exon NM_005188

Panel I

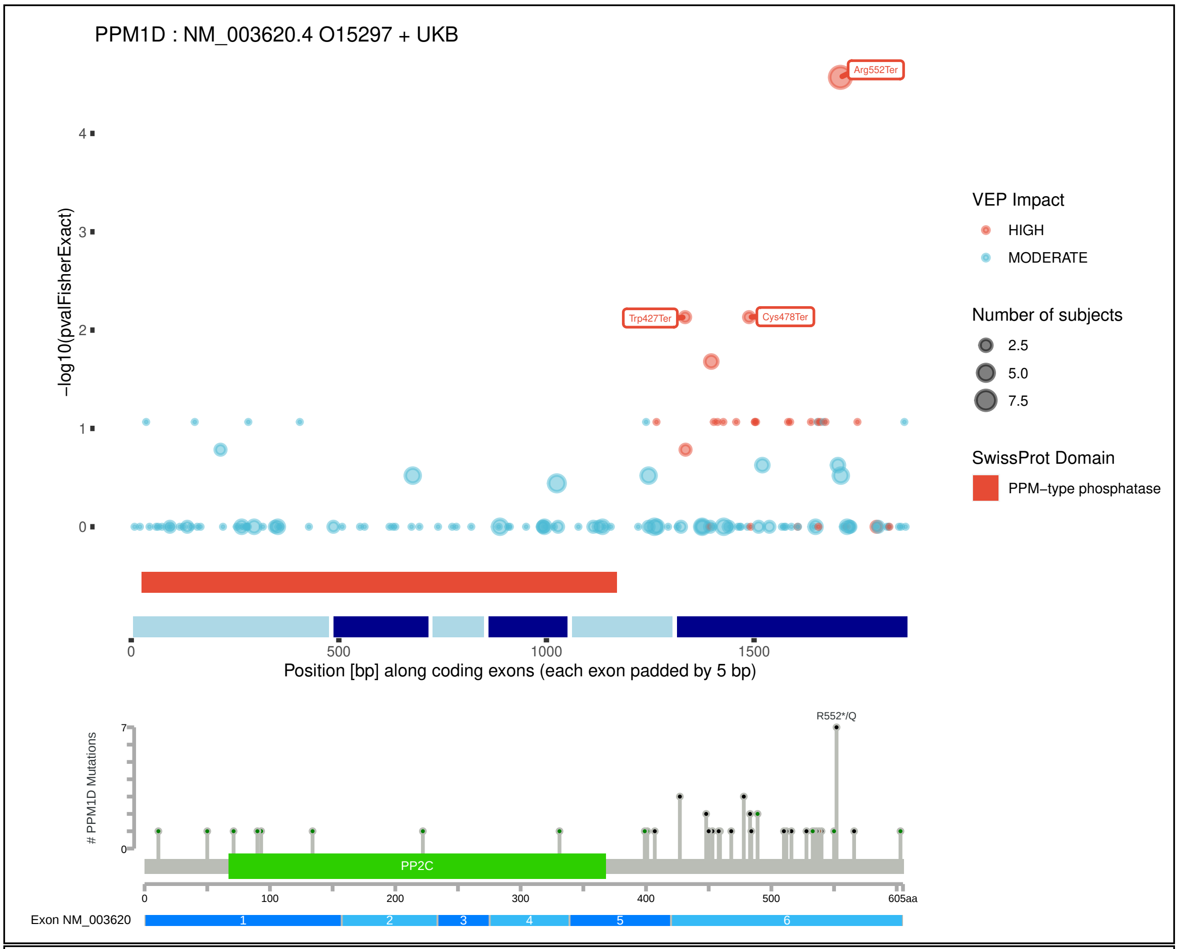
PPM1D : NM_003620.4 O15297 + UKB
Arg552Ter
Trp427Ter
Cys478Ter
−log10(pvalFisherExact)
0
1
2
3
4
0
500
1000
1500
Position [bp] along coding exons (each exon padded by 5 bp)
VEP Impact
HIGH
MODERATE
Number of subjects
2.5
5.0
7.5
SwissProt Domain
PPM−type phosphatase
R552*/Q
PPM1D Mutations
7
0
PP2C
0
100
200
300
400
500
605aa
Exon NM_003620
1
2
3
4
5
6

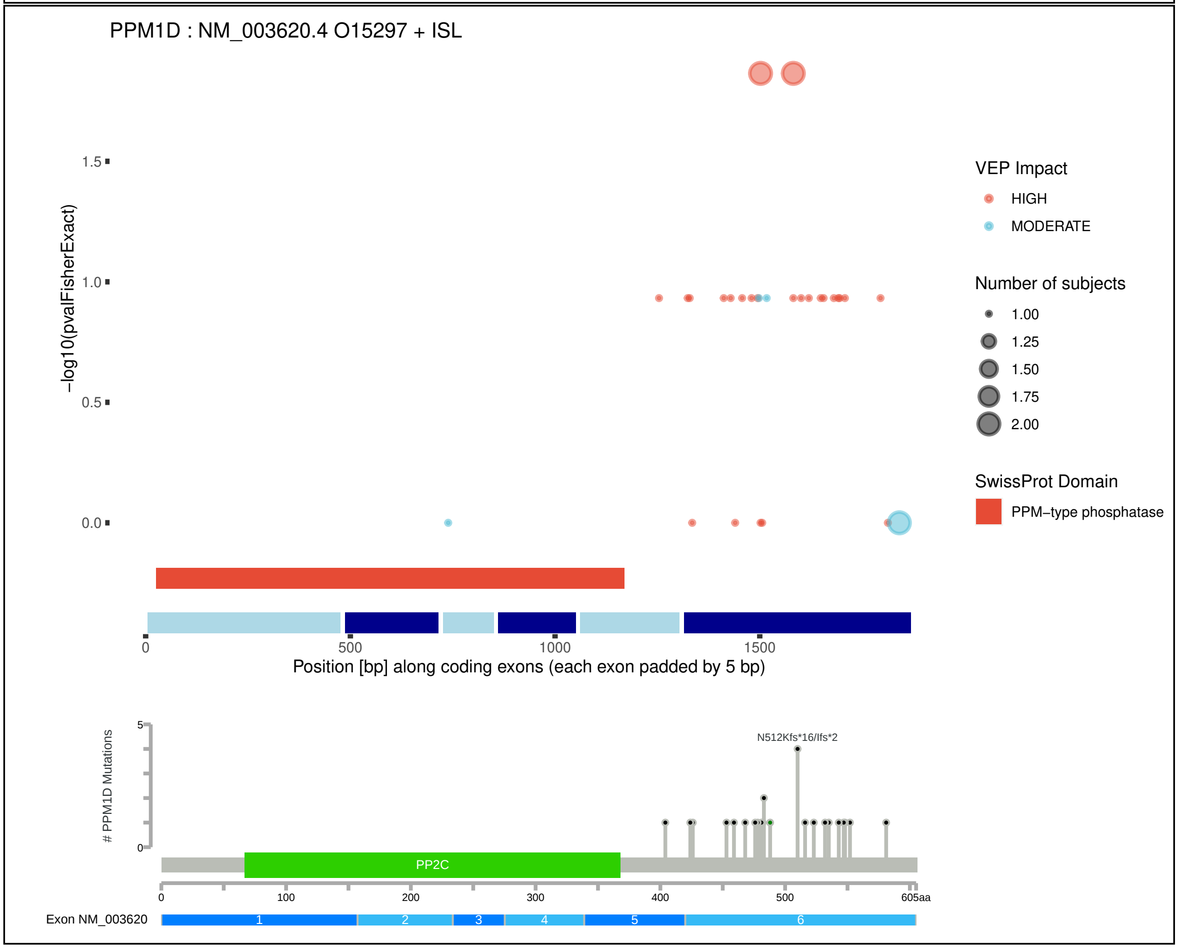
PPM1D : NM_003620.4 O15297 + ISL
−log10(pvalFisherExact)
0.0
0.5
1.0
1.5
0
500
1000
1500
Position [bp] along coding exons (each exon padded by 5 bp)
VEP Impact
HIGH
MODERATE
Number of subjects
1.00
1.25
1.50
1.75
2.00
SwissProt Domain
PPM−type phosphatase
N512Kfs*16/Ifs*2
PPM1D Mutations
5
0
PP2C
0
100
200
300
400
500
605aa
Exon NM_003620
1
2
3
4
5
6

Panel J

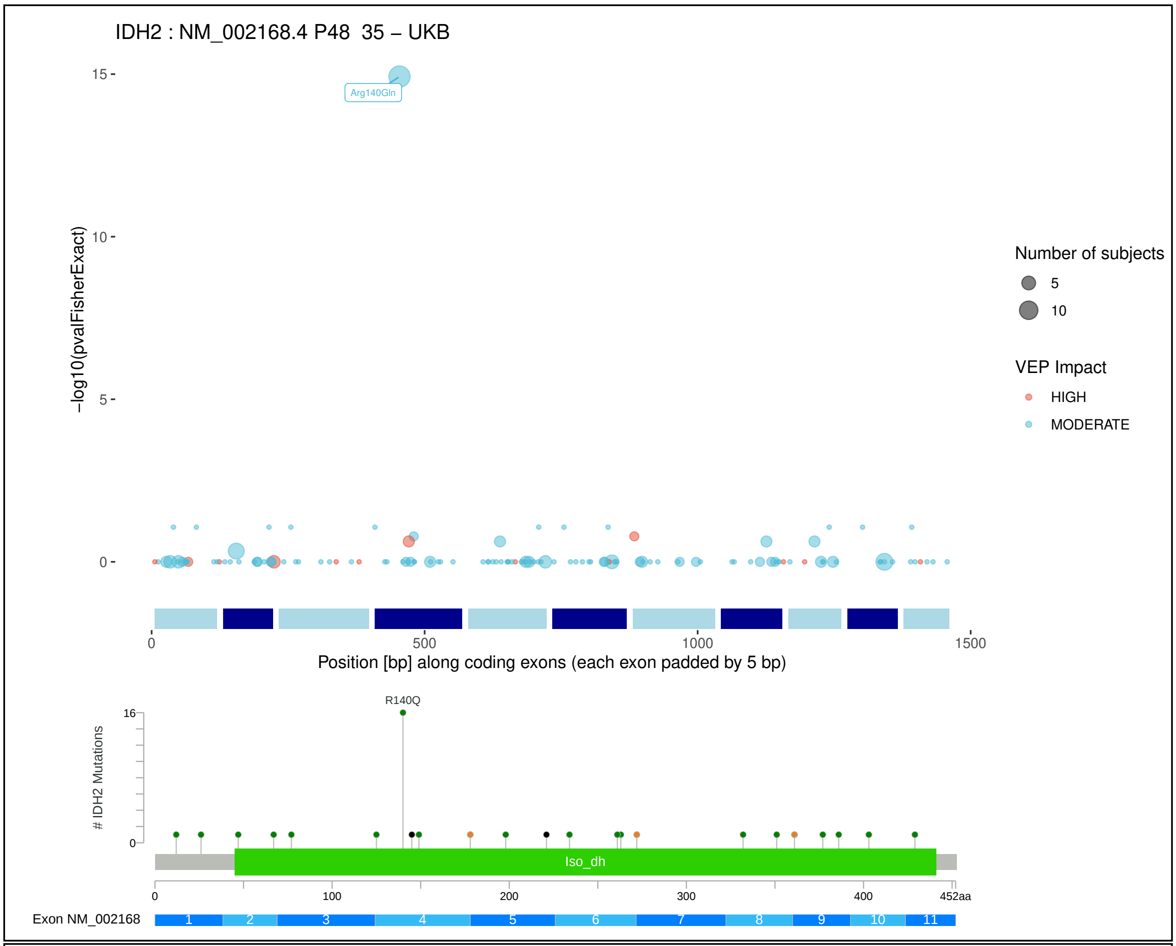
IDH2 : NM_002168.4 P48 35 – UKB
Arg140Gln
−log10(pvalFisherExact)
15
10
5
0
Number of subjects
5
10
VEP Impact
HIGH
MODERATE
0
500
1000
1500
Position [bp] along coding exons (each exon padded by 5 bp)
R140Q
16
0
IDH2 Mutations
Iso_dh
0
100
200
300
400
452aa
Exon NM_002168
1
2
3
4
5
6
7
8
9
10
11

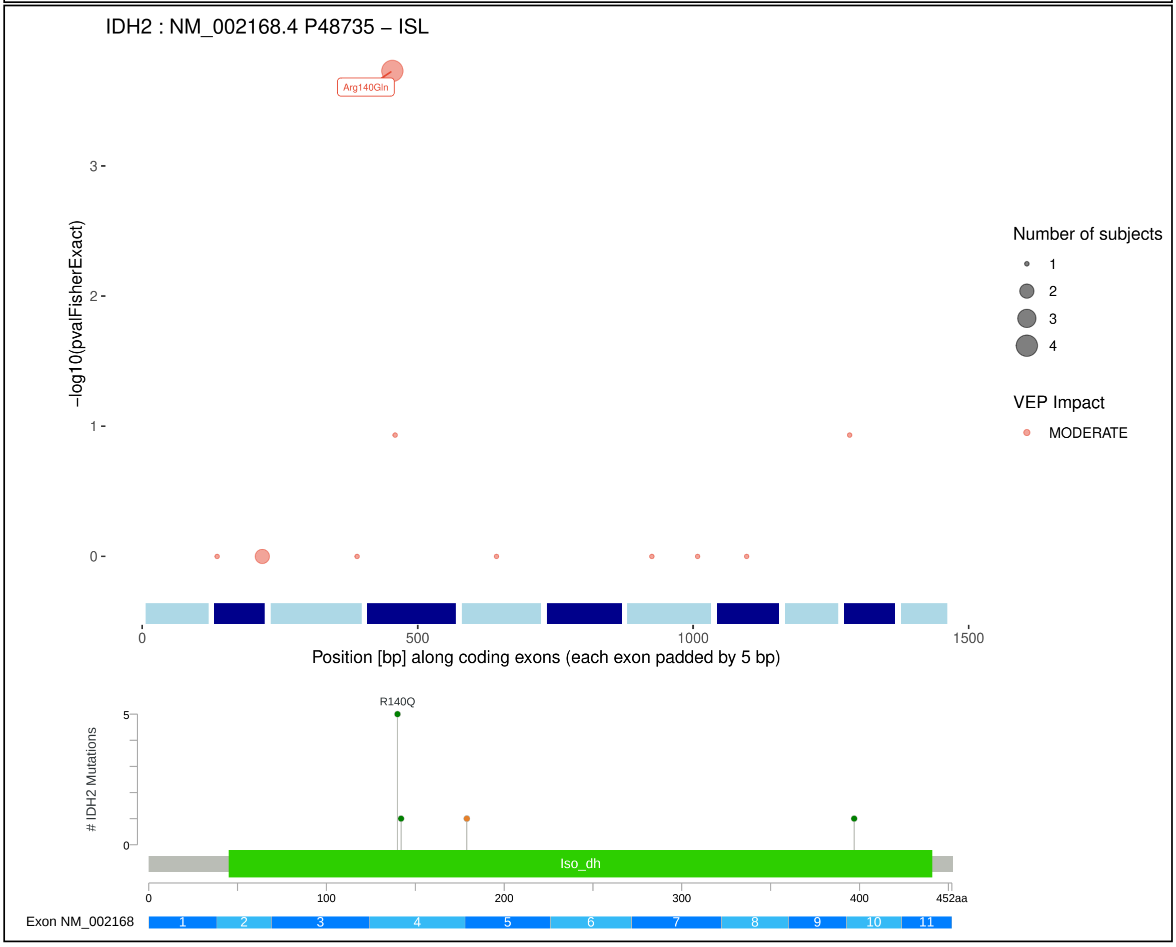
IDH2 : NM_002168.4 P48735 – ISL
Arg140Gln
−log10(pvalFisherExact)
3
2
1
0
Number of subjects
1
2
3
4
VEP Impact
MODERATE
0
500
1000
1500
Position [bp] along coding exons (each exon padded by 5 bp)
R140Q
5
0
IDH2 Mutations
Iso_dh
0
100
200
300
400
452aa
Exon NM_002168
1
2
3
4
5
6
7
8
9
10
11

Panel K

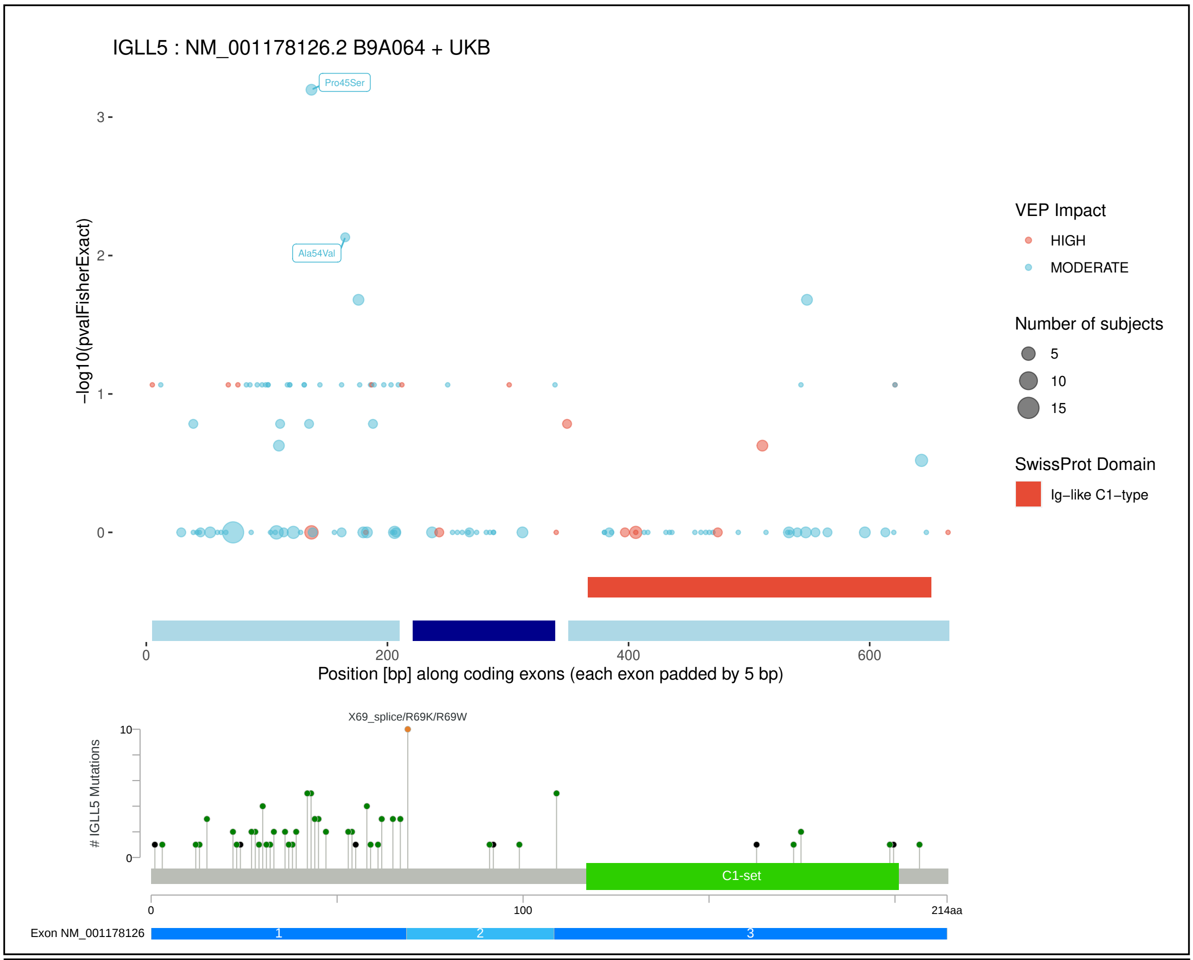

IGLL5 : NM_001178126.2 B9A064 + UKB
Pro45Ser
Ala54Val
−log10(pvalFisherExact)
Position [bp] along coding exons (each exon padded by 5 bp)
VEP Impact
HIGH
MODERATE
Number of subjects
5
10
15
SwissProt Domain
Ig−like C1−type
X69_splice/R69K/R69W
IGLL5 Mutations
C1-set
214aa
Exon NM_001178126

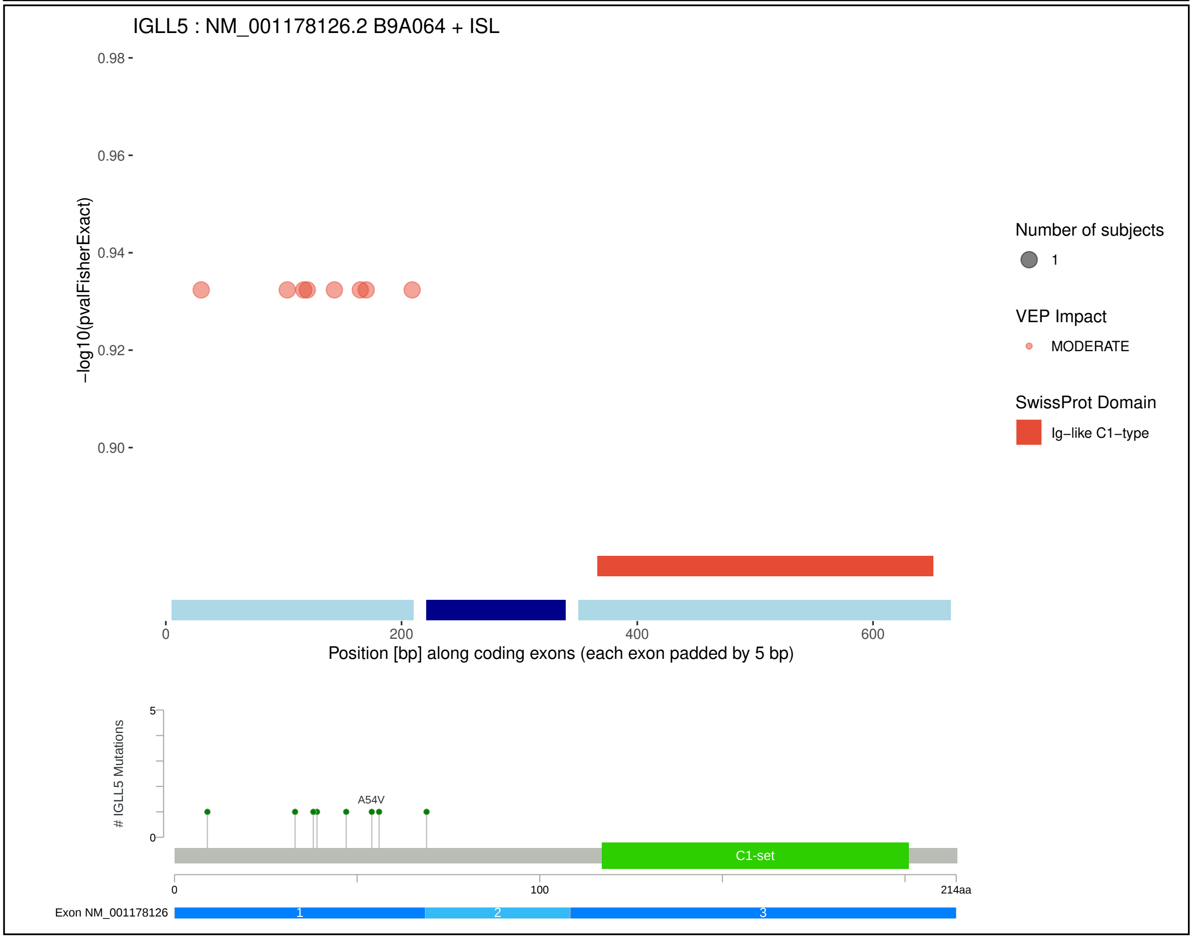

IGLL5 : NM_001178126.2 B9A064 + ISL
−log10(pvalFisherExact)
Position [bp] along coding exons (each exon padded by 5 bp)
Number of subjects
1
VEP Impact
MODERATE
SwissProt Domain
Ig−like C1−type
A54V
IGLL5 Mutations
C1-set
214aa
Exon NM_001178126

Panel L

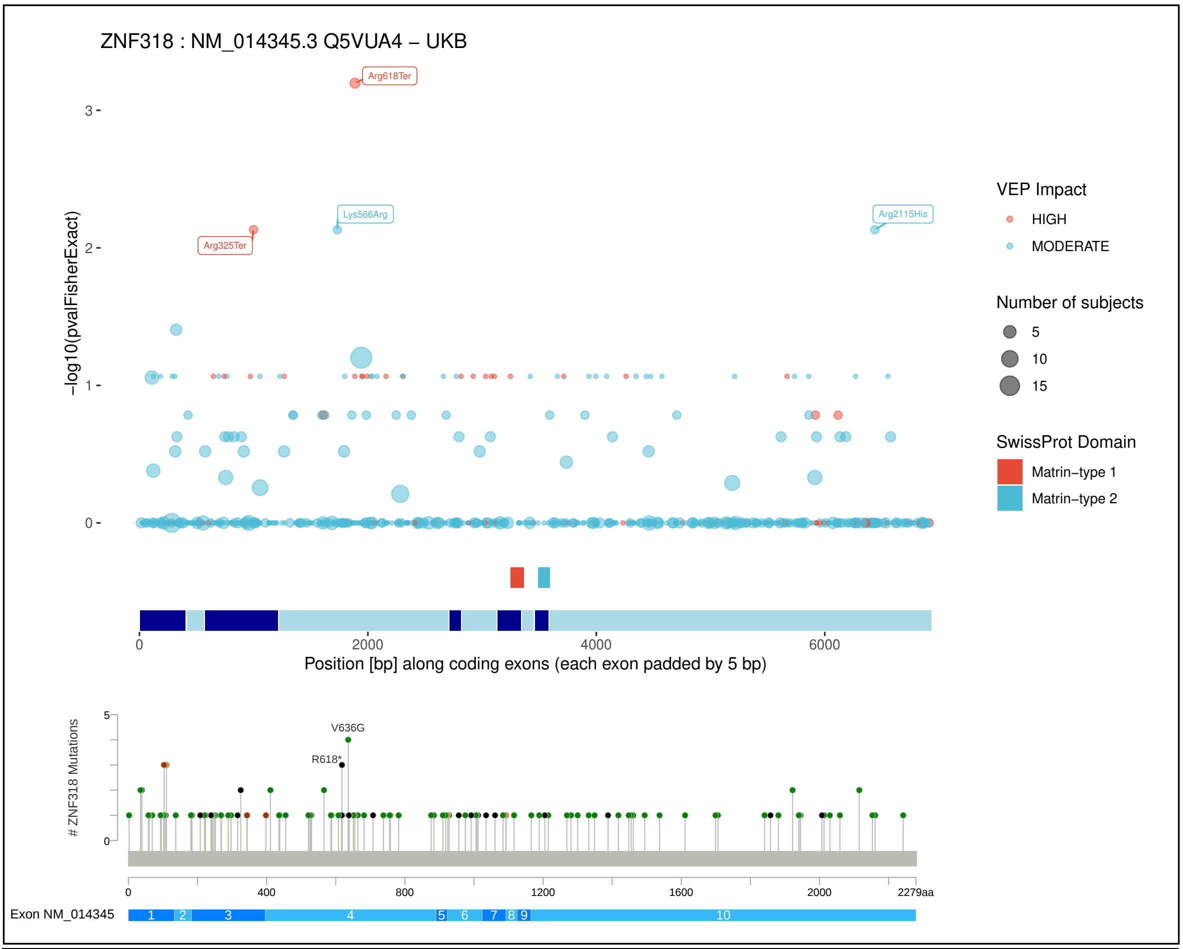

ZNF318 : NM_014345.3 Q5VUA4 – UKB
Arg618Ter
Lys566Arg
Arg2115His
Arg325Ter
−log10(pvalFisherExact)
Position [bp] along coding exons (each exon padded by 5 bp)
VEP Impact
HIGH
MODERATE
Number of subjects
5
10
15
SwissProt Domain
Matrin−type 1
Matrin−type 2
ZNF318 Mutations
V636G
R618*
2279aa
Exon NM_014345

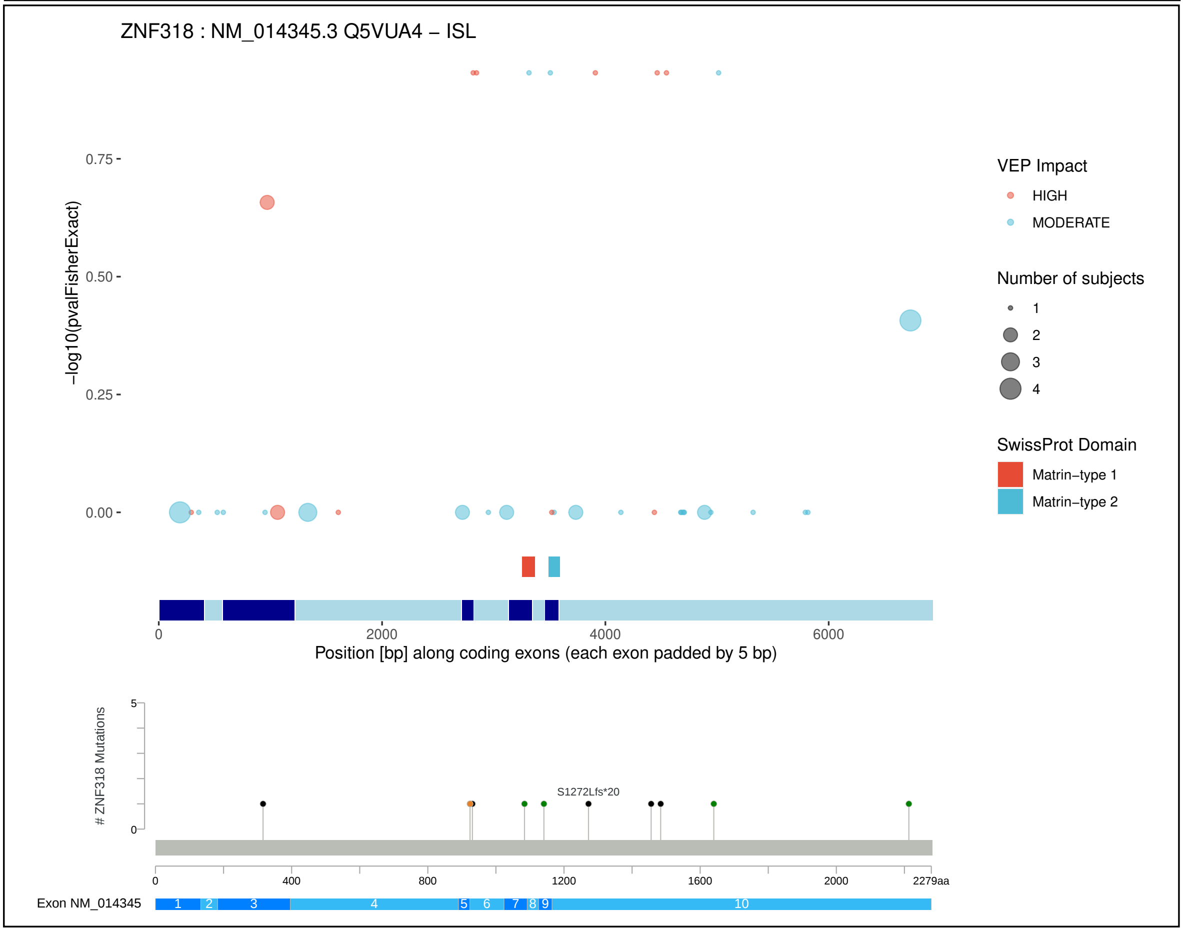

ZNF318 : NM_014345.3 Q5VUA4 – ISL
−log10(pvalFisherExact)
Position [bp] along coding exons (each exon padded by 5 bp)
VEP Impact
HIGH
MODERATE
Number of subjects
1
2
3
4
SwissProt Domain
Matrin−type 1
Matrin−type 2
ZNF318 Mutations
S1272Lfs*20
2279aa
Exon NM_014345

Panel M

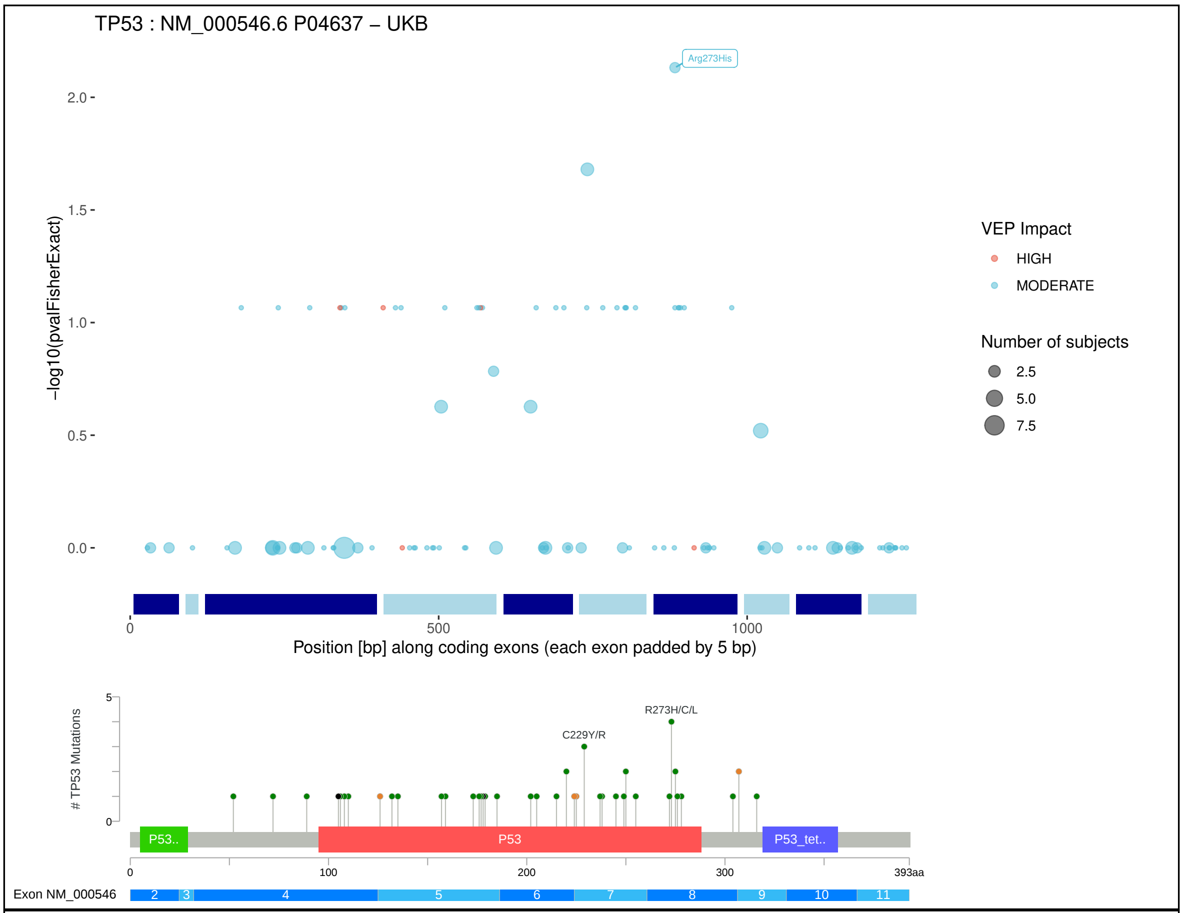
TP53 : NM_000546.6 P04637 – UKB
Arg273His
−log10(pvalFisherExact)
2.0
1.5
1.0
0.5
0.0
VEP Impact
HIGH
MODERATE
Number of subjects
2.5
5.0
7.5
0
500
1000
Position [bp] along coding exons (each exon padded by 5 bp)
TP53 Mutations
R273H/C/L
C229Y/R
P53..
P53
P53_tet..
100
200
300
393aa
Exon NM_000546
2
3
4
5
6
7
8
9
10
11

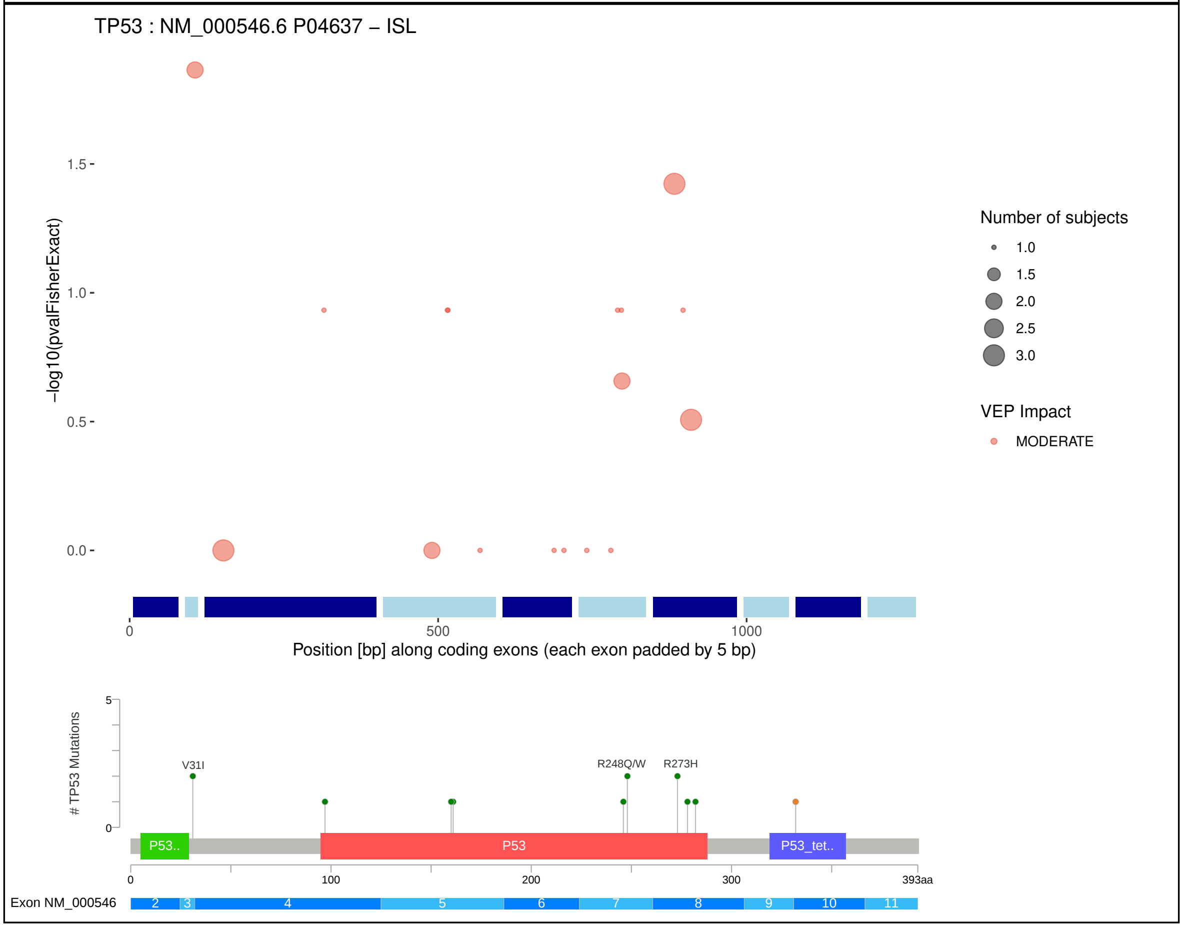
TP53 : NM_000546.6 P04637 – ISL
−log10(pvalFisherExact)
1.5
1.0
0.5
0.0
Number of subjects
1.0
1.5
2.0
2.5
3.0
VEP Impact
MODERATE
0
500
1000
Position [bp] along coding exons (each exon padded by 5 bp)
TP53 Mutations
V31I
R248Q/W
R273H
P53..
P53
P53_tet..
100
200
300
393aa
Exon NM_000546
2
3
4
5
6
7
8
9
10
11

Panel N

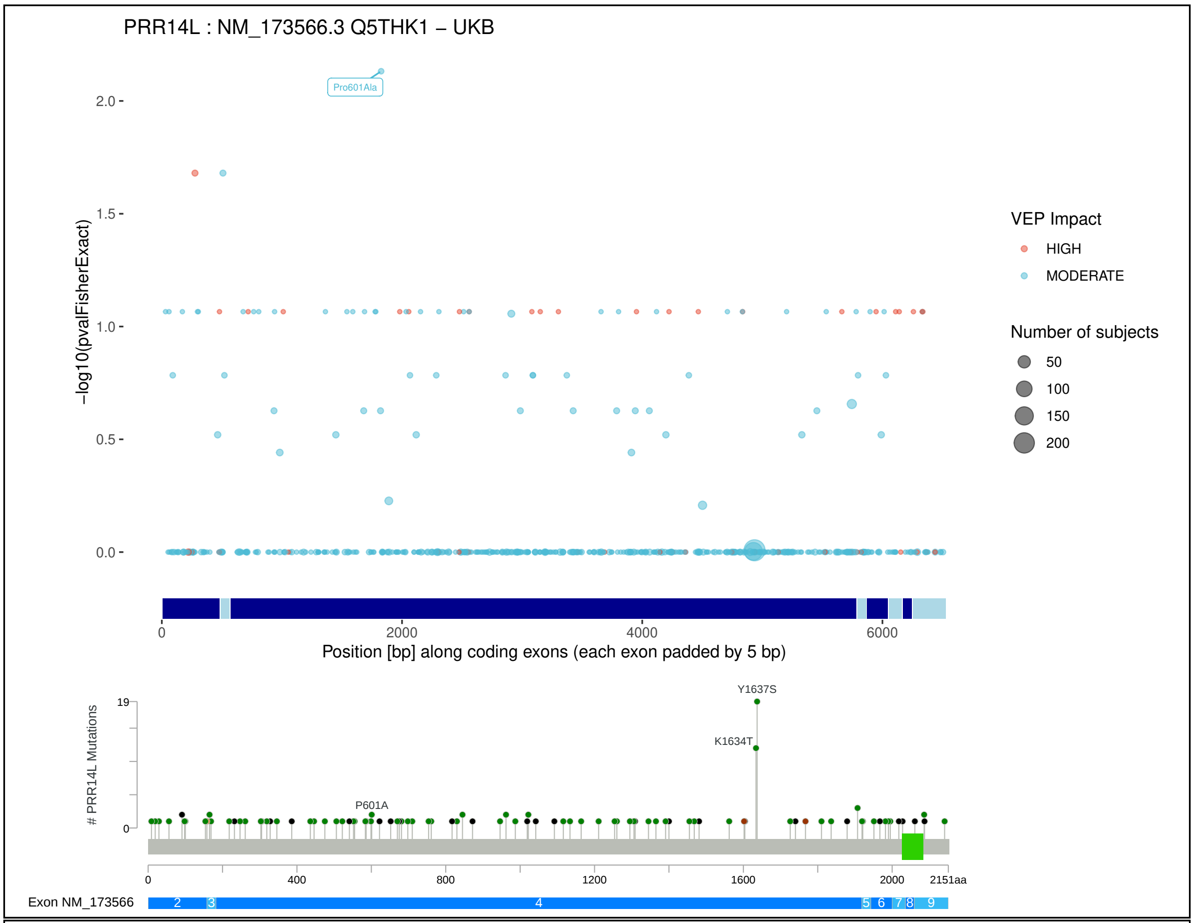

PRR14L : NM_173566.3 Q5THK1 – UKB
Pro601Ala
−log10(pvalFisherExact)
2.0
1.5
1.0
0.5
0.0
0
2000
4000
6000
Position [bp] along coding exons (each exon padded by 5 bp)
VEP Impact
HIGH
MODERATE
Number of subjects
50
100
150
200
PRR14L Mutations
19
0
Y1637S
K1634T
P601A
0
400
800
1200
1600
2000
2151aa
Exon NM_173566
2
3
4
5
6
7
8
9

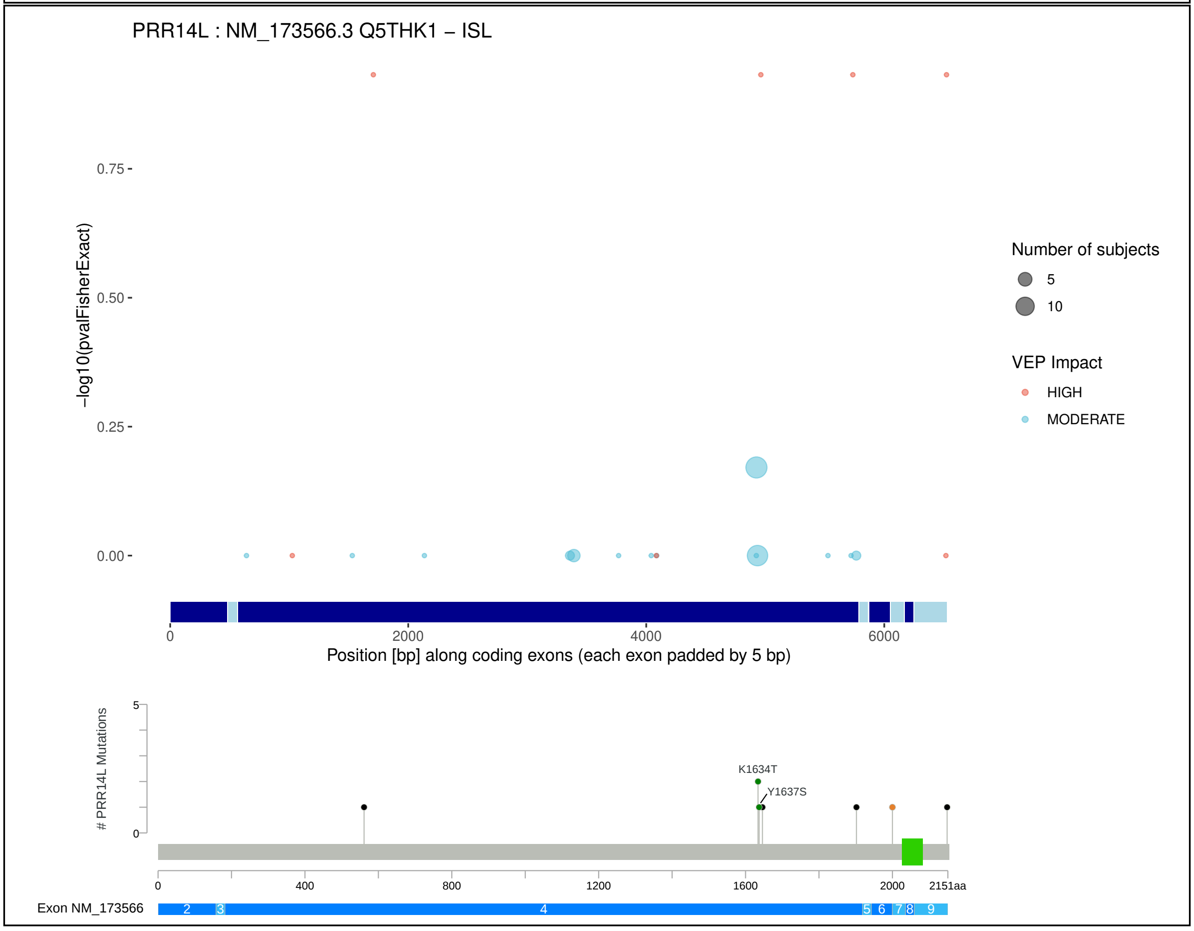

PRR14L : NM_173566.3 Q5THK1 – ISL
−log10(pvalFisherExact)
0.75
0.50
0.25
0.00
0
2000
4000
6000
Position [bp] along coding exons (each exon padded by 5 bp)
Number of subjects
5
10
VEP Impact
HIGH
MODERATE
PRR14L Mutations
5
0
K1634T
Y1637S
0
400
800
1200
1600
2000
2151aa
Exon NM_173566
2
3
4
5
6
7
8
9

Panel O

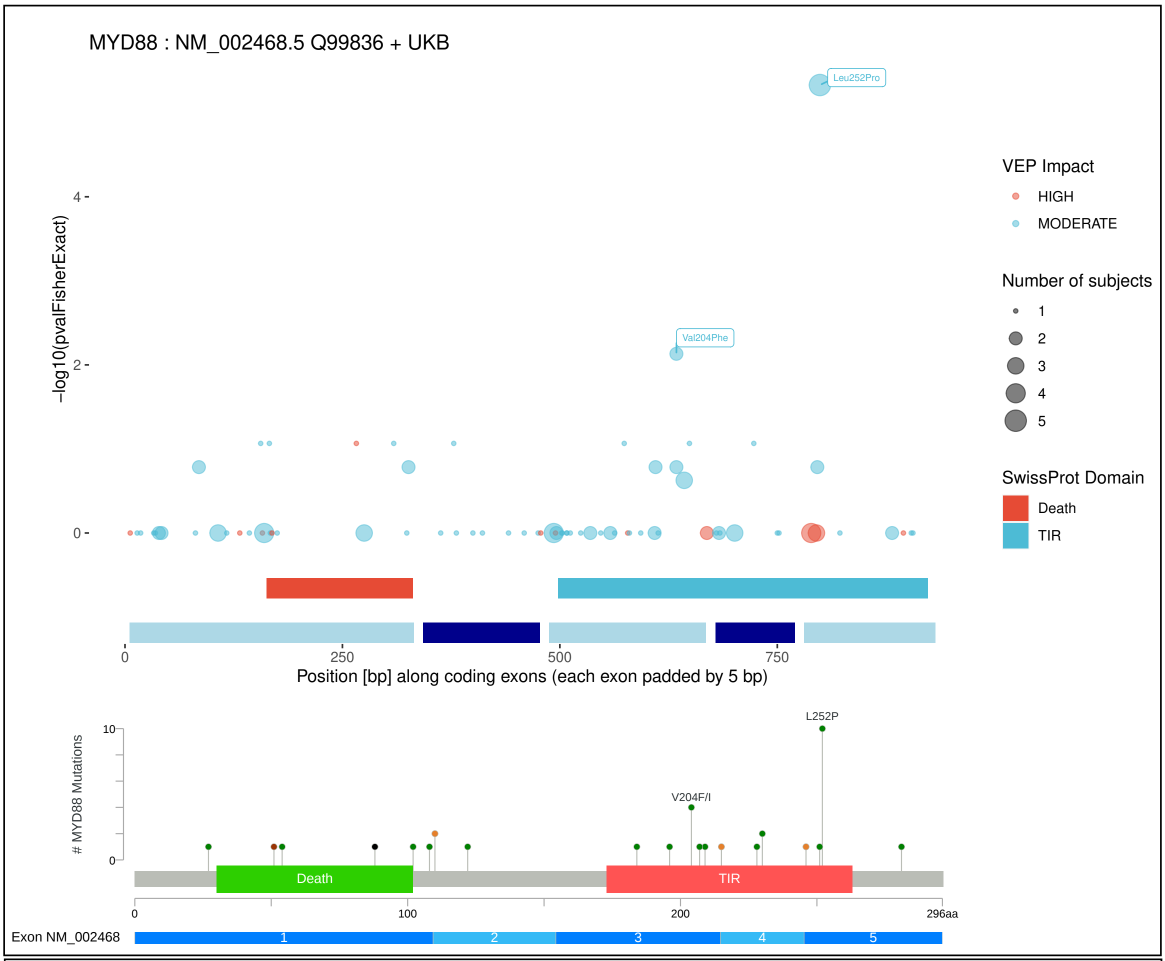

MYD88 : NM_002468.5 Q99836 + UKB
Leu252Pro
Val204Phe
-log10(pvalFisherExact)
4
2
0
VEP Impact
HIGH
MODERATE
Number of subjects
1
2
3
4
5
SwissProt Domain
Death
TIR
0
250
500
750
Position [bp] along coding exons (each exon padded by 5 bp)
MYD88 Mutations
10
0
L252P
V204F/I
Death
TIR
0
100
200
296aa
Exon NM_002468
1
2
3
4
5

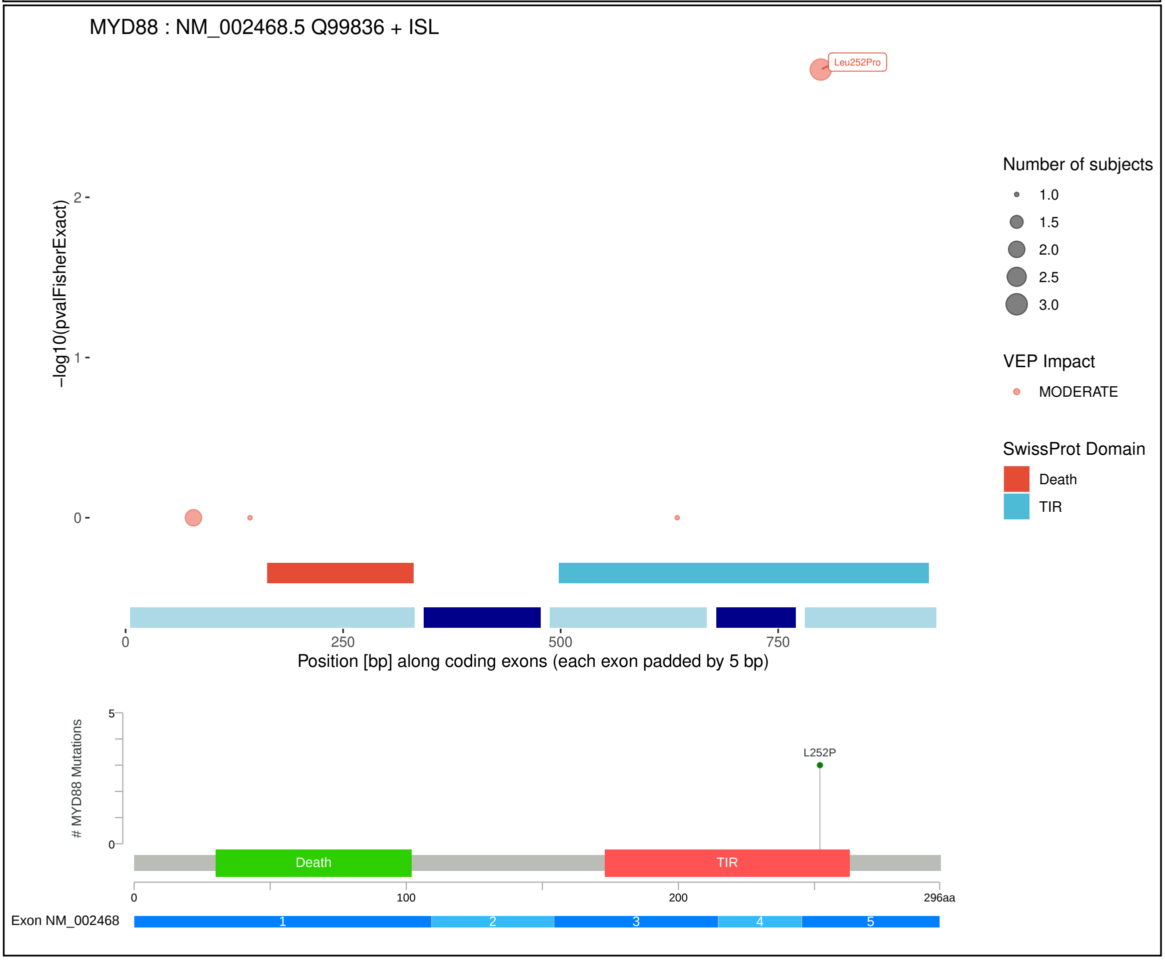

MYD88 : NM_002468.5 Q99836 + ISL
Leu252Pro
-log10(pvalFisherExact)
2
1
0
Number of subjects
1.0
1.5
2.0
2.5
3.0
VEP Impact
MODERATE
SwissProt Domain
Death
TIR
0
250
500
750
Position [bp] along coding exons (each exon padded by 5 bp)
MYD88 Mutations
5
0
L252P
Death
TIR
0
100
200
296aa
Exon NM_002468
1
2
3
4
5

Panel P

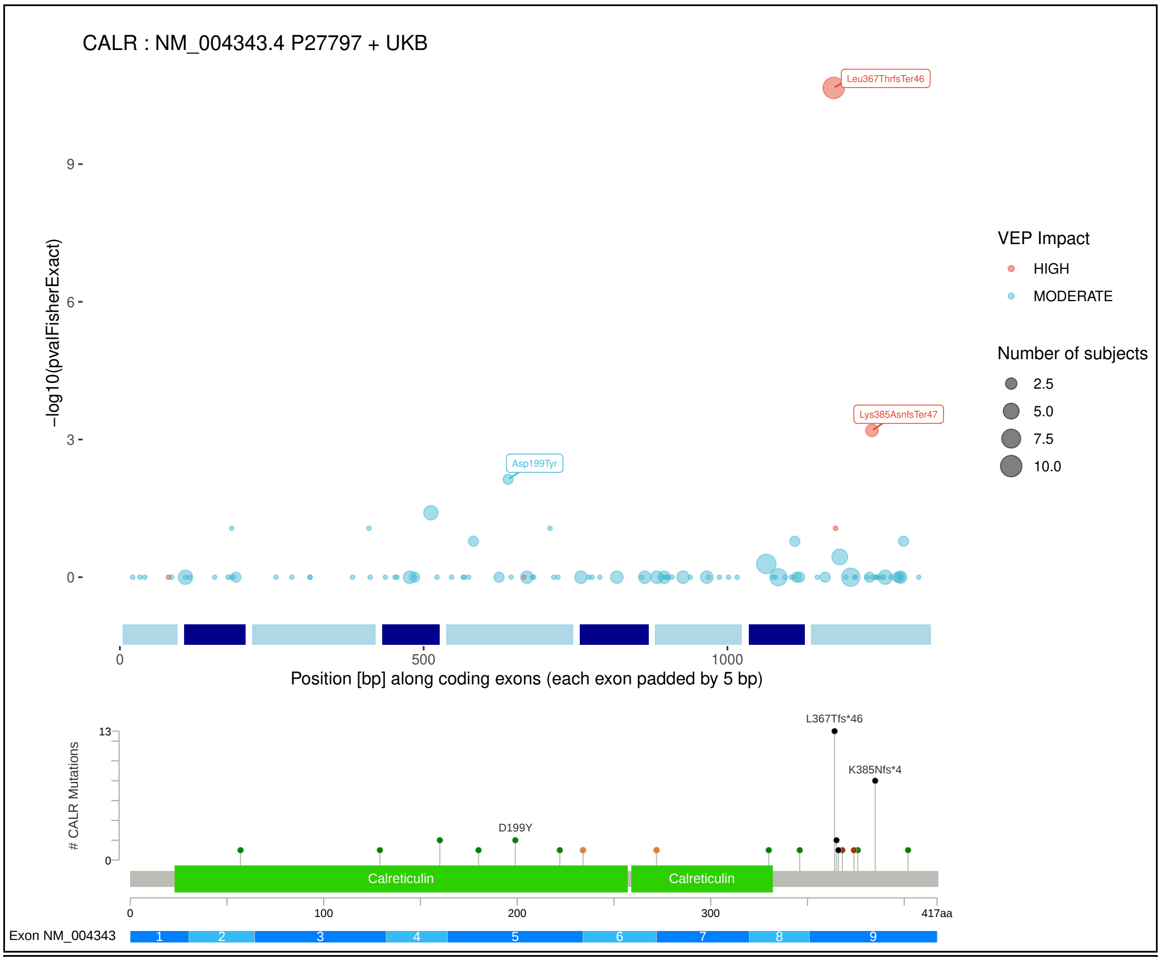

CALR : NM_004343.4 P27797 + UKB
Leu367ThrfsTer46
Lys385AsnfsTer47
Asp199Tyr
−log10(pvalFisherExact)
Position [bp] along coding exons (each exon padded by 5 bp)
VEP Impact
HIGH
MODERATE
Number of subjects
2.5
5.0
7.5
10.0
CALR Mutations
L367Tfs*46
K385Nfs*4
D199Y
Calreticulin
Calreticulin
417aa
Exon NM_004343

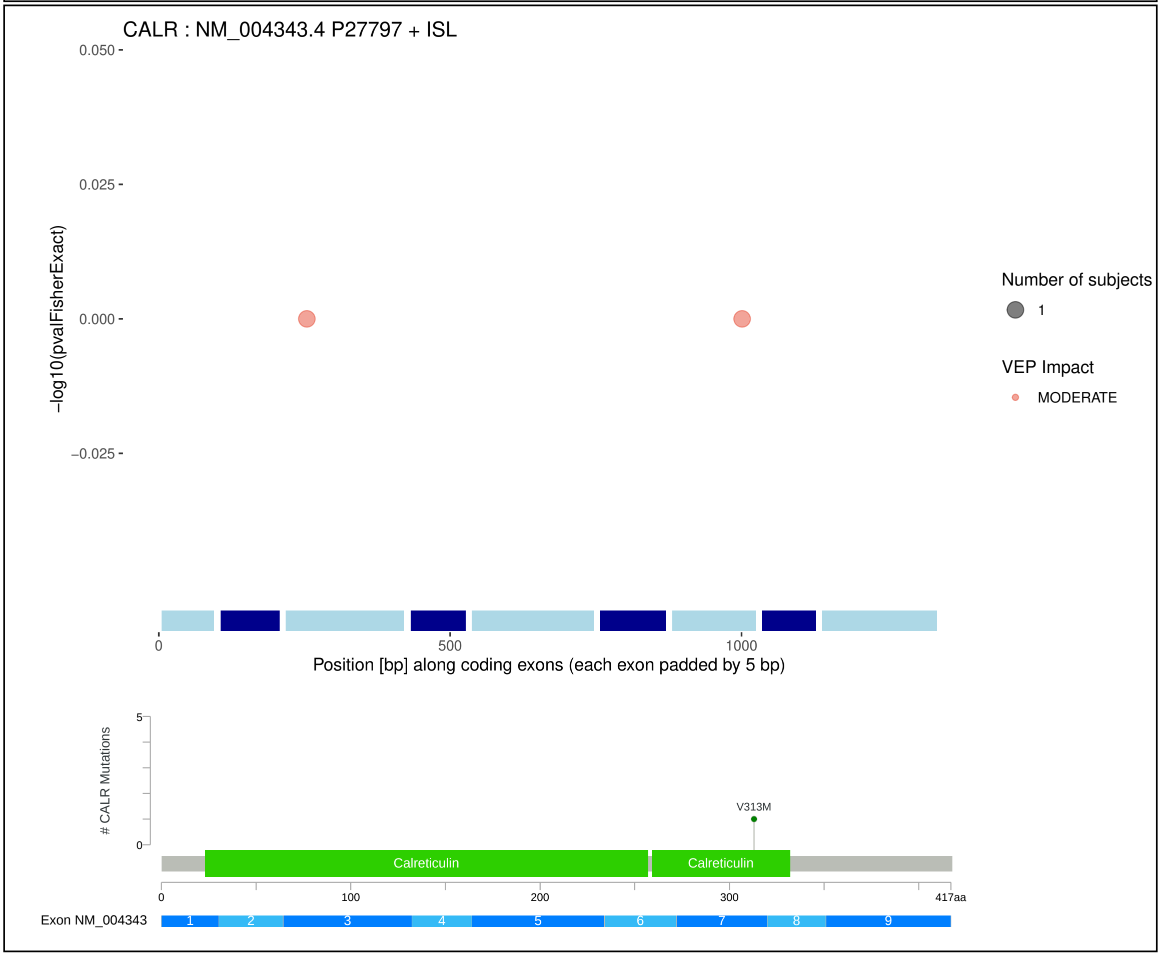

CALR : NM_004343.4 P27797 + ISL
−log10(pvalFisherExact)
Position [bp] along coding exons (each exon padded by 5 bp)
Number of subjects
1
VEP Impact
MODERATE
CALR Mutations
V313M
Calreticulin
Calreticulin
417aa
Exon NM_004343

Panel Q

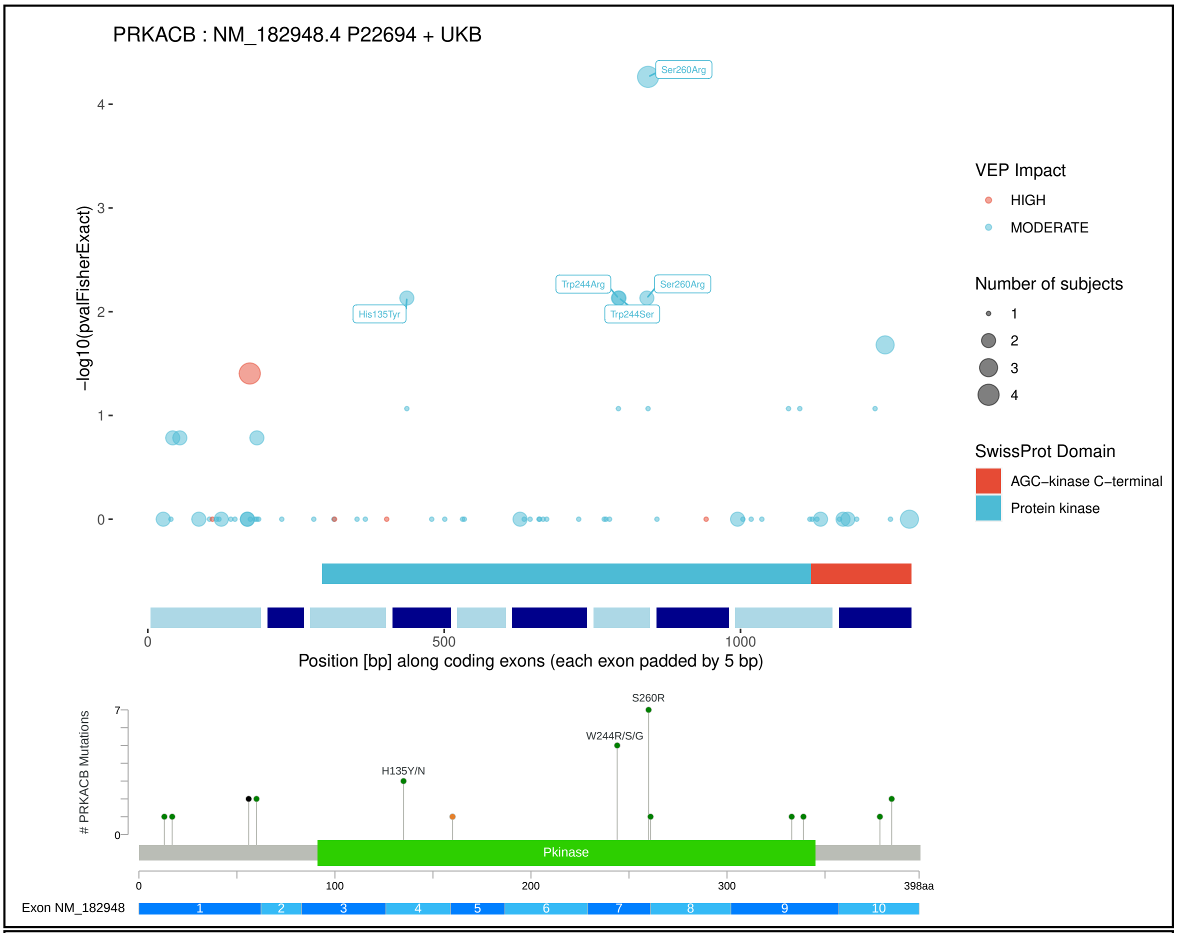
PRKACB : NM_182948.4 P22694 + UKB
Ser260Arg
His135Tyr
Trp244Arg
Ser260Arg
Trp244Ser
−log10(pvalFisherExact)
Position [bp] along coding exons (each exon padded by 5 bp)
VEP Impact
HIGH
MODERATE
Number of subjects
1
2
3
4
SwissProt Domain
AGC−kinase C−terminal
Protein kinase
PRKACB Mutations
S260R
W244R/S/G
H135Y/N
Pkinase
398aa
Exon NM_182948

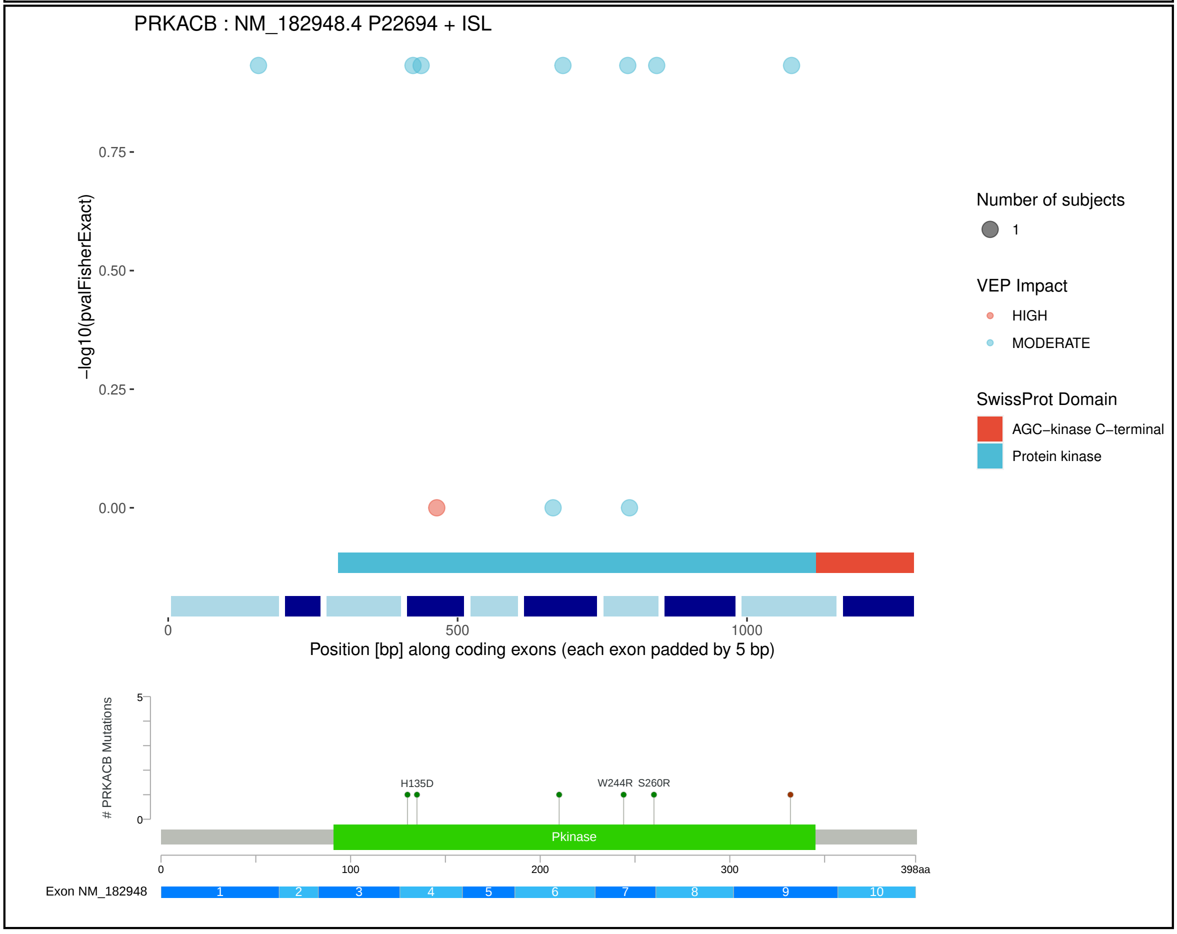
PRKACB : NM_182948.4 P22694 + ISL
−log10(pvalFisherExact)
Number of subjects
1
VEP Impact
HIGH
MODERATE
SwissProt Domain
AGC−kinase C−terminal
Protein kinase
Position [bp] along coding exons (each exon padded by 5 bp)
PRKACB Mutations
H135D
W244R
S260R
Pkinase
398aa
Exon NM_182948

Panel R

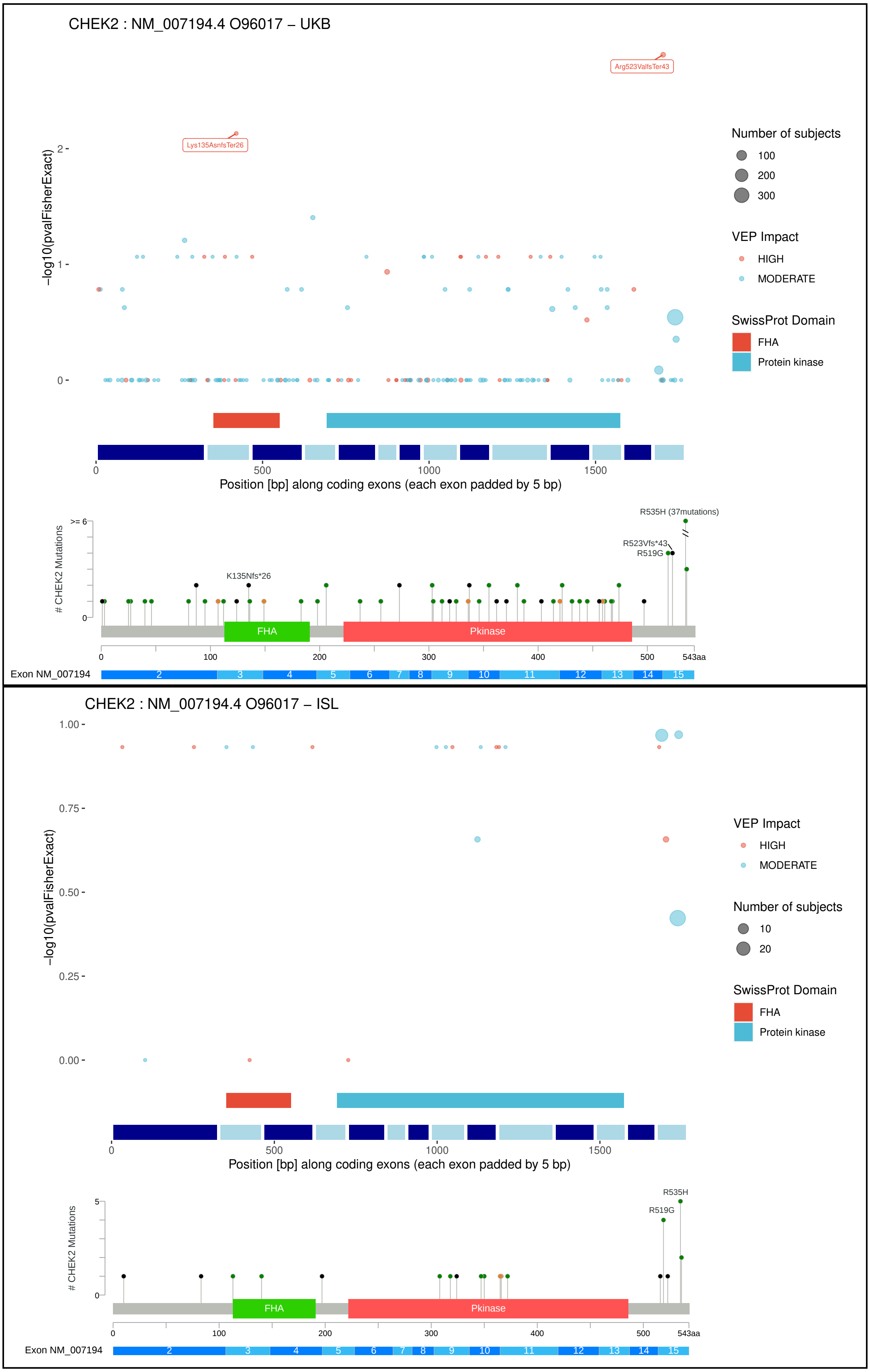
CHEK2 : NM_007194.4 O96017 – UKB
Arg523ValfsTer43
Lys135AsnfsTer26
–log10(pvalFisherExact)
Number of subjects
100
200
300
VEP Impact
HIGH
MODERATE
SwissProt Domain
FHA
Protein kinase
Position [bp] along coding exons (each exon padded by 5 bp)
R535H (37mutations)
R523Vfs*43
R519G
K135Nfs*26
CHEK2 Mutations
FHA
Pkinase
543aa
Exon NM_007194
CHEK2 : NM_007194.4 O96017 – ISL
–log10(pvalFisherExact)
VEP Impact
HIGH
MODERATE
Number of subjects
10
20
SwissProt Domain
FHA
Protein kinase
Position [bp] along coding exons (each exon padded by 5 bp)
R535H
R519G
CHEK2 Mutations
FHA
Pkinase
543aa
Exon NM_007194

Panel S

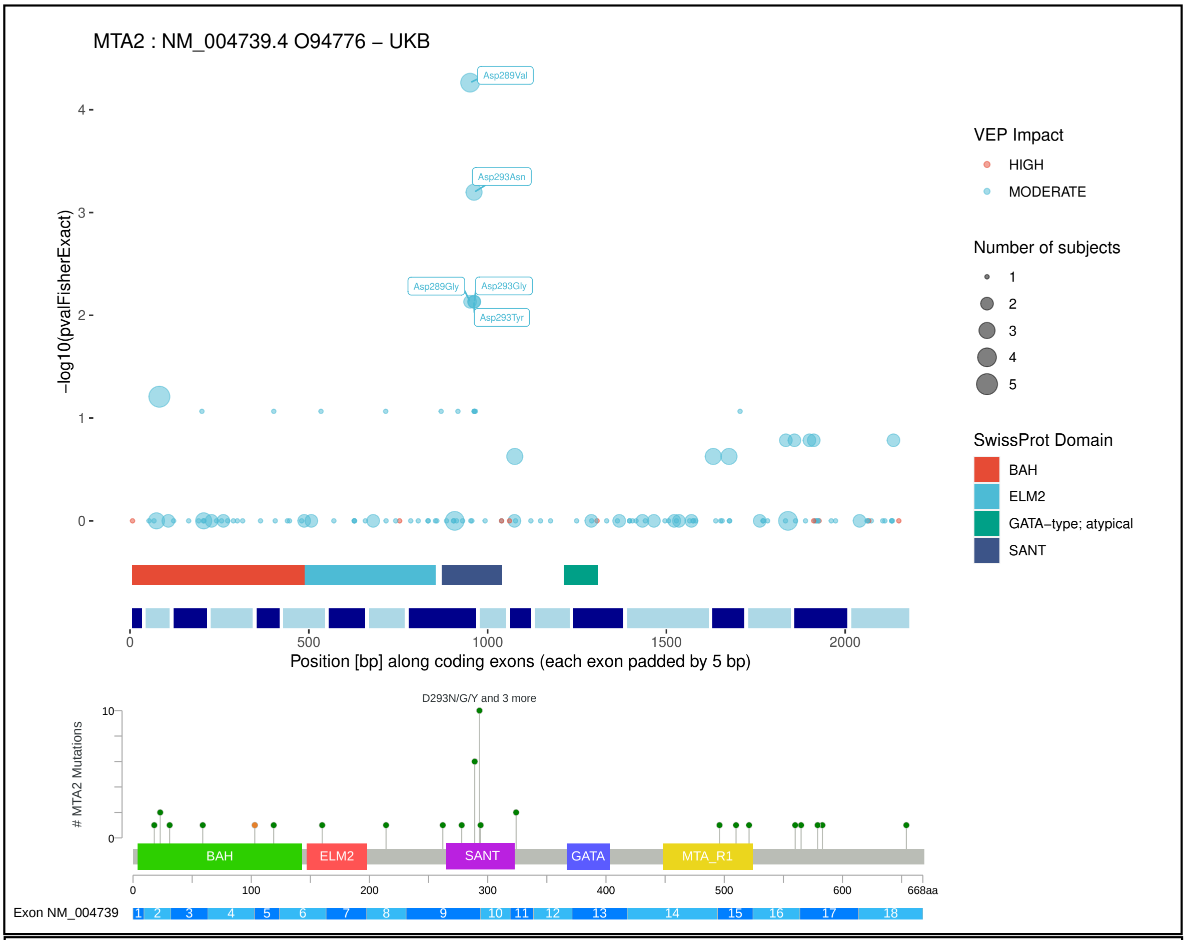
MTA2 : NM_004739.4 O94776 – UKB
Asp289Val
Asp293Asn
Asp289Gly
Asp293Gly
Asp293Tyr
−log10(pvalFisherExact)
VEP Impact
HIGH
MODERATE
Number of subjects
1
2
3
4
5
SwissProt Domain
BAH
ELM2
GATA−type; atypical
SANT
Position [bp] along coding exons (each exon padded by 5 bp)
D293N/G/Y and 3 more
MTA2 Mutations
BAH
ELM2
SANT
GATA
MTA_R1
668aa
Exon NM_004739

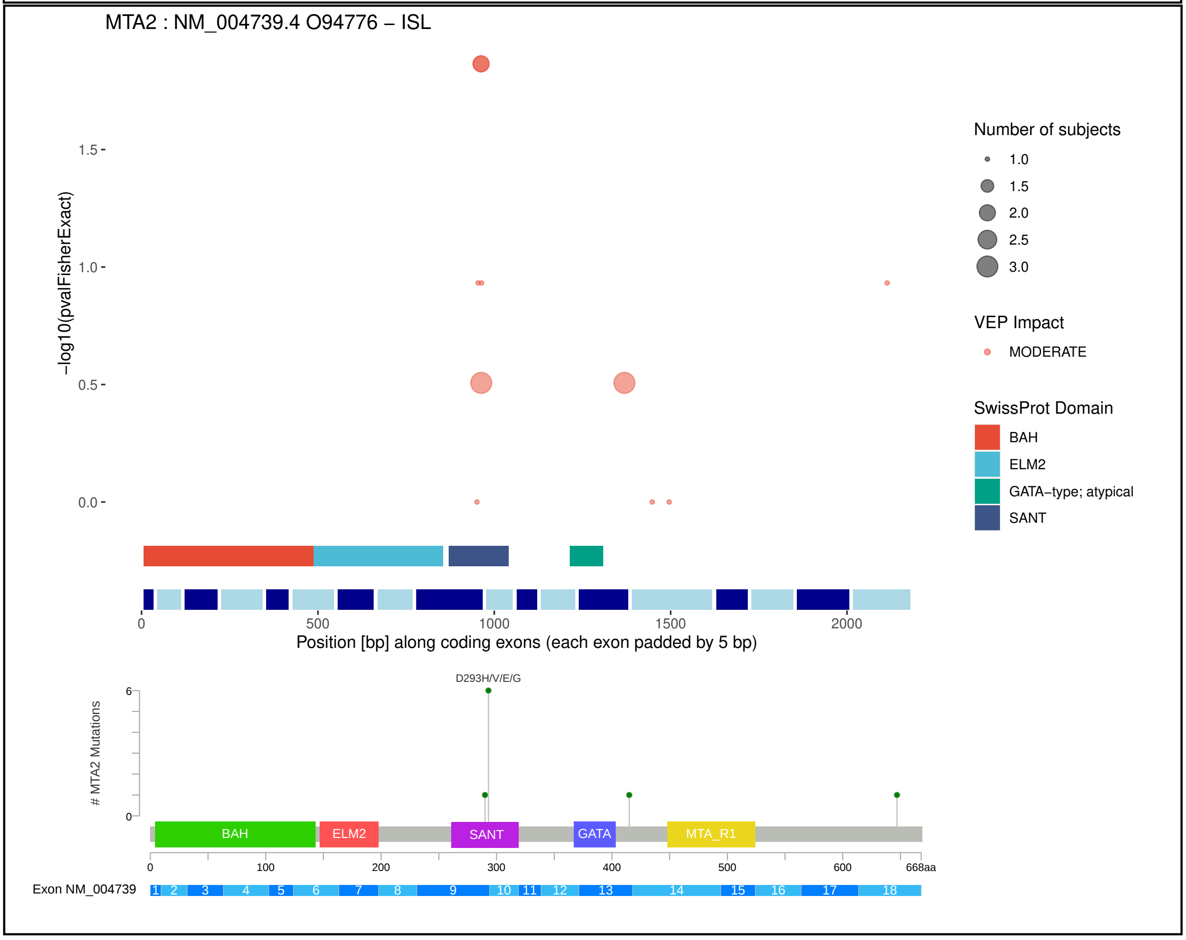
MTA2 : NM_004739.4 O94776 – ISL
−log10(pvalFisherExact)
Number of subjects
1.0
1.5
2.0
2.5
3.0
VEP Impact
MODERATE
SwissProt Domain
BAH
ELM2
GATA−type; atypical
SANT
Position [bp] along coding exons (each exon padded by 5 bp)
D293H/V/E/G
MTA2 Mutations
BAH
ELM2
SANT
GATA
MTA_R1
668aa
Exon NM_004739

Supplementary Figure 1 | Association of somatic mutations with CH.
The upper section of each box shows a plot of Fisher Exact association test results for individual mutations in the gene indicated. The y-axis corresponds to the -$\log_{10}$ P-values. Diameter of the circles indicates the total number of subjects with the mutation (CH cases + controls). SwissProt domain and exon structure of the gene shown below the plot. The lower section of each box shows Lollipop plots of the counts of somatic mutations seen in CH cases. Green lollipops are missense, black are frameshifts and orange are splice mutations. PFAM domain and exon structures are shown below each plot. For each gene, data from UKB and ISL are shown on separate plots.
In addition to what is mentioned in the main text, we note that the pattern of mutations in *TET2* was dominated by nonsense/frameshift mutations spread throughout the gene (Panel B). Nevertheless there were recurrent missense mutations concentrated in or near the Tet_JBP Oxygenase domain, notably at Ile1873 ($P=4.0 \times 10^{-7}$ for Ile1873Thr, UKB, FE). For *JAK2*, the CH association was entirely driven by Val617Phe ($P=1.9 \times 10^{-32}$, UKB, FE, Panel E) which has a known, strong association with Myeloproliferative Neoplasia (MPN)[1].
We note that germline *CHEK2* mutations predispose to MPN and CLL[2-4], but somatic *CHEK2* mutations are not well recognized as CH drivers. We saw CH associations with both high and moderate impact somatic mutations in *CHEK2* in both ISL and UKB (Panel R and Supplementary Table 5). The individual mutations giving the strongest signals were the frameshifts Arg523ValfsTer43 and Lys135AsnfsTer26. These are different from the germline CH predisposition variant (Thr367MetfsTer15 [i.e. 1100delC]) that we found in the CH GWAS and they occurred in different individuals.

Supplementary Figure References

1. Szybinski, J. & Meyer, S. C. Genetics of Myeloproliferative Neoplasms. *Hematol. Oncol. Clin. North Am.* **35**, 217–236 (2021).

2. Hinds, D. A. *et al.* Germ line variants predispose to both JAK2 V617F clonal hematopoiesis and myeloproliferative neoplasms. *Blood* **128**, 1121–1128 (2016).

3. Janiszewska, H. *et al.* A risk of essential thrombocythemia in carriers of constitutional CHEK2 gene mutations. *Haematologica* **97**, 366–370 (2012).

4. Rudd, M. F., Sellick, G. S., Webb, E. L., Catovsky, D. & Houlston, R. S. Variants in the ATM-BRCA2-CHEK2 axis predispose to chronic lymphocytic leukemia. *Blood* **108**, 638–644 (2006).
