# Supplementary material for: Genetics and epidemiology of mutational barcode-defined clonal hematopoiesis
Source: Nat Genet. 2023 Nov 6;55(12):2149–59. doi: 10.1038/s41588-023-01555-z (PMC10703693; doi:10.1038/s41588-023-01555-z)
Supplement: Supplementary file 2 — Reporting Summary [file 41588_2023_1555_MOESM2_ESM.pdf]

nature portfolio

Corresponding author(s): Stacey, SN

Last updated by author(s): Sep 22, 2023

Reporting Summary

Nature Portfolio wishes to improve the reproducibility of the work that we publish. This form provides structure for consistency and transparency in reporting. For further information on Nature Portfolio policies, see our Editorial Policies and the Editorial Policy Checklist.

Statistics

For all statistical analyses, confirm that the following items are present in the figure legend, table legend, main text, or Methods section.

n/a	Confirmed	
☐	☒	The exact sample size (*n*) for each experimental group/condition, given as a discrete number and unit of measurement
☒	☐	A statement on whether measurements were taken from distinct samples or whether the same sample was measured repeatedly
☐	☒	The statistical test(s) used AND whether they are one- or two-sided<br>*Only common tests should be described solely by name; describe more complex techniques in the Methods section.*
☐	☒	A description of all covariates tested
☐	☒	A description of any assumptions or corrections, such as tests of normality and adjustment for multiple comparisons
☐	☒	A full description of the statistical parameters including central tendency (e.g. means) or other basic estimates (e.g. regression coefficient) AND variation (e.g. standard deviation) or associated estimates of uncertainty (e.g. confidence intervals)
☐	☒	For null hypothesis testing, the test statistic (e.g. *F*, *t*, *r*) with confidence intervals, effect sizes, degrees of freedom and *P* value noted<br>*Give P values as exact values whenever suitable.*
☒	☐	For Bayesian analysis, information on the choice of priors and Markov chain Monte Carlo settings
☒	☐	For hierarchical and complex designs, identification of the appropriate level for tests and full reporting of outcomes
☐	☒	Estimates of effect sizes (e.g. Cohen's *d*, Pearson's *r*), indicating how they were calculated

Our web collection on statistics for biologists contains articles on many of the points above.

Software and code

Policy information about availability of computer code

Data collection	DNA sequence acquisition: bwa mem (v0.7.17), Picard MarkDuplicates(v2.20.3[UKB],v1.117[ISL]), GATK BaseRecalibrator (v4.0.12), GATK IndelRealigner (GATK 2.3-9), GATK ApplyBQSR (v4.0.12), GATK HaplotypeCaller, GATK GenotypeGVCFs (v4.0.12), Strelka2 (v2.9.10), VEP(v100), GraphTyper (v1.4).<br>RNA sequence acquisition: STAR aligner (v2.5.3)
Data analysis	R packages Survival (v3.3-1) and COLOC (v5.2.2)<br>RNA analysis packages: Kallisto (v0.43.1), LeafCutter (v0.2.6)

For manuscripts utilizing custom algorithms or software that are central to the research but not yet described in published literature, software must be made available to editors and reviewers. We strongly encourage code deposition in a community repository (e.g. GitHub). See the Nature Portfolio guidelines for submitting code & software for further information.

Data

Policy information about availability of data

All manuscripts must include a data availability statement. This statement should provide the following information, where applicable:

- Accession codes, unique identifiers, or web links for publicly available datasets
- A description of any restrictions on data availability
- For clinical datasets or third party data, please ensure that the statement adheres to our policy

In addition to data presented in Supplementary Tables 1-22, the following new datasets are made available at https://www.decode.com/summarydata/ :
1. Variant level GWAS meta-analysis data for ISL and UKB for barcode-CH and each CPLD-CH type illustrated in Fig. 3.
2. Mutation level counts and Fisher exact results for each somatic mutation tested in ISL and UKB.

WGS, genotype and phenotypic data for UKB subjects can be accessed by approved researchers via the UKB research analysis platform: https://ukbiobank.dnanexus.com/landing . Guidance on access can be found here: Apply for access (ukbiobank.ac.uk) . Individual level ISL WGS, RNAseq and phenotype data cannot be made publicly available because that is prohibited by the Icelandic Act on Data Protection and Processing of Personal Data and conditions set forth to us by the Icelandic Data Protection Authority. On-site access to the data at deCODE genetics facilities may be granted. Interested parties should write to the Lead Contact author, S.N.S. (simon.stacey@decode.is) with a brief description of the requirements and intended use. Requests will be discussed by the deCODE data access committee and a response given within four weeks.

We used data from the following public domain sources:
1. GWAS Catalog(Sollis et al. 2023) (https://www.ebi.ac.uk/gwas/home 26/10/2021 release) for reported GWAS associations.
2. GTEx v8(GTEx Consortium 2020) (https://gtexportal.org/home/) for eQTL/sQTL, various tissues.
3. eQTL Catalogue(Kerimov et al. 2021) (https://www.ebi.ac.uk/eqtl) for eQTL/sQTL, various tissues.
4. GEUVADIS(Lappalainen et al. 2013) (https://www.cnag.crg.eu/projects/geuvadis) for eQTL/sQTL in LCL.
5. Chen et al.(Chen et al. 2016) for eQTL/sQTL in monocytes, neutrophils, T cells.
6. eQTLGen Consortium(Võsa et al. 2021) (https://www.eqtlgen.org) for eQTL/sQTL in blood.
7. Franzen et al.(Franzén et al. 2016) for eQTL/sQTL in vascular and metabolic tissues.
8. xQTL Serve(Ng et al. 2017) (https://mostafavilab.stat.ubc.ca/xQTLServe) for eQTL/sQTL in brain.
9. Lee et al.(M. N. Lee et al. 2014) for eQTL/sQTL in dendritic cells.
10. Zeller et al.(Zeller et al. 2010) for eQTL/sQTL in monocytes.
11. MuTHER(Grundberg et al. 2012) (http://www.muther.ac.uk) for eQTL/sQTL in adipose, LCL, skin.
12. Strunz et al.(Strunz et al. 2018) for eQTL/sQTL in liver.
13. Hao et al.(Hao et al. 2012) for eQTL/sQTL in lung.
14. Gillies et al.(Gillies et al. 2018) (https://nephqtl.org) for eQTL/sQTL in kidney.
15. Hauberg et al.(Hauberg et al. 2017) (http://icahn.mssm.edu/gwas2genes) for eQTL/sQTL in various tissues.
16. Pala et al.(Pala et al. 2017) for eQTL/sQTL in leukocytes.
17. Yao et al.(Yao et al. 2017) for eQTL/sQTL in blood.
18. Yazar et al.(Yazar et al. 2022) (GEO [https://www.ncbi.nlm.nih.gov/geo] Accession # GSE196830) for eQTL/sQTL in 14 immune cell types.
19. Liang et al.(Liang et al. 2013) for eQTL/sQTL in LCL.

Research involving human participants, their data, or biological material

Policy information about studies with human participants or human data. See also policy information about sex, gender (identity/presentation), and sexual orientation and race, ethnicity and racism.

Reporting on sex and gender	Sex was used as a covariate in analyses where statistically appropriate. Sex information was self-reported.
Reporting on race, ethnicity, or other socially relevant groupings	For UKB, subjects were of self-declared British or Irish ethnicity. For ISL, subjects had genealogical record proven Icelandic ancestry as far back as great-grandparents.
Population characteristics	Subjects were UK residents with British or Irish ancestry or residents of Iceland with Icelandic ancestry. Median age was 58.4 for UKB, 53.0 for ISL.
Recruitment	All subjects were identified through national registers and invited to participate through letters of invitation.
Ethics oversight	UKB: The overall UKB study was authorized by the North West Research Ethics Committee (REC reference number 06/MRE08/65). Genotype and phenotype data for this particular study were obtained and research conducted under the UKB application license number 56270. ISL: The study was authorized by the Icelandic National Bioethics Committee and the Data Protection Authority (License #VSN-16-104). All individuals gave written informed consent.

Note that full information on the approval of the study protocol must also be provided in the manuscript.

Field-specific reporting

Please select the one below that is the best fit for your research. If you are not sure, read the appropriate sections before making your selection.

☒ Life sciences ☐ Behavioural & social sciences ☐ Ecological, evolutionary & environmental sciences

For a reference copy of the document with all sections, see nature.com/documents/nr-reporting-summary-flat.pdf

Life sciences study design

All studies must disclose on these points even when the disclosure is negative.

Sample size	UKB and ISL are strategic scale studies involving population based whole genome sequencing. We used all available, qualifying samples.
Data exclusions	For UKB, only subjects of self-declared British or Irish ethnicity were included. For ISL, subjects were included only if they had genealogical record proven Icelandic ancestry as far back as great-grandparents. This was necessary to avoid stratification and other technical issues. Subjects were excluded from most analyses (unless otherwise specified) if they had a diagnosis of a hematological disorder (ICD10 codes C81-C96, D45-D47) before or within 6 months after blood draw. Subjects were also excluded if they had substantial evidence of abnormality from hematology parameters measured at recruitment (if available). Subjects were excluded if their data failed various quality control checks.
Replication	For the epidemiological aspects, samples from UKB and ISL were analysed separately and cross-checked for agreement. For the somatic and germline genetics aspects, samples were meta-analysed and cross-checked for substantial heterogeneity between cohorts. For RNAseq eQTL and sQTL, findings were only reported if confirmed in publicly available datasets. For proteomics, UKB data were used for discovery and results confirmed using ISL data.
Randomization	Subject recruitment for UKB utilized stratified random sampling. ISL subjects were patients, family members and controls participating in a broad range of disease-focused projects. The range of projects is so extensive that sampling approaches a saturation level population based recruitment of Icelandic adults.
Blinding	Investigators involved in data collection and processing were not aware of the group allocations of the participants.

Reporting for specific materials, systems and methods

We require information from authors about some types of materials, experimental systems and methods used in many studies. Here, indicate whether each material, system or method listed is relevant to your study. If you are not sure if a list item applies to your research, read the appropriate section before selecting a response.

Materials & experimental systems

n/a	Involved in the study	
☒	☐	Antibodies
☒	☐	Eukaryotic cell lines
☒	☐	Palaeontology and archaeology
☒	☐	Animals and other organisms
☒	☐	Clinical data
☒	☐	Dual use research of concern
☒	☐	Plants

Methods

n/a	Involved in the study	
☒	☐	ChIP-seq
☒	☐	Flow cytometry
☒	☐	MRI-based neuroimaging
